# Photocatalytic Bilateral Disulfuration of Thioethers Toward *α*‐Sulfide Disulfides With Antibacterial Activity

**DOI:** 10.1002/advs.202502862

**Published:** 2025-04-26

**Authors:** Qingqiang Tian, Chuxia Wang, Bingrui Liu, Xiangwei Wu, Yahui Li

**Affiliations:** ^1^ Anhui Provincial Key Laboratory of Hazardous Factors and Risk Control of Agri‐food Quality Safety Anhui Agricultural University Hefei 230036 China; ^2^ Hefei National Laboratory for Physical Sciences at the Microscale and CAS Key Laboratory of Mechanical Behavior and Design of Materials Department of Precision Machinery and Precision Instrumentation University of Science and Technology of China Hefei 230026 China

**Keywords:** antibacterial activity, bilateral disulfurating reagent, photocatalysis, thioether C(sp^3^)−H bond polysulfidation, α−sulfide disulfides

## Abstract

*α*−Sulfide disulfides represent valuable motifs in organic and pharmaceutical chemistry. However, the limited availability of synthetic approaches for *α*−sulfide disulfides has impeded progress in this field. In this study, a photocatalytic approach to synthesizing modifiable *α*−sulfide disulfides is presented using accessible sulfides and a bilateral disulfurating reagent. The reaction proceeds under mild conditions and demonstrates broad substrate compatibility, accommodating both aromatic and aliphatic sulfides. Moreover, the synthesized *α*−sulfide disulfides display robust reactivity in subsequent transformations with different electrophiles. Notably, this protocol can also be applied to the modification of polymer matrices. Bioassays further reveal that certain target compounds exhibit significant antibacterial activity against plant pathogens, such as *Xanthomonas oryzae pv. oryzae* (*Xoo*), *Xanthomonas oryzae pathovar oryzicola* (*Xoc*), and *Dickeya zeae* (*D. zeae*).

## Graphical abstract

This study introduces a mild photocatalytic strategy for synthesizing tunable *α*‐sulfide disulfides from readily available sulfides and bilateral disulfurating reagents. The method demonstrates a broad substrate scope across aromatic and aliphatic sulfides, with products exhibiting versatile reactivity in diverse electrophilic transformations and polymer matrix modifications. Notably, certain derivatives display potent antibacterial effects against plant pathogens.

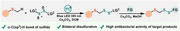

## 1 Introduction

**Figure 1:**
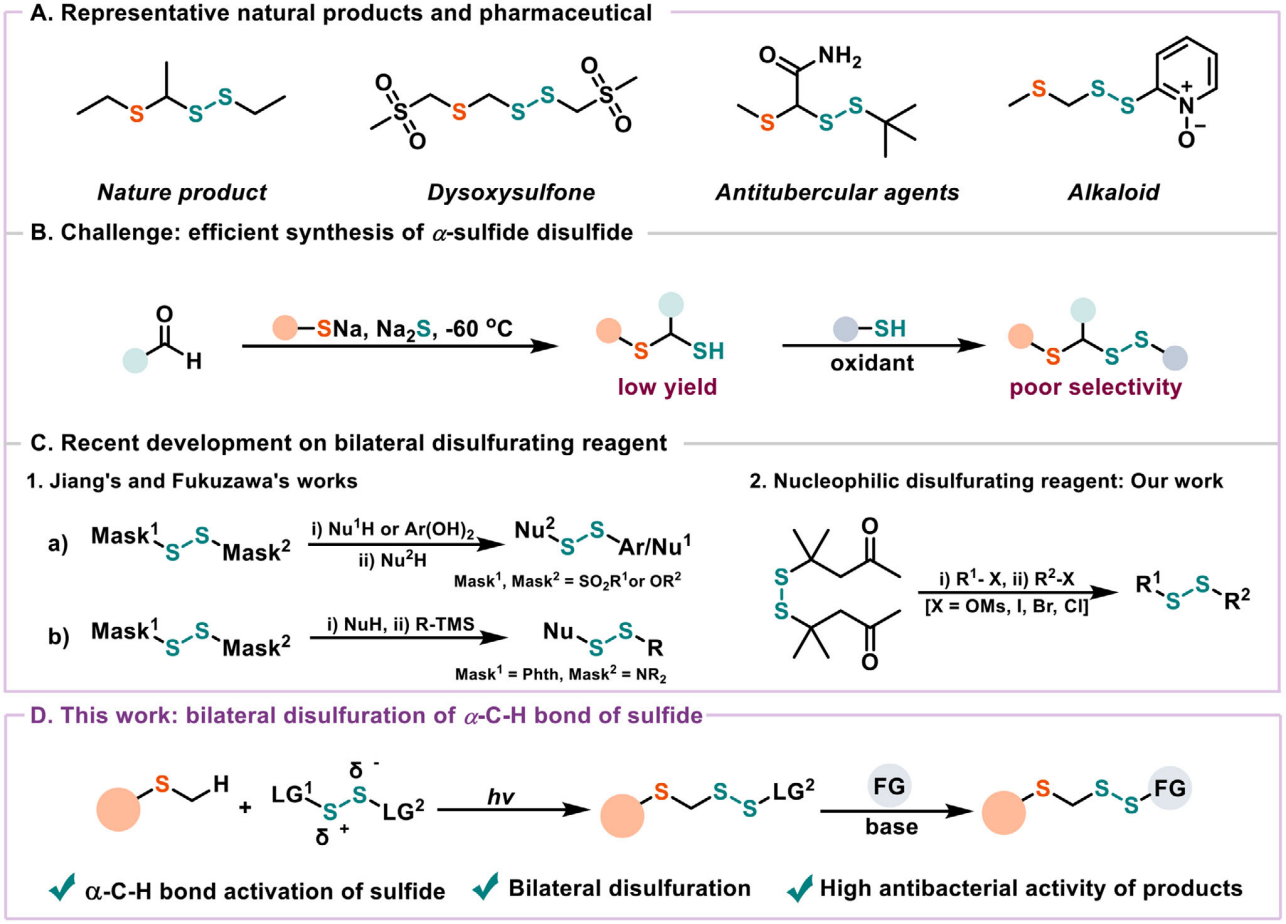
Multisite modifications of natural products. A) Representative SS−containing natural products and pharmaceutical. B) Challenge: efficient synthesis of *α*−sulfide disulfide. C) Recent development on disulfurating reagents. D) This works.

Sulfur is recognized as one of the six fundamental elements indispensable for the sustenance of life.^[^ ^1^ ^]^ In fact, sulfur has a more prominent presence in approved drugs than both fluorine and phosphorus.^[^ ^2^ ^]^ Among organic sulfur compounds, *α*−sulfide disulfides represent privileged structural motifs, as they encompass two important pharmacophores−disulfide and dithioacetal (**Figure** **1**A).^[^ ^3^ ^]^ However, these compounds remain relatively underexamined in biochemistry. In the few studies conducted thus far, *α*−sulfide disulfides exhibited potential against bacteria and fungi,^[^ ^4^ ^]^ such as *Staphylococcus aureus* (*S. aureus*) and *Aspergillus flavus* (*A. flavus*). Moreover, the existence of *α*−sulfide disulfides is also observed in nature.^[^ ^5^ ^]^ Although the value of *α*−sulfide disulfides appears to be profound, the creation of innovative and practical techniques to access these compounds remains challenging. Common protocols for the preparation of *α*−sulfide disulfides usually involve harsh multistep procedures, poor selectivity, and limited substrate universality (Figure 1B).^[^ ^6^ ^]^ Such procedures obviously lag behind the advancement of numerous methods for accessing other sulfur analogues, including sulfides, dithioacetals, and sulfones.

**Figure 2:**
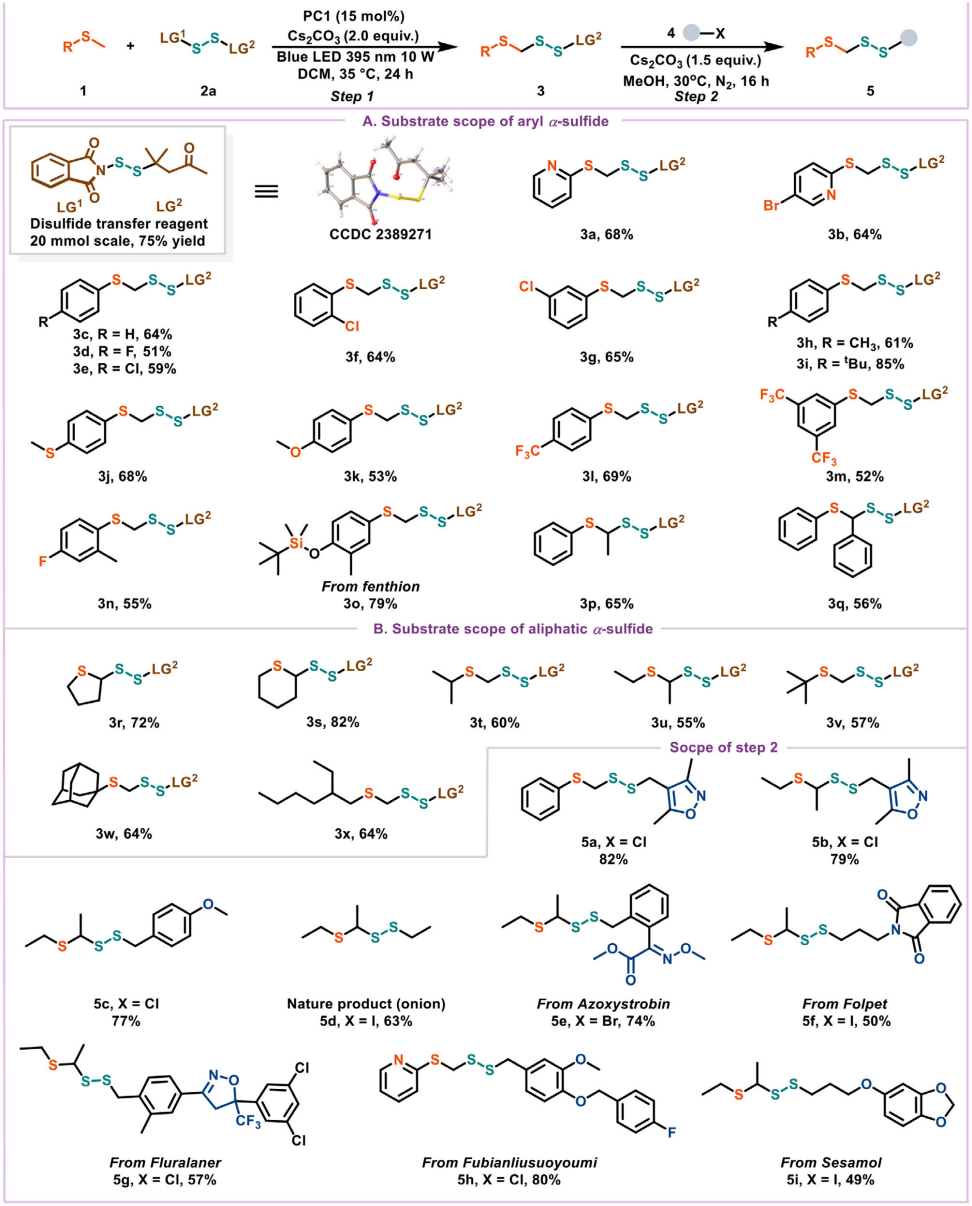
The substrate scope for disulcanization of *α*−sulfide derivatives. Reaction conditions: **1** (0.6 mmol, 3.0 equiv.), **2a** (0.2 mmol, 1.0 equiv.), Cs_2_CO_3_ (0.4 mmol, 2.0 equiv.), **PC1** (15 mol%), DCM (2.0 mL), Blue LED (10 W, 395 nm), 35 °C, 24 h. Isolated yield. **Step 2**: **3** (0.3 mmol, 1.5 equiv.), **4** (0.2 mmol, 1.0 equiv.), Cs_2_CO_3_ (0.3 mmol, 1.5 equiv.), MeOH (2.0 mL), N_2_, 30 °C, 16 h. Isolated yield. The structure of **2a** were determined by X‐ray analysis.^[^ ^13^ ^]^

The prevalence of thioethers in bioactive molecules suggests that direct C─H disulfuration of thioethers would be an ideal approach for synthesizing *α*−sulfide disulfides.^[^ ^7^ ^]^ Ideally, the introduced sulfur−sulfur motif (SS−LG) could be easily modified, which would underscore its potential as a robust and versatile synthetic strategy. However, two fundamental challenges impede progress: i) the thermodynamic instability of sulfur−sulfur bonds compromises selective activation of *α*−C(sp^3^)─H bonds in thioethers; ii) the flexible installation of thioethers and other functional groups on both sides of the sulfur−sulfur bond remains difficult to achieve. With few exceptions, minimal progress on bilateral disulfuration has been achieved.^[^ ^8^ ^]^ Jiang and Fukuzawa pioneered three bilateral disulfurating reagents (RO─SS─OR, RO─SS─SO₂R, Phth─SS‐NR₂) based on bond energy disparities among S─O, S─SO₂R, and S−N bonds (Figure 1C and 1).^[^ ^9^ ^]^ In 2023, our group designed a bilateral disulfide reagent with an alkyl group as the leaving group via *β*−SS elimination. Driven by base, the reagent is used to sequentially disulfurate haloalkanes, constructing unsymmetrical disulfides (Figures 1C and 2).^[^ ^10^ ^]^ Currently, the substrates for bilateral disulfuration reactions are mainly applicable to Ar(OH)₂, NuH (thiols and amines), R−TMS (allyltrimethylsilanes and alkynylsilanes), electron−rich arenes, and activated alkanes. The research on the disulfuration of C(sp^3^)−H bonds using alkanes as substrates remains underexplored. Moreover, the inherent lability of the sulfur−sulfur bond exacerbates the challenge of accomplishing the selective disulfuration of the *α*−C(sp^3^)−H bond in thioethers.

To address this unresolved challenge, we anticipate that combining the Happer reagent with our reagent (DSMO) can yield a bilateral disulfurating reagent with both nucleophilic and electrophilic properties. Although this compound **2a** has been described in the literature,^[^ ^11^ ^]^ its functional potential as a bifunctional disulfide precursor remains underexplored, with no systematic investigation into its reactivity or stereoselective applications in the synthesis of disulfides. We hypothesized that a photoinduced selective activation of the *α*−C(sp^3^)−H bond in sulfides via hydrogen atom transfer (HAT) would lead to *α*−thiol radical formation.^[^ ^12^ ^]^ Subsequently, a photocatalytic *α*−C(sp^3^)−H disulfuration reaction occurs with the Happer reagent to form a modifiable *α*−sulfide disulfide scaffold. In the presence of a base, “RSCH₂SS^−^” is generated in situ through *β*−elimination, enabling it to react with a wider range of electrophilic reagents (Figure 1D). This optimized approach promotes the synthesis of *α*−sulfide disulfides by leveraging the readily available unactivated C(sp^3^)−H bonds in sulfides, eliminating the need for pre‐functionization reactions.

## 2 Results and Discussion

To test our initial hypothesis, we first conducted a gram−level experiment for preparation of the bilateral disulfide transfer reagent **2a**. As illustrated in **Figure** **2**, we successfully prepared **2a** (CCDC 2 389 271) with a yield of 75% on a 20 mmol scale. Subsequent optimization studies were conducted using 2−methylthiopyridine (**1a**) and the bilateral disulfurating reagent (**2a**) to produce a highly functionalized *α*−sulfide disulfide, with the results detailed in **Table** **1**. Systematic investigations revealed that the reaction achieves optimal efficiency, when using 4,4′−dichloro substituted benzophenone **PC1** (15 mol%) as the photocatalyst and Cs_2_CO_3_ (2.0 equiv.) as the base in DCM, under irradiation with a 10 W 395 nm LED for 24 h at 35 °C, yielding the *α*−sulfide disulfides product **3a** in 71% GC yield (Table 1, entry 1), along with a 15% yield of the by‐product, 4‐methyl‐4‐(((pyridin‐2‐ylthio)methyl)thio)pentan‐2‐one. Control experiments demonstrated the necessity of the photocatalyst, base, and light for this catalytic protocol (Table 1, entries 2−4). Lowering the PC1 loading reduced the reaction rate (Table 1, entry 5). A survey of alternative bases revealed that substituting Cs₂CO₃ with bases such as K₃PO₄, Na₂CO₃, DIPEA, or Et₃N resulted in reduced yields (Table 1, entries 6–9). Furthermore, solvents that play an important role in this reaction, such as DMSO, DMF, and MeCN, were found to be unsuitable for this reaction (Table 1, entries 10–12). We also evaluated various benzophenone photocatalysts with different chromophores, but these yielded lower amounts of the desired product (Table 1, entries 13–18). Finally, changing the irradiation wavelength from 395 to 380 or 425 nm resulted in decreased yields, with **3a** obtained at 43% and trace amounts, respectively (Table 1, entries 19 and 20). Decreased yields were observed at both 25 and 50 °C (Table 1, entries 21 and 22).

**Table 1:** Optimization of the reaction conditions.^a)^

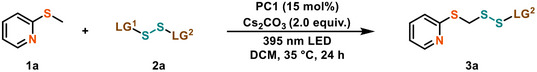						Leaving groups 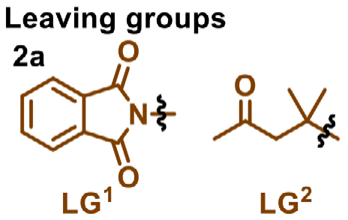
Entry	Variation	Yield of **3a**(%)	Entry	Variation	Yield of **3a**(%)	Photocatalysts (PC) 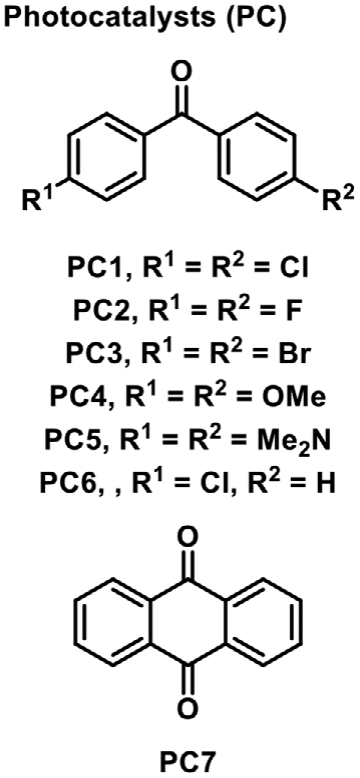
1	none	71(68)^b)^	12	MeCN instead of DCM	0	
2	without photocatalyst	0	13	PC2 instead of PC1	57	
3	without base	0	14	PC3 instead of PC1	55	
4	without light	0	15	PC4 instead of PC1	52	
5	PC1 5%	38	16	PC5 instead of PC1	0	
6	K_3_PO_4_ instead of Cs_2_CO_3_	57	17	PC6 instead of PC1	45	
7	Na_2_CO_3_ instead of Cs_2_CO_3_	44	18	PC7 instead of PC1	Trace	
8	DIPEA instead of Cs_2_CO_3_	0	19	380nm instead of 395nm	43	
9	Et_3_N instead of Cs_2_CO_3_	0	20	425nm instead of 395nm	Trace	
10	DMSO instead of DCM	0	21	25 ^o^C instead of 35^ o^C	52	
11	DMF instead of DCM	0	22	50 ^o^C instead of 35 ^o^C	48	

^a)^ Reaction conditions: **1a** (0.6 mmol, 3.0 equiv.), **2a** (0.2 mmol, 1.0 equiv.), Cs_2_CO_3_ (0.4 mmol, 2.0 equiv.), and **PC1** (15 mol%) in DCM (2.0 mL) under irradiation using blue LEDs (10W, 395 nm) at 35 °C for 24 h. Yields were determined by GC with tetradecane as the internal standard.

^b)^ Isolated yield.

## 3 Reaction Substrate Scope

With the optimized conditions established, we investigated the versatility and efficiency of this protocol in synthesizing *α*−sulfide disulfides. It was found that thioanisole derivatives with various electronic properties and functional groups, including −Br, −*^t^* Bu, and −CF₃, showed excellent reactivity, with yields ranging from 51% to 85% (e.g., **3b**, **3k**, and **3n**). Substrates with chlorine substitutions at the ortho, meta, or para positions underwent disulfuration with comparable yields (**3e**, **3f**, and **3** **g**), indicating that steric factors did not significantly impede reactivity.

Additionally, silicon−containing thioanisole derivatives demonstrated good tolerance (**3o**, 79%), providing opportunities for further synthetic modifications. Site–selectivity was also observed, with exclusive activation at the *S*−methyl position in thioethers with multiple C(sp^3^)─H activation sites (**3p** and **3q**). Meanwhile, unactivated aliphatic substrates, such as linear and cyclic aliphatic thioethers were successfully accommodated and transformed with exclusive selectivity (**3v** and **3w**), the *β*‐branched thioether **1x** was also suitable for this reaction system, and the target compound **3x** was obtained with a yield of 51%.

After the successful execution of the initial disulfurating reactions of thioethers, the scope of the second disulfurating reaction was further investigated. To our delight, the reaction demonstrated a notable capacity to accommodate a wide range of alkyl halides possessing varying electronic properties and functional groups, resulting in the formation of the desired *α*−sulfide disulfide products **5a**–**5c** in yields ranging from 77%–82%. Notably, the natural product 1−(ethyldisulfanyl)−1−(ethylsulfanyl)ethane (**5d**), isolated from durian, was also accessible under standard conditions. The ^1^H NMR and ^13^C NMR spectra are consistent with those reported in the literature.^[^ ^14^ ^]^ Additionally, commercially available drugs such as kresoxim−methyl (**5e**), folpet (**5f**), and fluralaner (**5g**) were effortlessly modified through our reactions, broadening the chemical space available for structure‐activity relationship (SAR) studies. Additionally, sesamol was also tolerated in this reaction, yielding the desired disulfide **5i** with a synthetically useful yield.

## 4 Synthetic Application

**Figure 3:**
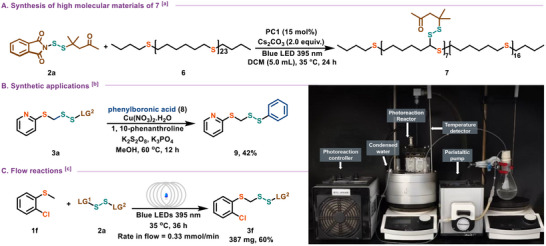
Synthetic applications. A) Synthesis of polymer of **7**; B) Synthetic application; C) Floew reaction. ^[a]^ Reaction conditions: **2a** (0.2 mmol, 1.0 equiv.), **6** (76.0 mg), PC1 (15 mol%), Cs_2_CO_3_ (0.4 mmol, 2.0 equiv.), DCM (5.0 mL), Blue LED 395 nm, 35 °C, 24 h. ^[b]^ Reaction conditions: **3a** (0.3 mmol, 1.5 equiv.), phenylboronic acid (0.2 mmol, 1.0 equiv.), Cu(NO_3_)_2_ **^.^** H_2_O (15 mol%), 1,10−phen (15 mol%), K_2_S_2_O_8_ (0.3 mmol, 1.5 equiv.), K_3_PO_4_ (0.3 mmol, 1.5 equiv.), MeOH (4.0 mL), 60 °C, 12 h. ^[c]^ Reaction conditions: **1f** (6.0 mmol, 3.0 equiv.), **2a** (2.0 mmol, 1.0 equiv.), PC1 (15 mol%), Cs_2_CO_3_ (4.0 mmol, 2.0 equiv.), rate in flow (0.33 mmol min^−1^), DCM (50.0 mL), Blue LED 395 nm, 35 °C, 36 h.

Incorporating sulfur atoms into polymer chains can enhance material properties across multiple applications. To demonstrate the utility of this protocol for materials chemistry, we performed late−stage disulfuration of polysulfide ether substrates relevant to polymer materials. As shown in **Figure** **3**A, thioether polymers successfully underwent sulfurization, yielding target polymer **7** under standard conditions. The second disulfuration process was investigated in the presence of a base to promote the elimination of the masking group, along with alkyl or benzyl halides to alkylate the sulfur. Moreover, phenylboric acid (**9**) is also an effective electrophilic partner (Figure 3B). To further validate the industrial applicability of this catalytic protocol, we conducted a continuous flow synthesis, producing compound **3f** in a 60% yield (387 mg) (Figure 3C).

## 5 Antibacterial Activity Screening

**Figure 4:**
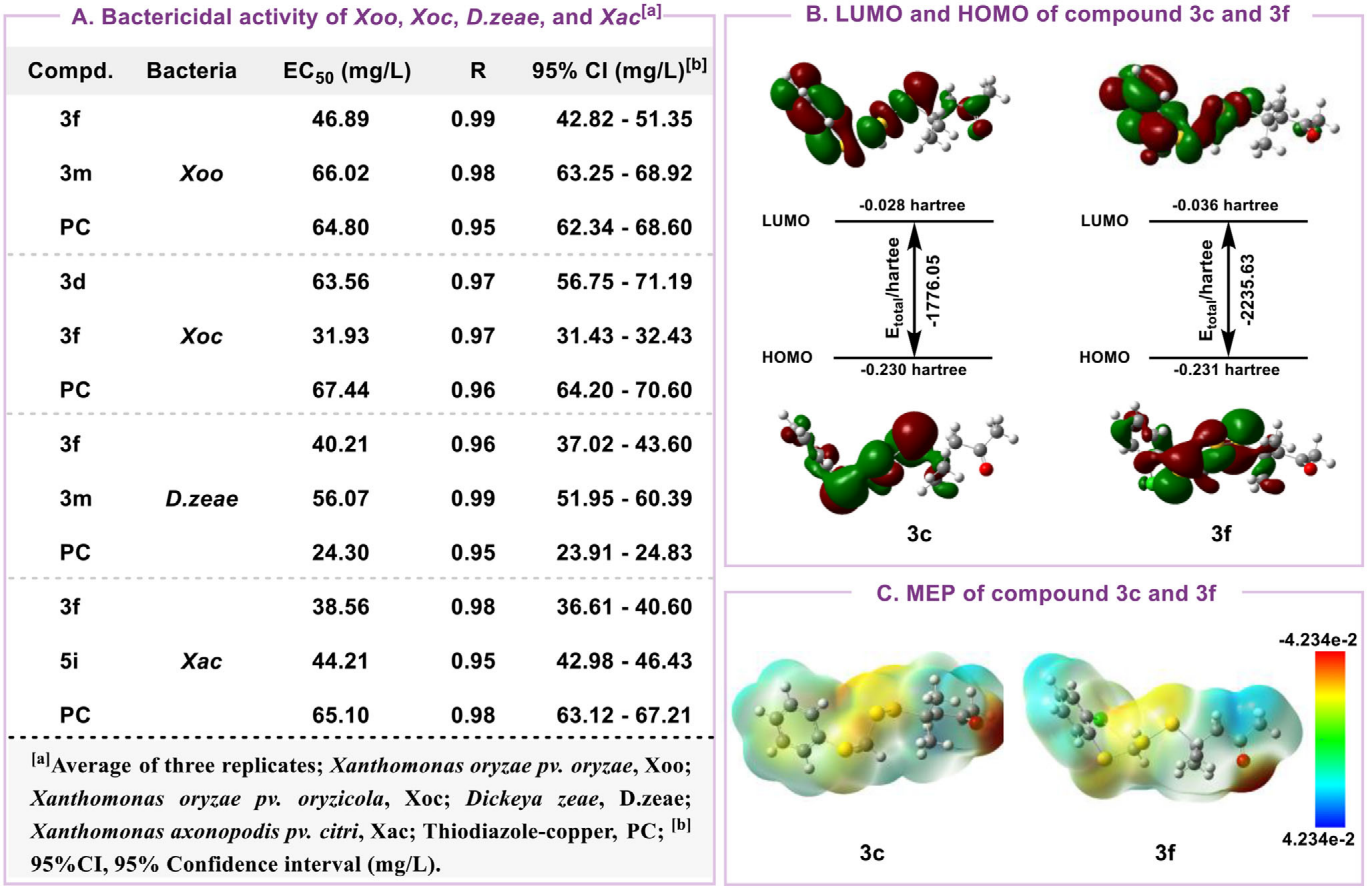
Antibacterial activity screening. A) Bactericidal activity of *Xoo*, *Xoc*, *D. zeae*, and *Xac*. B) HOMO and LUMO of compounds **3c** and **3f**. C) MEP of compounds **3c** and **3f**.

Bacterial diseases of plants represent a significant threat to agricultural production, leading to considerable economic losses globally each year.^[^ ^15^ ^]^ *α*−Sulfide disulfide structures are common cores in numerous antibacterial compounds, prompting interest in their potential application for plant pathogen control. The turbidity method was used to evaluate the bactericidal activity of the 36 newly synthesized *α*−sulfide disulfides against four major bacterial pathogens: *Xanthomonas oryzae pv. oryzae* (*Xoo*),^[^ ^16^ ^]^ *Xanthomonas oryzae pathovar oryzicola* (*Xoc*),^[^ ^17^ ^]^ *Dickeya zeae* (*D. zeae*),^[^ ^18^ ^]^ and *Xanthomonas axonopodis pv. citri* (*Xac*).^[^ ^19^ ^]^ The specific evaluation method is provided in the Supporting Information, with commercial pesticide thiodiazole copper used as a positive control. The evaluation results are presented in Tables S3 and S4 (Supporting Information), when the pyridine ring is substituted with a bromine atom, it enhances the bactericidal activity against *Xoc* (**3b** > **3a**). Furthermore, the presence of a chlorine substituent on the benzene ring, particularly in the ortho position, significantly improves bactericidal activity. For example, compound **3f** exhibited bactericidal activities of 98.8%, 100%, and 97.3% against *Xoo*, *Xoc*, and *Xac* at 100 mg L^−1^, respectively, with EC_50_ values of 46.89, 31.93, and 38.56 mg L^−1^, outperforming thiodiazole copper (64.80, 67.44, and 65.10 mg L^−1^) (Figure 4A). Additionally, *α*‐positioned aryl substitution of the thioether showed superior activity compared to alkyl substitution (**3p** > **3q**). Alkyl thioethers did not enhance inhibitory activity relative to ortho‐chlorobenzyl thioether. Among bilaterally substituted compounds, the aryl‐substituted disulfide demonstrated improved bactericidal activity against *Xac*, reaching 78.0% at 100 mg L^−1^, which is superior to thiodiazole copper (72.1%). These results indicate that these novel *α*−sulfide disulfides hold promising potential as novel candidates for controlling plant pathogenic bacteria.

To further investigate the structural properties contributing to this antibacterial activity, we performed density functional theory (DFT) calculations using the B3LYP method. Compounds **3c** and **3f** were selected to calculate their frontier molecular orbital energies and molecular electrostatic potentials (MEP). As illustrated in **Figure** **4**, the highest occupied molecular orbital (HOMO) of both **3c** and **3f** mainly exists on the molecular S─S bond and aromatic ring. The energy gaps between the HOMO and the lowest unoccupied molecular orbital (LUMO) were measured at 0.202 Hartree for **3c** and 0.195 Hartree for **3f**. Furthermore, their total energies differ considerably, with **3c** having an energy of ‐1776.05 Hartree and **3f** an energy of ‐2235.63 Hartree (Figure 4B). This suggests that the position and nature of the benzene ring substituents are crucial for bactericidal activity, with compounds exhibiting smaller energy gaps and higher total energies demonstrating enhanced effects. MEP analysis provides further insight into intermolecular interactions. For both compounds **3c** and **3f**, the MEP map shows the aryl and alkyl moieties in the positive potential region (blue) and the S─S bond and thioether group in the negative region (yellow) (Figure 4C). These findings indicate that energy levels, molecular orbital distributions, and MEP characteristics may influence receptor interactions, potentially explaining the observed differences in antibacterial activity.

## 6 Reaction Mechanism

**Figure 5:**
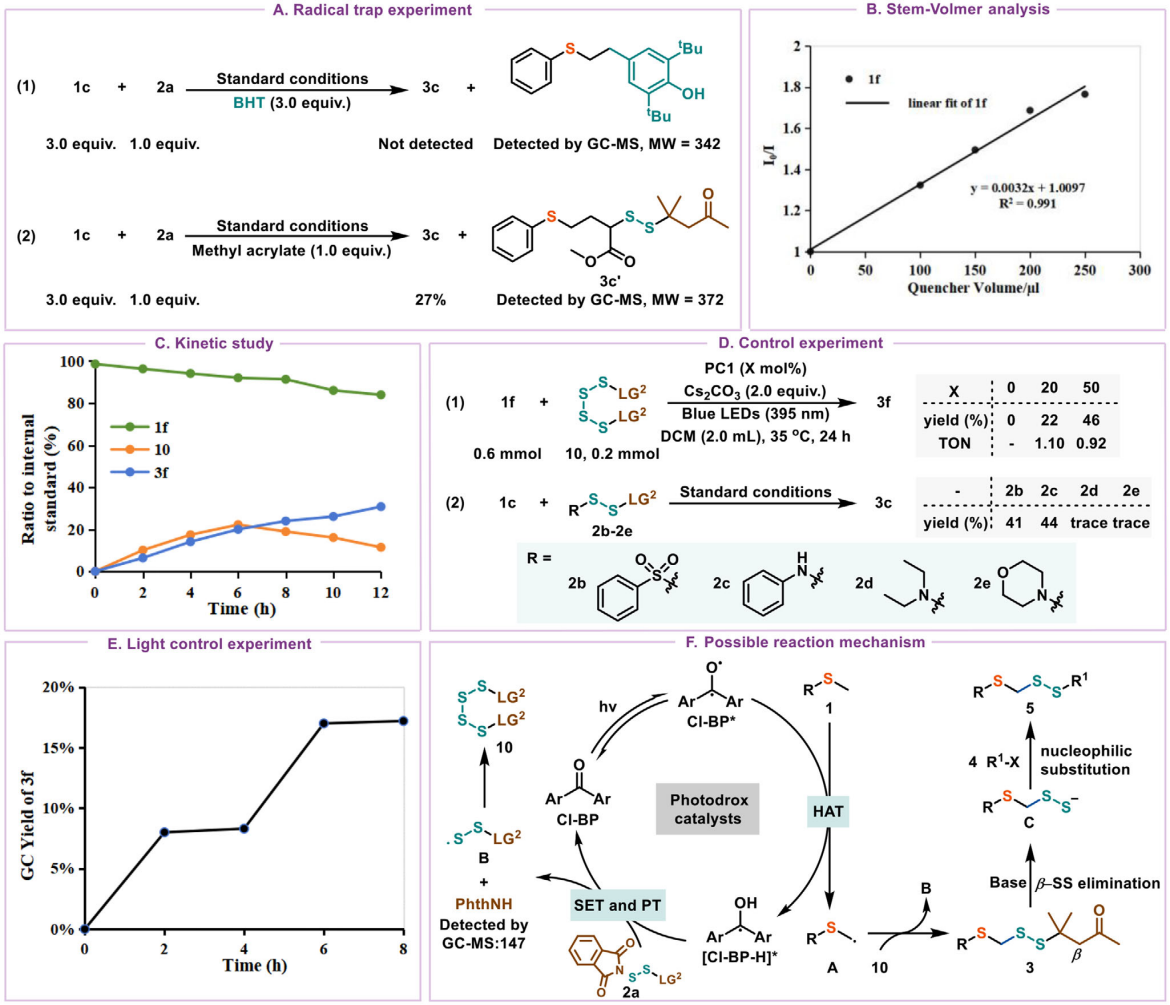
Reaction mechanism. A) Radical trap experiment; B) Stem–Volmer analysis. C) Kinetic study; D) Control experiment. E) Light control experiment. F) Possible reaction mechanism.

Mechanistic investigations and control experiments were conducted to outline a plausible reaction pathway. To further elucidate the reaction mechanism, control experiments were conducted to outline a plausible reaction pathway. First, we examined the role of radical intermediates by using radical scavenger BHT. The reaction was completely inhibited, and no products were formed, GC─MS analysis showed adducts between BHT and thioanisole coupling partners, indicating the formation of an active sulfide alkyl radical (**Figure** **5**A and 1). Moreover, the radical cascade product 3c' was detected when the reaction was performed with methyl acrylate. This result indicates that the reaction system involves a radical pathway. A fluorescence quenching experiment was then conducted to further explore the mechanism. Initially, the emission signal of Cl−BP (4,4′−dichloro−benzophenone) at 433 nm was observed at maximum intensity. However, upon introducing compound **1f**, a quenching of Cl−BP emission occurred at room temperature, suggesting that the reaction of the excited species Cl−BP* with **1f** occurred (Figure 5B). Subsequently, we investigated the kinetics of this transformation by tracking the formation of product **3f** during the initial 12 h (Figure 5C). In the reaction, the dimer (SS−LG^2^)_2_ (**10**) was identified by GC‐MS and HRMS, with its concentration initially increasing and then decreasing. This observation implies that reagent **10** likely serves as a critical mediator in the transformation. Replacing reagent **2a** with **10** revealed a catalyst loading‐dependent product yield (Figure 5D and 1). This may arise from the inability of the intermediate [Cl─BP─H]* to efficiently undergo single‐electron transfer (SET) and proton transfer (PT) processes, when **10** is employed as the disulfurating reagent.^[^ ^20^ ^]^ In contrast, when **2a** was used as the disulfurating reagent, the succinimide radical effectively acted as a reverse hydrogen atom transfer (RHAT) reagent, enabling the photocatalytic cycle.^[^ ^21^ ^]^ DFT calculations were also performed to determine the energy barriers and to verify the feasibility of substitution reactions between **10** and **2a** with benzylthio radicals (Figure S16, Supporting Information). The results revealed that benzylthio radical attack on **10** is more favorable (dimer **10** transition state ΔG^‡^ = 15.7 kcal mol^−1^ vs reagent **2a** transition state ΔG^‡^ = 24.8 kcal mol^−1^), indicating that the pathway involving **10** is energetically preferential for generating the target product. Meanwhile, we also compared several other reagents. The results showed that **2b** and **2c** acted as useful agents, delivering **3c** in 41% and 44% yields, respectively. Conversely, reagents **2d** and **2e** displayed minimal reactivity, yielding only trace amounts of **3c** (Figure 5D and 2). Furthermore, the light on/off experiments demonstrated that continuous light is essential for product formation, effectively ruling out a radical chain process (Figure 5E).

A plausible reaction mechanism is proposed based on our mechanistic studies and relevant literature (Figure 5F).^[10,11a,22]^ Under 395 nm LED illumination, the photosensitizer Cl─BP is excited to its triplet state Cl−BP*. This excited state then generates a double radical, which abstracts a hydrogen atom from the methyl group of **1**, initiating hydrogen atom transfer and forming the **1** methyl radical **A** and [Cl^−1^BP^−1^H]*. In the presence of a base, the hydrogen proton and electron are transferred from PC1−H to reagent **2a**, regenerating the ground−state photosensitizer and producing phthalimide and SS−LG^2^ radicals **B**. These radicals rapidly couple to form the dimer intermediate **10**. Finally, radical **A** is captured either by dimer **10**, resulting in the formation of the target compound **3**. Subsequently, the base abstracts the *α*‐hydrogen of the carbonyl group in compound **3**, triggering *β*‐elimination to form intermediate **B**. The intermediate then undergoes nucleophilic substitution with alkyl halides to afford the *α*−sulfide disulfide derivative **5**.

## 7 Conclusion

In summary, we have developed a novel and efficient protocol for synthesizing *α*−sulfide disulfide derivatives through photocatalytic *α*−C(sp^3^)^−1^H disulfuration of thioethers, employing a bilateral disulfurating reagent. This mild, highly selective process offers a straightforward and robust method for accessing versatile *α*−sulfide disulfides from readily available and environmentally benign thioethers. The resulting compounds exhibit significant potential for further diversification, enabling rapid and orthogonal conversion into various valuable building blocks. Additionally, these synthesized *α*−sulfide disulfides show remarkable antibacterial activity, positioning them as promising candidates for effective plant pathogen control. Given its practicality, convenience, and reliability, this study lays a strong foundation for the further exploration of *α*−sulfide disulfide derivatives across diverse applications.

## 8 Experimental Section

### Preparation of the Reagent 2a

Phthalimide (100 mmol, 1.0 equiv.) and *N*, *N*‐dimethylformamide (80 mL) were added to a 500 mL reaction flask. Disulfide dichloride (8 mL, 0.9 equiv.) was then added, and the reaction was transferred to an oil bath at 28 °C for 20 h. As the reaction proceeds, white solid suspensions gradually form in the reaction solution. After the reaction is completed, ice cream like white solid 2,2′‐disulfanediylbis(isoindoline‐1,3‐dione) (yield: 84%) was obtained through vacuum filtration. Intermediate 2,2′‐disulfanediylbis (isoindoline‐1,3‐dione) (30 mmol, 1.5 equiv.), 4‐mercapto‐4‐methyl‐2‐pentanone (20 mmol, 1.0 equiv.), and 1,2‐dichloroethane (100 mL) were added to a 150 mL reaction flask. The reaction system was placed in an oil bath at 80 °C and stirred for 3 h. After the reaction, the mixture was concentrated, and the residue was purified by silica gel column chromatography to obtain a white solid **2a** (yield: 75%). Store the obtained pure compound at −20 °C. CCDC 2 389 271 contains the supplementary crystallographic data for this paper. These data can be obtained free of charge from The Cambridge Crystallographic Data Centre via www.ccdc.cam.ac.uk/data_request/cif.

### General Procedures for 3

Sulfide **1** (0.6 mmol, 3.0 equiv.), persulfur reagent **2a** (0.2 mmol, 1.0 equiv.), **PC1** (15 mol%), and Cs_2_CO_3_ (0.4 mmol, 2.0 equiv.), dichloromethane (2.0 mL) were added to a 10 mL reaction tube. The tube was then sealed, and the reaction was irradiated under a 395 nm Blue LED light source for 24 h, and the temperature of the reaction solution was controlled at 35 °C through a low‐temperature cooling circulation pump. After TLC monitoring the reaction, DCM was removed by reducing pressure. The target compounds **3** were isolated and purified by silica gel column chromatography.

### General Procedures for 5

**3** (0.3 mmol, 1.5 equiv.), R^2^‐X (**4**, 0.2 mmol, 1.0 equiv.), Cs_2_CO_3_ (0.3 mmol, 1.5 equiv.), and MeOH (2.0 mL) were added to a 10 mL Schlenk tube under nitrogen. The tube was then sealed, and the reaction system was placed in an oil bath at 30 °C and stirred for 16 h. After the reaction (as monitored by TLC), the mixture was concentrated, and the residue was purified by silica gel column chromatography to give the desired product **5**.

### General Procedures for 9

To a 25 mL oven‐dried pressure tube equipped with a magnetic stir bar were added PhB(OH)_2_ (**8**, 0.2 mmol, 1.0 equiv.), **3a** (0.3 mmol, 1.5 equiv.), K_2_S_2_O_8_ (0.3 mmol, 1.5 equiv.), Cu(OAc)_2_.H_2_O (15 mol%), K_3_PO_4_ (0.3 mmol, 1.5 equiv.), 1,10‐phenanthroline (15 mol%), and MeOH (4.0 mL). The sealed pressure tube was vigorously stirred at 60 °C for 12 h. The reaction mixture was quenched with few drops of water and extracted with ethyl acetate (3 × 20 mL). The combined organic layer was dried over anhydrous Na_2_SO_4_, filtered, and concentrated. The residue was purified by chromatography on silica gel to afford the pure product **9**.

### Statistical Analysis

All tests were carried out in triplicate, and data were presented as mean ± standard deviation (SD). Microsoft Office Excel 2019 was used for all antibacterial activity statistical analysis. Modeling, calculation parameter setup for HOMO, LUMO, and MEP were performed using Gauss View 5.0 software, and structural optimization calculations were conducted with Gaussian 09 W software.

## Conflict of Interest

The authors declare no conflict of interest.

## Author Contributions

Q.T. and C.W. contributed equally to this work. Q.T. and Y.L. conceived the project. Y.L. supervised the research. Q.T., C.W., and B.L. conducted the experimental work and analyzed the data. Q.T. and Y.L. were involved in the preparation of the Figures. Q.T., W.X., and Y.L. contributed to the writing and editing of the final manuscript.

## Supporting information



Supporting Information

## Data Availability

The data that support the findings of this study are available in the supplementary material of this article.
