# Supplementary material for: Photocatalytic Bilateral Disulfuration of Thioethers Toward α‐Sulfide Disulfides With Antibacterial Activity
Source: Adv Sci (Weinh). 2025 Apr 26;12(27):2502862. doi: 10.1002/advs.202502862 (PMC12279184; doi:10.1002/advs.202502862)
Supplement: Supplementary file 1 — Supporting Information [file ADVS-12-2502862-s001.pdf]

ADVANCED SCIENCE
Open Access

Supporting Information

for *Adv. Sci.*, DOI 10.1002/advs.202502862

Photocatalytic Bilateral Disulfuration of Thioethers Toward α-Sulfide Disulfides With Antibacterial Activity

Qingqiang Tian, Chuxia Wang, Bingrui Liu, Xiangwei Wu and Yahui Li**

Supporting Information

Photocatalytic Bilateral Disulfuration of Thioethers toward *α*-Sulfide Disulfides with Antibacterial Activity

Qingqiang Tian,[1†] Chuxia Wang,[1†] Bingrui Liu,[1,2] Xiangwei Wu[1*] and Yahui Li[1*]

[1]Anhui Provincial Key Laboratory of Hazardous Factors and Risk Control of Agri-food Quality Safety, Anhui Agricultural University, Hefei 230036, China

[2] Hefei National Laboratory for Physical Sciences at the Microscale and CAS Key Laboratory of Mechanical Behavior and Design of Materials, Department of Precision Machinery and Precision Instrumentation, University of Science and Technology of China, Hefei 230026, China

[*]Corresponding author (E–mail: Yahui.Li@ahau.edu.cn, wxw@ahau.edu.cn)

[†]These authors contributed equally to this work.

Contents

1. General information 3
2. General procedures for the synthesis of starting 5
3. Flow reaction 8
4. Control experiments 9
5. Stern-Volmer fluorescence quenching experiments 12
6. Light control experiments 13
7. Synthesis and characterization of materials 6 and 7 14
8. Evaluation of bactericidal activity 17
9. X-Ray crystallographic data 20
10. DFT calculation 22
11. Characteristic data of compound 31
12. NMR spectra 45

1. General information

Most of the chemicals were purchased from aladdin, energy-chemical, TCI or Alfa Aesar and used as such unless stated otherwise. The reactions were performed on an RLH-18 8-position Photo Reaction System, which manufactured by Beijing Rogertech Co.ltd. NMR spectra were recorded on Bruker Avance 600 and Bruker ARX 400 spectrometers. Chemical shifts (ppm) were given relative to solvent: references for $CDCl_3$ were 7.26 ppm (^1H NMR) and 77.00 ppm (^{13}C NMR); references for DMSO were 2.50 ppm (^1H NMR) and 39.50 ppm (^{13}C NMR). Multiplets were assigned as s (singlet), d (doublet), t (triplet), q (quartet), p (pentet), dd (doublet of doublet), m (multiplet), and br. s (broad singlet). GC-yields were calculated using dodecane as internal standard. All measurements were carried out at room temperature unless otherwise stated. GC-MS analysis was performed on a Shimadzu 2010 instrument and Rtx-5 capillary column. Starting materials were prepared adopting the reported procedures. High resolution mass spectra (HRMS) were recorded on Agilent 6210. The data are given as mass units per charge (m/z). Gas chromatography analysis was performed on a Shimadzu 2010 instrument with an FID detector and Rtx-5 capillary column. The products were isolated from the reaction mixture by column chromatography on silica gel 60, 0.063-0.2 mm, 70-230 mesh (Merck). X-ray single-crystal diffraction data were collected on a Bruker D8 Venture (Bruck, Germany). The crystals were held at 213.00K during data collection. Using Olex2, the structure was solved using a SHELXT structural solver with intrinsic phasing and refinement using the SHELXT refinement package with the least square method. All non-hydrogen atoms were refined with an anisotropy displacement parameter. Hydrogen atoms were placed and observed in geometrically idealized location. Photochemical reactions were performed with a 395 nm LED purchased from Beijing rogertech Ltd. (https://www.rogertech.cn/).

Table S1. Light source detection

Parameter

Name	Value	Name	Value	Name	Value
ESuv(mW/cm²)	0.0030	SDCM	100.00	Peak Signal	53253
Euvc(mW/cm²)	0.0000	Ra	-84.4	Dark Signal	2209
Euvb(mW/cm²)	0.0000	Ee(mW/cm²)	95.83699	Compensate level	2888
Euva(mW/cm²)	69.8753	S/P	57.880		
Euv(mW/cm²)	69.88	Dominant(nm)	415.20		
Eb(mW/cm²)	24.36	Purity(%)	99.9		
Eg(mW/cm²)	0.00	HalfWidth(nm)	15.9		
Er(mW/cm²)	0.00	Peak(nm)	393.7		
Eir(mW/cm²)	4.27	Center(nm)	393.8		
E(lx)	284.98	Centroid(nm)	413.0		
Candle E(fc)	26.48	Color Ratio(RGB)	0.8,0.0,99.2		
CCT(K)	100000	CIE1931 X	13812.099		
Duv	-0.13364	CIE1931 Y	417.252		
CIE x,y	0.1722,0.0052	CIE1931 Z	65961.117		
CIE u,v	0.2535,0.0115	TLCI-2012	0		
CIE u',v'	0.2535,0.0172	Integral Time(ms)	0.1		

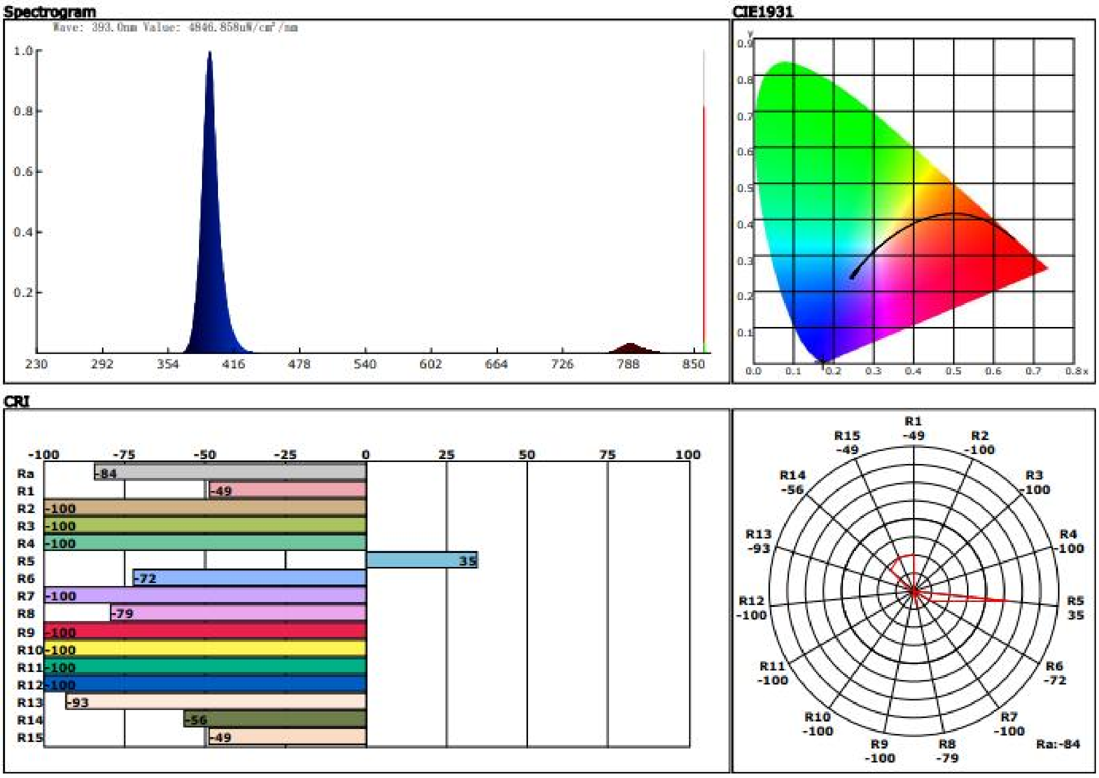

Figure S1. Light source detection.

2. General procedures for the synthesis of starting

Materials preparation of the persulfur reagent (2a)

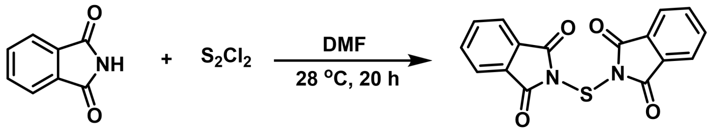

According to the literature[1]: Phthalimide (100 mmol) and *N*,*N*-dimethyl-formamide (80 mL) were added to a 500 mL reaction flask, then add disulfide dichloride (8 mL), and transfer the reaction to an oil bath at 28 °C for 20 h. As the reaction proceeds, white solid suspensions gradually form in the reaction solution. After the reaction is completed, ice cream like white solid 2,2'-disulfanediylbis(isoindoline-1,3-dione) (yield: 84%) is obtained through vacuum filtration.

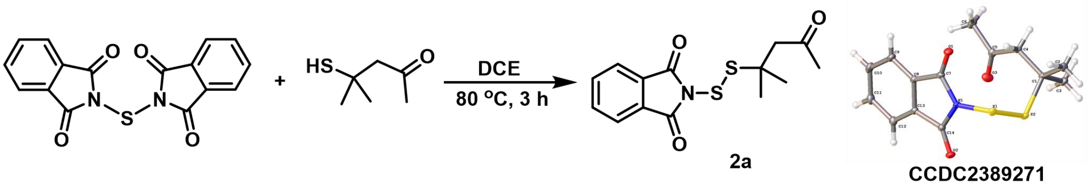

2a CCDC2389271

According to the literature[1]: Intermediate 2,2'-disulfanediylbis (isoindoline-1,3-dione) (30 mmol), 4-mercapto-4-methyl-2-pentanone (2.64 g, 20 mmol), and 1,2-dichloroethane (100 mL) were added to a 150 mL reaction flask. The reaction system was placed in an oil bath at 80 °C and stirred for 3 h. After the reaction, the mixture was concentrated, and the residue was purified by silica gel column chromatography to obtain a white solid **2a** (yield: 75%). Store the obtained pure compound at -20 °C.

Materials preparation of the persulfur reagent (2b)

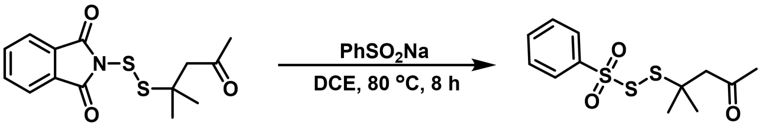

2a 2b

According to the literature[2]: In a 100 mL round-bottomed flask fitted with a reflux condenser, N-phthalimide disulfide **2a** (3.3 mmol) was dissolved in DCE (30 mL) and $PhSO_2Na$ (3.3 mmol) was added in one portion. The reaction mixture was stirred at 80 °C until the consumption of the starting material was observed by TLC. The reaction was cooled to room temperature and filtered through a plug of celite. Solvent was removed and the crude residue was purified by column chromatography to afford the **2b**, yield 71%.

Materials preparation of the persulfur reagent (2c)

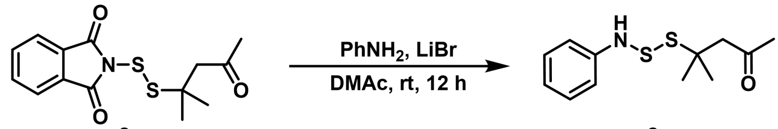

2a 2c

According to the literature[3]: To a mixture of Et_2NH (1.3 mmol, 1.3 equiv.) dissolved in DMAc (10.0 mL) was added **2a** (1.0 mmol, 1.0 equiv.) and LiBr (20%) at rt. After stirring for 12 h at the same temperature, the mixture was concentrated under reduced pressure. The residue was purified by column chromatography to give **2c**, yield 79%.

Materials preparation of the persulfur reagent (2d)

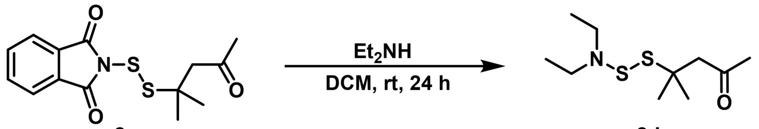

2a 2d

According to the literature[3]: To a mixture of Et_2NH (1.3 mmol, 1.3 equiv.) dissolved in DCM (10.0 mL) was added **2a** (1.0 mmol, 1.0 equiv.) at rt. After stirring for 24 h at rt, the mixture was concentrated under reduced pressure. The residue was purified by column chromatography to give **2d**, yield 72%.

Materials preparation of the persulfur reagent (2e)

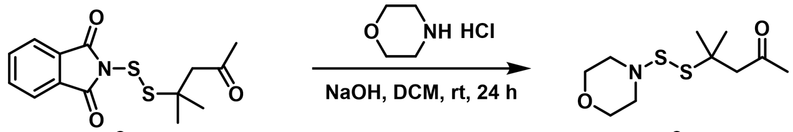

2a 2e

According to the literature[3]: To a mixture of morpholine hydrochloride (1.3 mmol, 1.3 equiv.) and NaOH (1.3 mmol, 1.3 equiv.) dissolved in DCM (10.0 mL) was added **2a** (1.0 mmol, 1.0 equiv.) at rt. After stirring for 24 h at rt, the mixture was concentrated under reduced pressure. The residue was purified by column chromatography to give **2e**, yield 80%.

Materials preparation of the persulfur reagent (10)

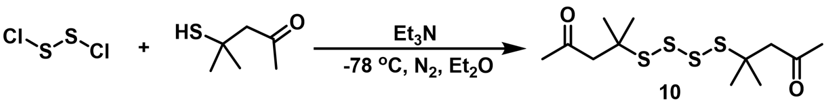

10

According to the literature[4]: Thiol (5.0 mmol, 1.0 equiv.) and Et_3N (, 5.0 mmol, 1.0 equiv.) were added to anhydrous solvent (30 mL) in an oven-dried round bottom flask under nitrogen. The solution was cooled to -78 °C for 1 h, after which sulfur monochloride (3.0 mmol, 0.6 equiv.) was added dropwise. The reaction was stirred for 2 h and then quenched with saturated $NaHCO_3$ solution (30 mL), and the aqueous layer was discarded. The organic layer was washed with deionized water (30 mL) and brine (30 mL). The organic layer was dried over sodium sulfate, the residue was

purified by column chromatography on silica gel using PE/EA = 20:1 and concentrated in vacuo to afford the required compounds (60%).

3. Optimization of conditions

Table S2. Optimization of conditions[a]

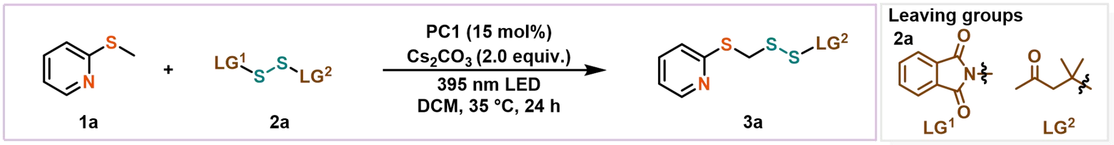

Entry	Variation	Yield of 3a (%)	Entry	Variation	Yield of 3a (%)
1	None	71(68)[b]	12	MeCN instead of DCM	0
2	without photocatalyst	0	13	PC2 instead of PC1	57
3	without base	0	14	PC3 instead of PC1	55
4	without light	0	15	PC4 instead of PC1	52
5	PC1 5%	38	16	PC5 instead of PC1	0
6	K_3PO_4 instead of Cs_2CO_3	57	17	PC6 instead of PC1	45
7	Na_2CO_3 instead of Cs_2CO_3	44	18	PC7 instead of PC1	Trace
8	DIPEA instead of Cs_2CO_3	0	19	380 nm instead of 395 nm	43
9	Et_3N instead of Cs_2CO_3	0	20	425 nm instead of 395 nm	Trace
10	DMSO instead of DCM	0	21	25 °C instead of 35 °C	52
11	DMF instead of DCM	0	22	50 °C instead of 35 °C	48

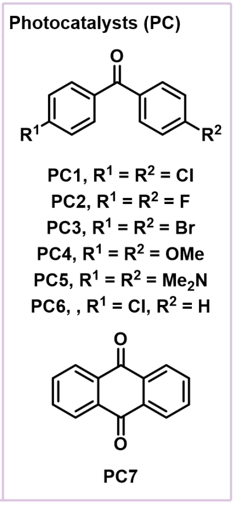

[a] Reaction conditions: **1a** (0.6 mmol, 3.0 equiv.), **2a** (0.2 mmol, 1.0 equiv.), Cs_2CO_3 (0.4 mmol, 2.0 equiv.), and **PC1** (15 mol%) in DCM (2.0 mL) under irradiation using blue LEDs (10W, 395 nm) at 35 °C for 24 h. Yields were determined by GC with tetradecane as the internal standard. [b] Isolated yield.

4. General procedures for 3, 5, and 9

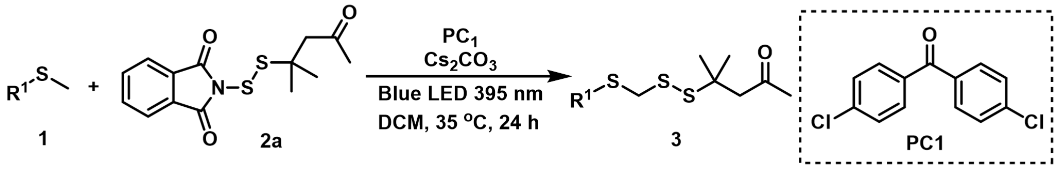

General procedure: Sulfide **1** (0.6 mmol, 3.0 equiv.), persulfur reagent **2a** (0.2 mmol, 1.0 equiv.), PC1 (15 mol%), and Cs_2CO_3 (0.4 mmol, 2.0 equiv.), DCM (2.0 mL) were added to a 10 mL reaction tube. The tube was then sealed, and the reaction was irradiated under a 395 nm Blue LED light source for 24 h, and the temperature of the reaction solution was controlled at 35 °C through a low-temperature cooling circulation pump. After TLC monitoring the reaction, DCM was removed by reducing pressure. The target compounds **3** were isolated and purified by silica gel column chromatography.

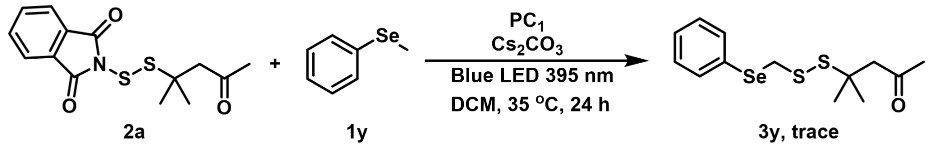

General procedure: **1y** (0.6 mmol, 3.0 equiv.), persulfur reagent **2a** (0.2 mmol, 1.0 equiv.), PC1 (15 mol%), and Cs_2CO_3 (0.4 mmol, 2.0 equiv.), DCM (2.0 mL) were added to a 10 mL reaction tube. The tube was then sealed, and the reaction was irradiated under a 395 nm Blue LED light source for 24 h at 35 °C. After the reaction is complete, the product is detected by GC-MS.

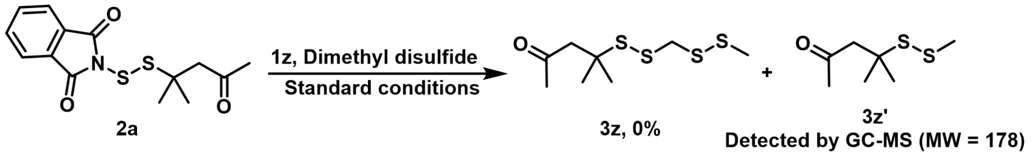

General procedure: **1z** (0.6 mmol, 3.0 equiv.), persulfur reagent **2a** (0.2 mmol, 1.0 equiv.), PC1 (15 mol%), and Cs_2CO_3 (0.4 mmol, 2.0 equiv.), DCM (2.0 mL) were added to a 10 mL reaction tube. The tube was then sealed, and the reaction was irradiated under a 395 nm Blue LED light source for 24 h at 35 °C. After the reaction is complete, the product is detected by GC-MS.

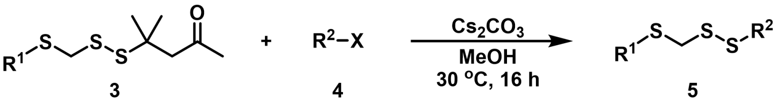

According to the literature[5]: General procedure: **3** (0.3 mmol, 1.5 equiv.), R^2-X (**4**, 0.2 mmol, 1.0 equiv.), Cs_2CO_3 (0.3 mmol, 1.5 equiv.), and MeOH (2.0 mL) were added to a 10 mL Schlenk tube under nitrogen. The tube was then sealed, and the reaction system was placed in an oil bath at 30 °C and stirred for 16 h. After the reaction (as monitored by TLC), the mixture was concentrated, and the residue was purified by silica gel column chromatography to give the desired product **5.**

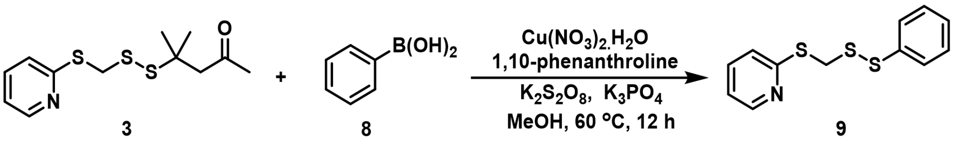

According to the literature[5]: To a 25 mL oven-dried pressure tube equipped with a magnetic stir bar were added $PhB(OH)_2$ (**8**, 0.2 mmol, 1.0 equiv.), **3a** (0.3 mmol, 1.5 equiv.), $K_2S_2O_8$ (0.3 mmol, 1.5 equiv.), $Cu(OAc)_2.H_2O$ (15 mol%), K_3PO_4 (0.3 mmol, 1.5 equiv.), 1,10-phenanthroline (15 mol%), and MeOH (4.0 mL). The sealed pressure tube was vigorously stirred at 60 °C for 12 h. The reaction mixture was quenched with few drops of water and extracted with ethyl acetate (3 × 20 mL). The combined organic layer was dried over anhydrous Na_2SO_4, filtered and concentrated. The residue was purified by chromatography on silica gel to afford the pure product **9.**

3. Flow reaction

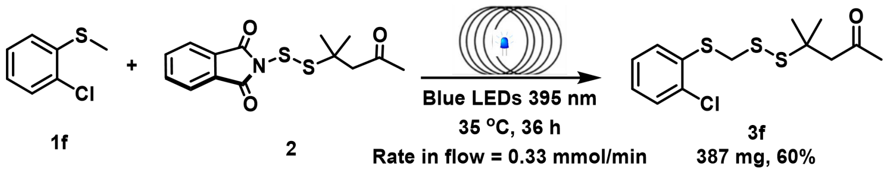

General procedure: Sulfide **1f** (6.0 mmol, 3.0 equiv.), persulfur reagent **2** (2.0 mmol, 1.0 equiv.), PC1 (15 mol%), Cs_2CO_3 (4.0 mmol, 2.0 equiv.), and DCM (50.0 mL) were added to a 150 mL reaction flask. The flask was equipped with rubber plugs, with inlet and outlet of micro tube. The solution was pumped by a pump (0.33 mmol/min) into the micro tube, then returned to flask. This circulatory system was irradiated by Blue LEDs 395 nm under 35 °C for 36 h. After the reaction, ethyl acetate (10 mL) was pumped into the tube to flush out residual fluid. After evaporation of solvent, the residue was purified by column chromatography on silica gel and concentrated in vacuo to afford the required compound **3f** (60%, 0.387 g)

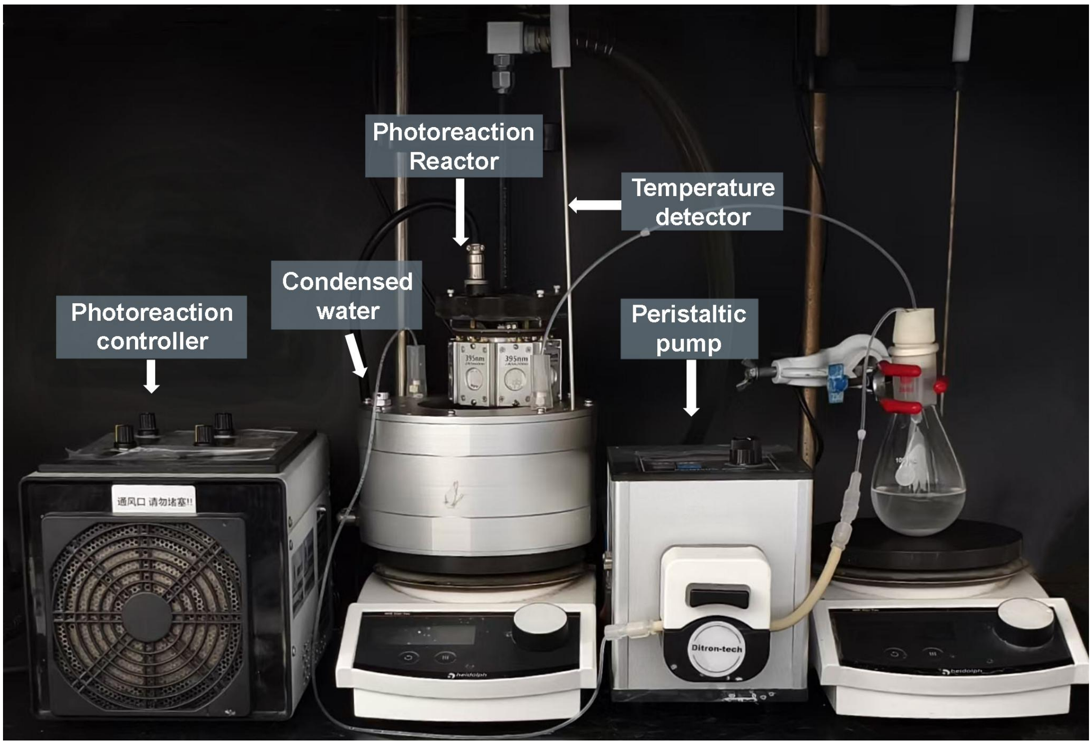

Figure S2. Continuous flow reaction device.

4. Control experiments

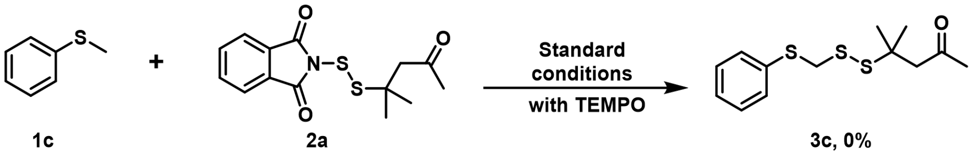

Sulfide **1c** (0.6 mmol, 3.0 equiv.), persulfur reagent **2** (0.2 mmol, 1.0 equiv.), 2,2,6,6-tetramethyl-1-piperinedinyloxy (TEMPO, 0.6 mmol, 3.0 equiv.), PC1 (15 mol%), and Cs_2CO_3 (0.4 mmol, 2.0 equiv.), DCM (2.0 mL) were added to a 10 mL reaction tube. The tube was then sealed, and the reaction was irradiated under a 395 nm light source for 24 h at 35 °C through a low-temperature cooling circulation pump. After the reaction is complete, the product is detected by GC-MS.

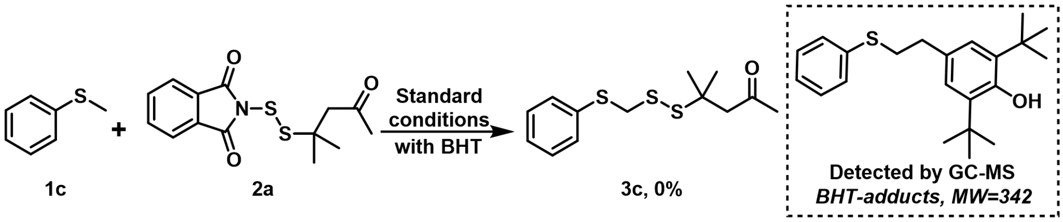

Sulfide **1c** (0.6 mmol, 3.0 equiv.), **2a** (0.2 mmol, 1.0 equiv.), butylated hydroxytoluene (BHT, 0.6 mmol, 3.0 equiv.), PC1 (15 mol%), Cs_2CO_3 (0.4 mmol, 2.0 equiv.), and DCM (2.0 mL) were added to a 10 mL reaction tube. The tube was then sealed, and the reaction was irradiated under a 395 nm light source for 24 h, and the temperature of the reaction solution was controlled at 35 ℃ through a low-temperature cooling circulation pump. After the reaction is complete, the product is detected by GC-MS, GC-MS (EI, 70ev): m/z(%) = 342 (M^+, 24), 233 (10), 219 (100), 57 (22).

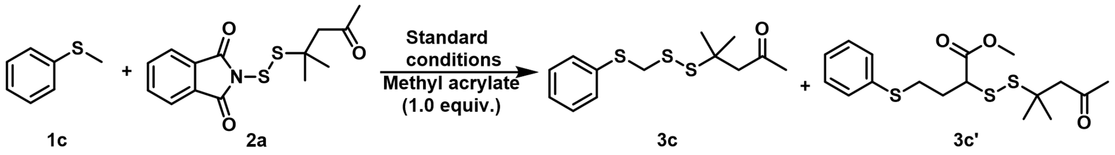

Sulfide **1c** (0.6 mmol, 3.0 equiv.), **2a** (0.2 mmol, 1.0 equiv.), methyl acrylate (0.2 mmol, 1.0 equiv.), PC1 (15 mol%), Cs_2CO_3 (0.4 mmol, 2.0 equiv.), and DCM (2.0 mL) were added to a 10 mL reaction tube. The tube was then sealed, and the reaction was irradiated under a 395 nm light source for 24 h, and the temperature of the reaction solution was controlled at 35 °C through a low-temperature cooling circulation pump. After completion, the product was detected by GC-MS. **3c'** was detected by GC-MS: GC-MS (EI, 70ev) m/z(%) = 372 (M^+, 2), 281 (5), 241 (100), 207 (12), 123 (18), 99 (18).

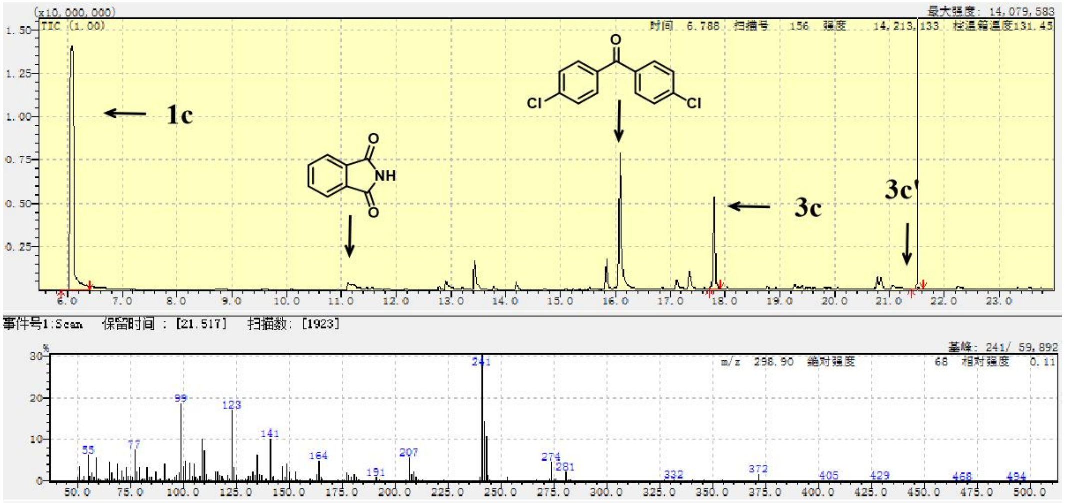

Figure S3. GC-MS of **3c'**

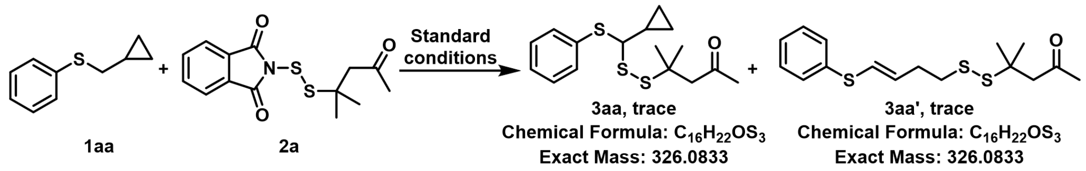

Sulfide **1aa** (0.6 mmol, 3.0 equiv.), **2a** (0.2 mmol, 1.0 equiv.), butylated hydroxytoluene (BHT, 0.6 mmol, 3.0 equiv.), PC1 (15 mol%), Cs_2CO_3 (0.4 mmol, 2.0 equiv.), and DCM (2.0 mL) were added to a 10 mL reaction tube. The tube was then sealed, and the reaction was irradiated under a 395 nm light source for 24 h, and the temperature of the reaction solution was controlled at 35 ℃ through a low-temperature cooling circulation pump. After the reaction is complete, the product is detected by GC-MS.

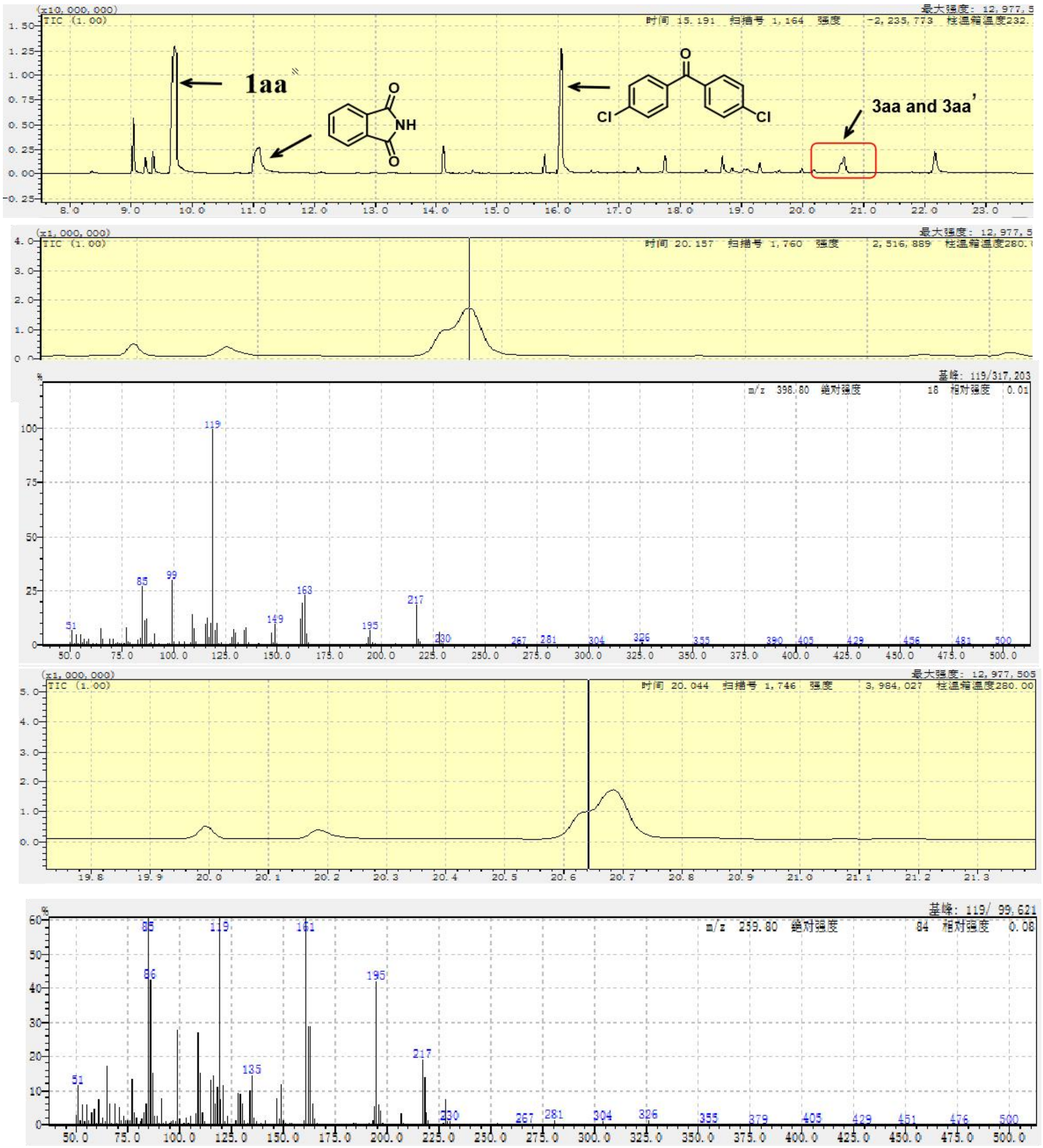

Figure S4. GC-MS of **3aa** and **3aa'**

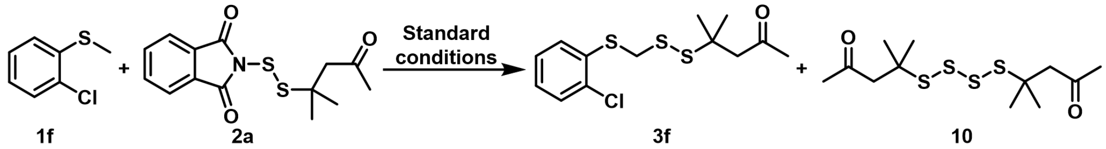

Sulfide **1f** (0.6 mmol, 3.0 equiv.), persulfur reagent **2** (0.2 mmol, 1.0 equiv.), PC1 (15 mol%), and Cs_2CO_3 (0.4 mmol, 2.0 equiv.), DCM (2.0 mL) were added to a 10 mL reaction tube. The tube was then sealed, and the reaction was irradiated under a 395 nm light source for 24 h at 35 °C. After the reaction is complete, the **10** is detected by HRMS. **HRMS (ESI)** calcd for $[M + Na]^+$ ($C_{12}H_{22}O_2S_4Na$): 349.0400; found: 349.0402.

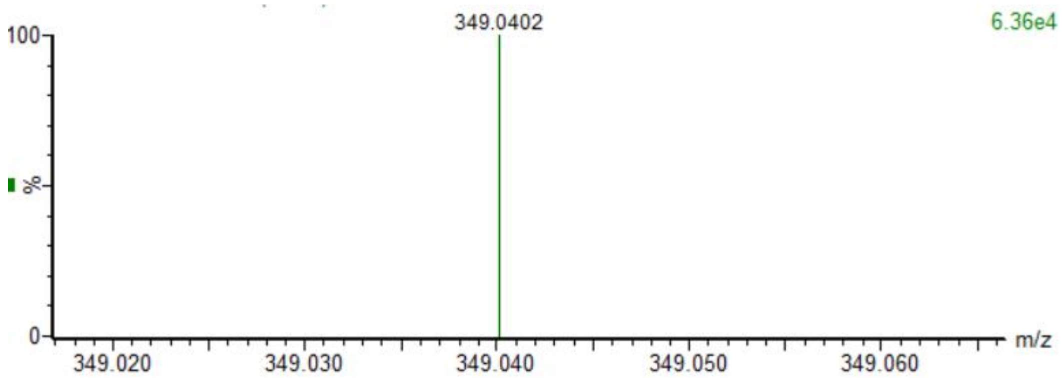

Figure S5. HRMS of **10**

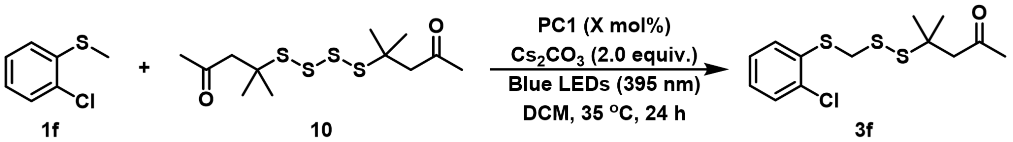

Sulfide **1f** (0.6 mmol, 3.0 equiv.), **10** (0.2 mmol, 1.0 equiv.), PC1 (0-50 mol%), and Cs_2CO_3 (0.4 mmol, 2.0 equiv.), DCM (2.0 mL) were added to a 10 mL reaction tube. The tube was then sealed, and the reaction was irradiated under a 395 nm light source for 24 h, and the temperature of the reaction solution was controlled at 35 °C through a low-temperature cooling circulation pump. After the reaction is complete, the product is detected by GC uing dodecane as an internal standard.

5. Stern-Volmer fluorescence quenching experiments

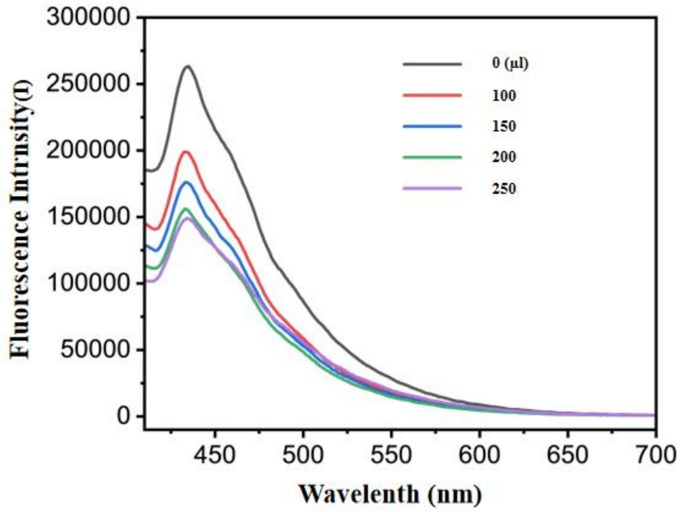

Figure S6. Fluorescence quenching of Cl-BP at variable concentrations of **1f.**

The excitation wavelength was 395 nm. Emission wavelength was 433 nm and the emission spectra were recorded between 420 and 750 nm. The samples were prepared by mixing Cl-BP (0.5×10^{-3} mol/L) and quenchers **1f** in degassed DCM (total volume = 2.0 mL) in a light path quartz fluorescence cuvette. The concentration of **1f** stock solution is 0.5×10^{-3} mol/L in DCM. For each quenching experiment, each volume of quenchers stock solution was titrated to a mixed solution of Cl-BP (0, 100, 150, 200, 250μL, in a total volume = 2.0 mL, ignoring changes in volume). Then the emission intensity was collected, and the results were presented in **Figure S6**. The observations indicate that the fluorescence intensity of Cl-BP significantly decreases along with the increasing of concentration of **1f**, and the Cl-BP is believed to be quenched by **1f**

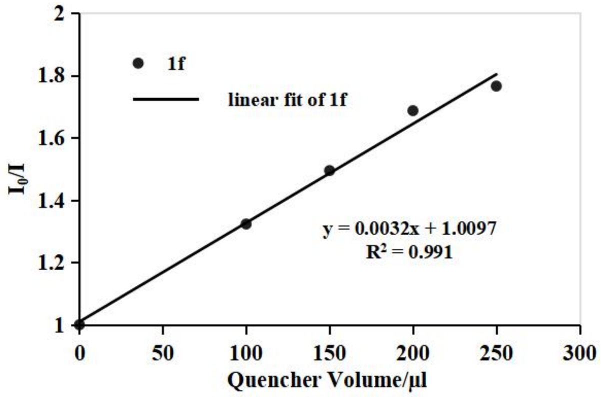

Figure S7. Stern-Volmer plots of Cl-BP with **1f** quenchers.

6. Light control experiments

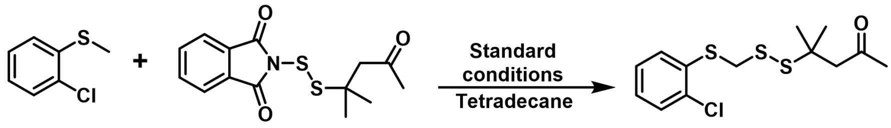

1f **2a** **3f**

Sulfide **1f** (0.6 mmol, 3.0 equiv.), **2** (0.2 mmol, 1.0 equiv.), tetradecane (50 μL.),

PC1 (15 mol%), and Cs_2CO_3 (0.4 mmol, 2.0 equiv.), DCM (2.0 mL) were added to a 10 mL reaction tube. After that, the reaction was placed under a 10 W 395 nm Bule LED, stirred and irradiated, as the time period indicated in **Figure. S8**. At the end of each period, a small portion (50 μL) of the reacting solution using a syringe and analyze it by GC.

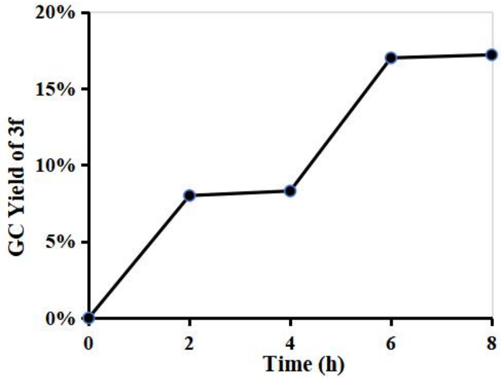

Figure S8. Light on/off experiments over time.

7. Synthesis and characterization of materials 6 and 7

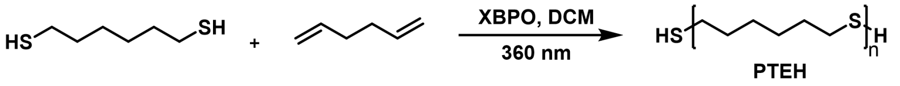

According to the literature[4]: To a solution of dithiol monomer (3.15 mmol) and diene monomer (3.0 mmol) in 3.0 mL DCM were added XBPO (0.025 mmol), and the solution was stirred while being exposed to the UV light for 2 h at a wavelength of 365 nm at room temperature. The solution was then precipitated into MeOH and dried in a vacuum oven at 40 °C to obtain the **PTEH** polymers as white powders.

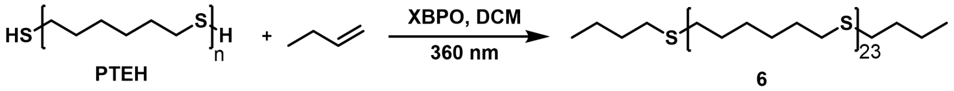

According to the literature[6]: To a solution of PTEH polymer (0.06 mmol) and 1-butylene (0.36 mmol) in 2 mL DCM was added XBPO (0.004 mmol), and the solution was stirred while being exposed to the UV light for 2 h at a wavelength of 365 nm (~100 mW/cm^2) at room temperature. The solution was then precipitated into MeOH and dried in a vacuum oven at 40 °C to obtain the PTEBu polymers (**6**) as white powders. The solutions of polymer in THF were then prepared, followed by the addition of n-hexane dropwise till the solution just turned turbid. The fraction with the largest molecular weight was taken out by centrifugation. Repeated operations on the supernatant were subsequently carried out to obtain fractions with successively smaller molecular weights. With the help of GPC analysis, PTEBu polymers with the number-average molecular weight (Mn) of ~3700 kDa were selected for the following study. White powders, yield: 32%, **^{1}H NMR** (600 MHz, $CDCl_3$): 2.50 (br m, 4H,

SC$\underline{H_2}$CH2 CH2), 1.58 (br m, 4H, SCH2C$\underline{H_2}$CH2); 1.40 (br m, 4H, SCH2CH2C$\underline{H_2}$); 0.92 (br m, terminal C$\underline{H_3}$).

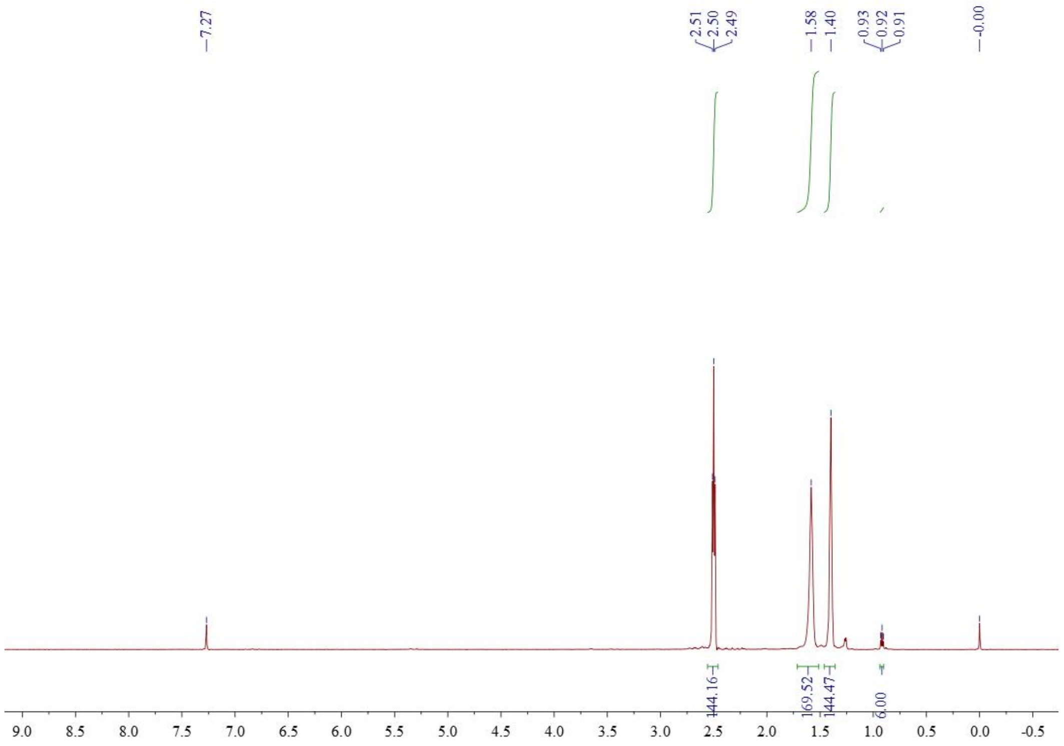

Figure S9. ^{1}**H NMR** spectra of **6.**

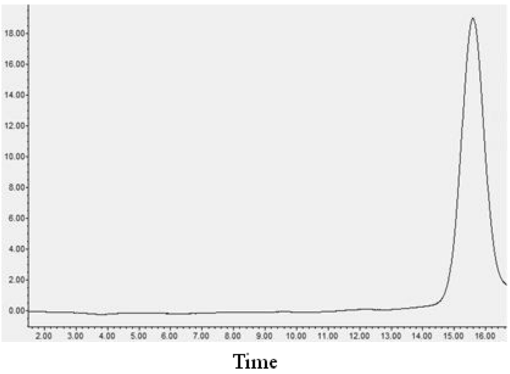

Figure S10. GPC profiles of polymers **6** with selected fraction in THF.

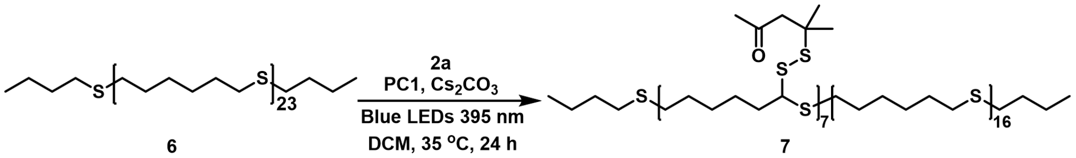

6 7

Sulfide **6** (76 mg), persulfur reagent **2** (61.8 mg), PC1 (7.53 mg), Cs_2CO_3 (130.4 mg), and DCM (5.0 mL) were added to a 10 mL reaction tube. The tube was then sealed, and the reaction was irradiated under a 395 nm Blue LEDs light source for 24 h, and the temperature of the reaction solution was controlled at 35 °C through a low-temperature cooling circulation pump. After the reaction, add water and extract with DCM. Then, dry the organic layer with anhydrous sodium sulfate and remove the solvent under vacuum decompression. The polymer solution was subsequently

prepared in THF, and MeOH (methanol) was added until the solution became turbid. The mixture was then subjected to extraction by centrifugation, which was taken for **^{1}H NMR** (**Figure S11**), **^{13}C NMR** (**Figure S12**), **IR** (**Figure S13**), and GPC (~4000 kDa) (**Figure S14**) analysis. The disulfuration ratios was 30% (7/23).

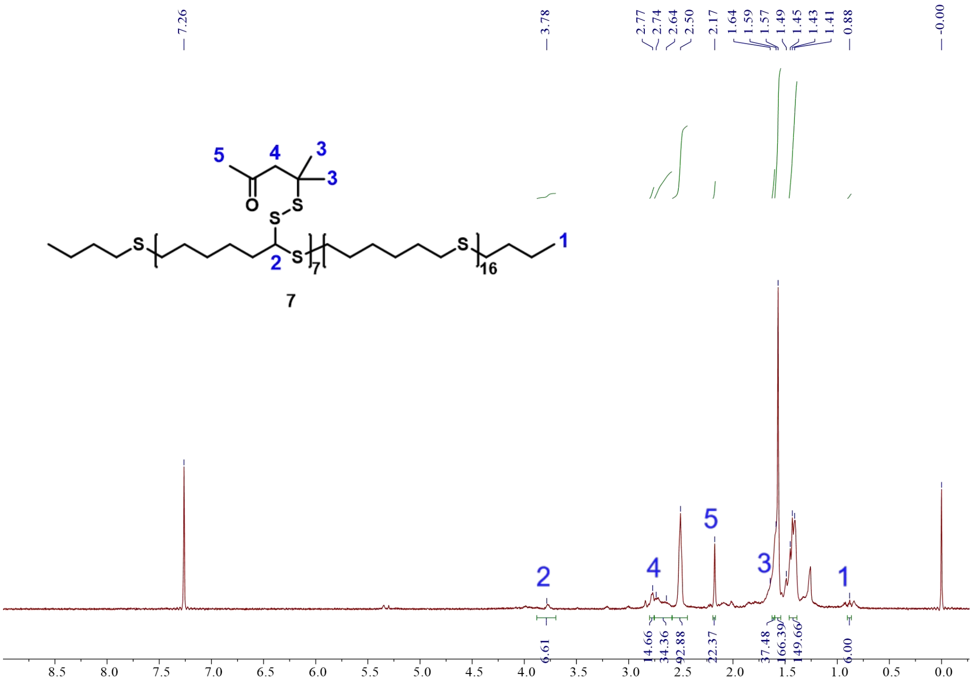

Figure S11. **^{1}H NMR** spectra of **7**.

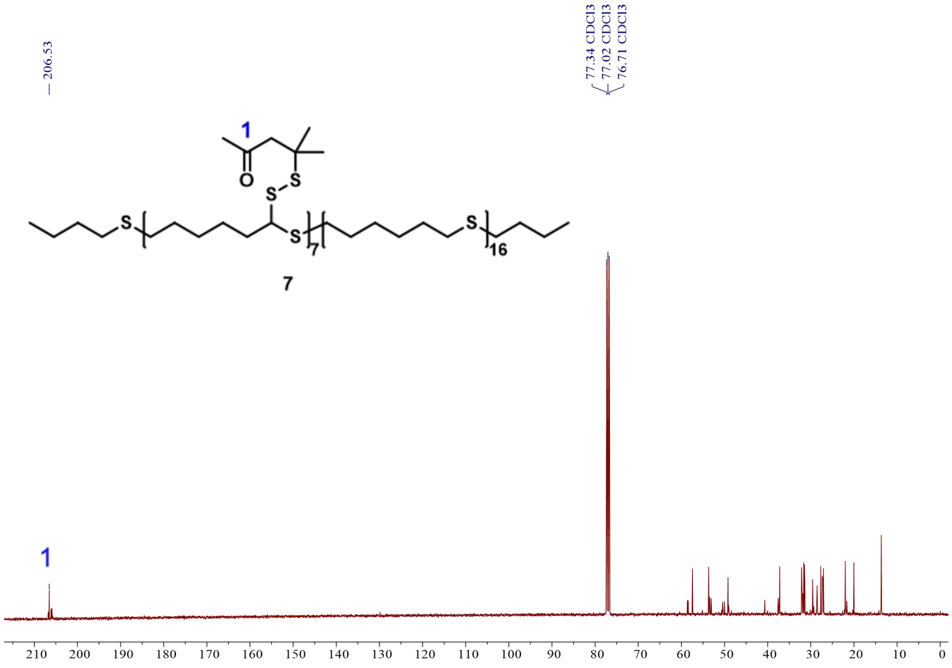

Figure S12. **^{13}C NMR** spectra of **7**.

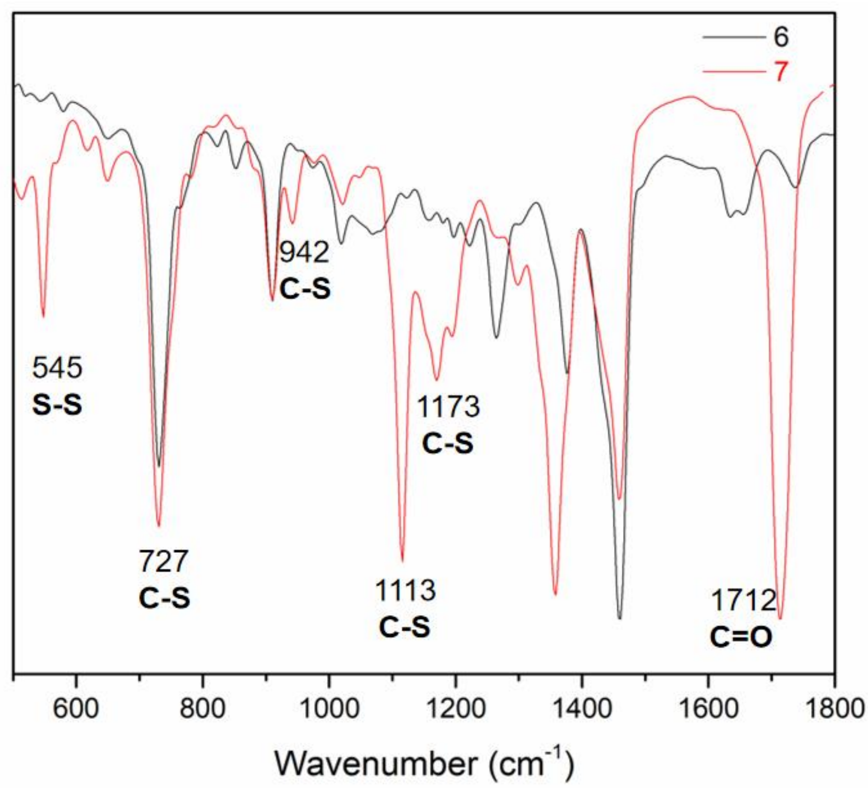

Figure S13. IR spectra of **6** and **7.**

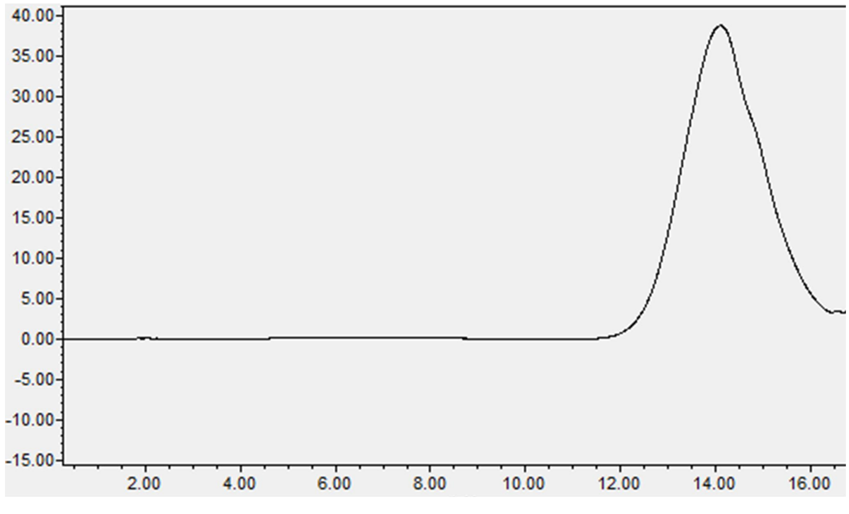

Figure S14. GPC profiles of polymers **7** with selected fraction in THF.

8. Evaluation of bactericidal activity

NB culture medium: Add beef peptone (3.0 g), yeast powder (1.0 g) and glucose (10.0 g) into distilled water (1.0 L) in turn, stir and dissolve them, adjust the pH to 7.2 with NaOH, then separately pack them into test tubes (4.0 mL/tube) with silica plugs, and sterilize them at 121 °C for 20 min.

This medium is used for *Xoo*, *Xoc*, *D.zeae*, and *Xac* bacteria culture and testing.

Blank control: acetone in sterile distilled water served as a blank control,

thiediazole copper served as positive controls.

Positive control: thiediazole copper (20% water dispersible granules) as positive controls.

Test process: In the sterile operating platform, the prepared solution was sucked out with a pipette gun and divided into three equal parts, which were respectively put into the test tube with the medium. Shake well and use the pipette to take 0.2 mL medium from each test tube for the initial $OD_{600\ nm}$ determination, then add the bacterial solution (4.0 µL/tube) and cover with silicone plug. Then put in the shaking table, set the shaking table at 28 ± 1°C, 180 rpm. The $OD_{600\ nm}$ value was measured 9 to 36 h later.

The relevant calculation formula:

Bacteriostatic rate% = [(control absorbance - treatment absorbance)/control absorbance] x 100%

Table S3. Antibacterial activity of partial target compounds against *Xoo* and *Xoc*[a]

Compd.	Inhibition rate (%) against *Xoo*		Inhibition rate (%) against *Xoc*	
	200 mg/L	100 mg/L	200 mg/L	100 mg/L
3a	19.7 ± 1.0	≤10	34.8 ± 3.3	≤10
3b	53.0 ± 0.8	≤10	61.3 ± 1.0	50.7 ± 0.9
3c	≤10	≤10	83.8 ± 4.9	33.8 ± 8.1
3d	≤10	≤10	94.7 ± 4.7	64.2 ± 6.0
3e	34.2 ± 5.5	16.6 ± 2.4	71.2 ± 4.5	37.5 ± 2.4
3f	100	98.8 ± 0.5	100	100
3g	33.3 ± 0.7	≤10	≤10	≤10
3h	≤10	≤10	57.4 ± 7.1	52.7 ± 3.0
3i	39.8 ± 3.4	12.5 ± 2.0	54.0 ± 2.2	36.3 ± 7.6
3j	≤10	≤10	≤10	≤10
3k	≤10	≤10	≤10	≤10
3l	37.4 ± 6.6	≤10	92.1 ± 3.3	25.5 ± 10.0
3m	92.5 ± 0.5	73.7 ± 9.9	-	-
3n	39.9 ± 2.8	≤10	≤10	≤10
3o	54.7 ± 5.3	20.7 ± 5.0	33.9 ± 2.3	33.4 ± 2.7
3p	≤10	≤10	40.9 ± 3.7	31.6 ± 8.3
3q	47.4 ± 4.2	≤10	70.2 ± 4.3	67.0 ± 4.5
3r	31.2 ± 4.0	28.1 ± 3.0	35.0 ± 3.2	26.5 ± 3.3
3s	45.1 ± 4.7	≤10	30.8 ± 7.1	≤10
3t	73.8 ± 1.2	39.7 ± 8.3	76.2 ± 1.8	66.2 ± 2.0
3u	63.3 ± 4.2	≤10	54.7 ± 1.0	29.5 ± 5.6

3v	63.1 ± 9.2	≤10	31.6 ± 1.2	≤10
3w	37.8 ± 3.0	32.3 ± 3.7	60.9 ± 2.3	40.5 ± 1.7
5a	≤10	≤10	87.4 ± 7.4	29.0 ± 0.9
5b	≤10	≤10	85.1 ± 6.7	31.9 ± 6.4
5c	≤10	≤10	≤10	≤10
5e	49.4 ± 4.2	≤10	≤10	≤10
5f	22.2 ± 7.1	13.7 ± 2.4	50.2 ± 4.5	21.5 ± 1.0
5g	≤10	≤10	47.4 ± 3.7	11.8 ± 1.8
5h	≤10	≤10	≤10	≤10
5i	74.8 ± 5.4	≤10	53.6 ± 3.6	42.9 ± 1.4
9	≤10	≤10	41.8 ± 8.8	37.9 ± 5.4
TC[b]	87.4 ± 1.3	62.3 ± 1.3	79.6 ± 0.4	59.4 ± 3.2

[a]*Xoo*, Xanthomonasoryzaepv.oryzae; *Xoc*, Xanthomonas oryzac pathovar oryzicola; TC, thiodiazole copper. [b]TC was used as positive control agents. All results are expressed as mean ± SD, n = 3. -Not tested.

Table S4. Antibacterial activity of partial target compounds against *D.zeae* and *Xac*[a]

Compd	Inhibition rate (%) against *D.zeae*		Inhibition rate (%) against *Xac*	
	200 mg/L	100 mg/L	200 mg/L	100 mg/L
3a	20.2 ± 4.9	≤10	45.5 ± 0.6	≤10
3b	≤10	≤10	25.3 ± 2.0	≤10
3c	≤10	≤10	35.8 ± 7.4	≤10
3d	≤10	≤10	66.5 ± 3.2	32.2 ± 2.5
3e	23.6 ± 0.2	14.1 ± 0.3	51.9 ± 5.4	48.5 ± 3.7
3f	98.8 ± 0.5	96.0 ± 5.3	98.9 ± 0.1	97.3 ± 0.5
3g	29.6 ± 1.0	18.6 ± 1.2	51.9 ± 5.4	≤10
3h	53.7 ± 4.0	39.0 ± 0.8	34.4 ± 2.0	25.0 ± 2.0
3i	17.6 ± 0.8	≤10	≤10	≤10
3j	55.6 ± 2.2	30.6 ± 1.6	≤10	≤10
3k	≤10	≤10	≤10	≤10
3l	39.5 ± 0.4	25.5 ± 4.0	52.4 ± 1.7	43.1 ± 2.6
3m	87.3 ± 0.9	78.6 ± 3.0	-	-
3n	32.6 ± 6.0	29.1 ± 9.8	81.3 ± 0.8	79.0 ± 1.1
3o	54.7 ± 5.3	≤10	20.5 ± 1.3	13.5 ± 3.2
3p	≤10	≤10	21.4 ± 7.0	17.4 ± 4.0
3q	80.9 ± 0.9	≤10	56.9±2.9	32.2 ± 3.5
3r	21.4 ± 4.0	16.6 ± 2.3	≤10	≤10
3s	≤10	≤10	≤10	≤10
3t	73.8 ± 1.2	26.4 ± 2.2	99.3 ± 0.1	35.8 ± 4.7
3u	63.6 ± 4.2	42.9 ± 4.1	38.8 ± 4.1	26.3 ± 3.5

3v	70.8 ± 7.2	51.9 ± 9.8	56.5 ± 3.8	≤10
3w	63.4 ± 2.3	39.0 ± 1.4	50.1 ± 7.3	27.6 ± 8.4
5a	77.3 ± 0.1	47.6 ± 0.3	≤10	≤10
5b	16.4 ± 8.2	≤10	92.7 ± 7.4	65.6 ± 2.9
5c	50.5 ± 3.0	36.7 ± 2.7	39.7 ± 6.4	≤10
5e	49.4 ± 4.2	≤10	≤10	≤10
5g	≤10	≤10	≤10	≤10
5h	≤10	≤10	28.8 ± 4.1	≤10
5i	67.2 ± 5.4	≤10	41.7 ± 4.6	29.4 ± 4.7
9	≤10	≤10	99.6 ± 0.1	78.0 ± 2.8
TC[b]	100	95.3 ± 1.0	90.2 ± 2.6	72.1 ± 3.0

[a]*D.zece*, Dickeya zeae; *Xac*, Xanthomonas citrisubsp citri; TC, thiodiazole copper. [b]TC was used as positive control agents. All results are expressed as mean ± SD, n = 3. -Not tested.

Table S5. EC_{50} values of some target compounds against *Xoo*, *Xoc*, *D.zeae*, and *Xac*[a]

Compd.	Bacteria	Toxic regression equation	R	EC_{50} (mg/L)	CI (mg/L) [b]
3f		y = 3.7528 x – 1.2713	0.99	46.89	42.82 - 51.35
3m	*Xoo*	y = 2.5072 x + 0.4376	0.98	66.02	63.25 - 68.92
PC[c]		y = 1.2807 x + 3.2703	0.95	64.80	62.34 - 68.60
3d		y = 4.5185 x – 3.1477	0.97	63.56	56.75 - 71.19
3f	*Xoc*	y = 2.8826 x + 0.6640	0.97	31.93	31.43 - 32.43
PC[c]		y = 3.0259 x – 0.5341	0.96	67.44	64.20 - 70.60
3f		y = 2.5241 x + 0.9506	0.96	40.21	37.02 - 43.60
3m	*D.zeae*	y = 3.0106 x – 0.2634	0.99	56.07	51.95 - 60.39
PC[c]		y = 1.7201 x + 2.6209	0.95	24.30	23.91 - 24.83
3f		y = 0.0106 x – 0.0316	0.98	38.56	36.61 - 40.60
5i	*Xac*	y = 3.0244 x + 0.0235	0.95	44.21	42.98 - 46.43
PC[c]		y = 1.4940 x + 2.3012	0.98	65.10	63.12 - 67.21

[a] Average of three replicates; Dickeya zeae, *D.zeae*; Xanthomonas axonopodis pv. citri, *Xac*; [b] CI, 95% Confidence interval (mg/L); [c] Thiodiazole-copper, PC.

9. X-Ray crystallographic data

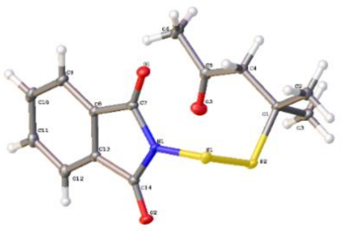

Figure S15. X-Ray of **2a** (CCDC 2389271)

X-Ray crystal structure of disulfide transfer reagent **2a** (**CCDC 2389271**). The crystal was grown from petroleum ether. 10.0 mg of **2a** was dissolved in ethyl acetate (2.0 mL) and the solvent was evaporated slowly in a room atmosphere.

Table S6. Crystal data and structure refinement for **2a**

Empirical formula	$C_{14}H_{15}NO_3S_2$
Formula weight	309.39
Temperature/K	170.00 K
Wavelength	1.34139 Å
Crystal system	Monoclinic
Space group	P 1 21/c 1
Unit cell dimensions	a = 8.7116 (2) Å, α = 90°
	b = 9.5478 (2) Å, β = 93.3080°
	c = 17.2742 (3) Å, γ = 90°
Volume	1434.42 (5) $Å^3$
Z	4
Density (calculated)	1.433 Mg/m^3
Absorption coefficient	2.255 mm^{-1}
F (000)	648
Crystal size	0.17 x 0.17 x 0.05 mm^3
Theta range for data collection	4.461 to 54.934°
Index ranges	-10<=h<=10, -10<=k<=11, -20<=l<=21
Reflections collected	15192
Independent reflections	2708 [R(int) = 0.0585]
Completeness to theta = 53.594°	99.2 %
Absorption correction	Semi-empirical from equivalents
Max. and min. transmission	0.7508 and 0.5380
Refinement method	Full-matrix least-squares on F^2
Data / restraints / parameters	2708 / 0 / 184
Goodness-of-fit on F^2	1.048
Final R indices [I>2sigma(I)]	R1 = 0.0336, wR2 = 0.0848
R indices (all data)	R1 = 0.0401, wR2 = 0.0899
Extinction coefficient	n/a
Largest diff. peak and hole	0.365 and -0.409 e.$Å^{-3}$

10. DFT calculation

1. HOMO and LUMO calculation of 3f and 3c

The most effective chemical **3f** and the less active chemical **3c**, were selected for DFT analysis in Gaussian 09W software. The DFT–B3LYP/6-31G method was employed to optimize the two structures. After a vibrational analysis was conducted, it was determined that the optimized structures did not contain any false frequencies and were therefore deemed stable.

Table S7. Frontier orbital total energy, energy difference between HOMO and LUMO (ΔE), Clog P, and topological polar surface areas (TPSA) of compounds **3c** and **3f**

	**3c**	**3f**
E_{total}/hartee	-1776.05	-2235.63
E_{HOMO}/hartee	-0.2296	-0.2308
E_{LUMO}/hartee	-0.0276	-0.0364
△E/hartee	0.2020	0.1944
Clog P	4.08	4.84
TPSA	17.07	17.07

Table S8. Calculate coordinates of **3c**

Entry	Atom	X	Y	Z
1	C	4.696592	2.254654	-1.347239
2	C	4.487848	0.980553	-1.877110
3	C	3.868398	-0.006342	-1.108950
4	C	3.453406	0.282993	0.196457
5	C	3.673718	1.559240	0.730938
6	C	4.289222	2.542950	-0.043221
7	S	2.701303	-0.995969	1.213681
8	C	0.920389	-0.652363	0.989112
9	S	0.318360	-1.365620	-0.614379
10	S	-1.561055	-0.507064	-0.858163
11	C	-2.813127	-1.743208	-0.142899
12	C	-4.135525	-0.974954	-0.345970
13	C	-5.427116	-1.683658	0.068208
14	C	-2.772720	-3.039849	-0.958442
15	C	-2.522398	-2.005911	1.337614
16	O	-5.455969	-2.756352	0.641012
17	C	-6.708178	-0.947794	-0.289778
18	H	5.179171	3.020478	-1.947303
19	H	4.804797	0.752765	-2.890492

20	H	3.700809	-0.997400	-1.516199
21	H	3.368032	1.773815	1.750202
22	H	4.456338	3.531352	0.374720
23	H	0.409226	-1.126157	1.828686
24	H	0.729998	0.421666	1.001749
25	H	-4.107544	-0.025439	0.208247
26	H	-4.247494	-0.688313	-1.401193
27	H	-3.505196	-3.747722	-0.561592
28	H	-1.781195	-3.497346	-0.898091
29	H	-2.996947	-2.851455	-2.012735
30	H	-1.545229	-2.483840	1.453647
31	H	-2.528147	-1.076850	1.916320
32	H	-3.281139	-2.678779	1.744459
33	H	-6.838948	-0.938729	-1.378415
34	H	-7.559356	-1.451827	0.169391
35	H	-6.670423	0.097178	0.036031

Table S9. Calculate coordinates of **3f**

Entry	Atom	X	Y	Z
1	C	1.623400	-1.639358	-1.639358
2	C	5.136992	0.230245	-1.641904
3	C	4.368260	-0.432948	-0.687974
4	C	3.649636	0.273317	0.288190
5	C	3.730113	1.677292	0.279518
6	C	4.487065	2.348971	-0.680733
7	S	2.768895	-0.661132	1.538081
8	C	1.019883	-0.366153	1.101381
9	S	0.536264	-1.387100	-0.368727
10	S	-1.273919	-0.554532	-0.964816
11	C	-2.637828	-1.574868	-0.124490
12	C	-3.900849	-0.831290	-0.606389
13	C	-2.588576	-3.016402	-0.641744
14	C	-2.486266	-1.522583	1.398554
15	C	-5.254556	-1.398859	-0.173251
16	C	-6.465194	-0.716163	-0.788766
17	O	-5.381332	-2.326754	0.602969
18	Cl	2.882349	2.648797	1.473614

19	H	5.783110	2.152119	-2.380935
20	H	5.681964	-0.339988	-2.387362
21	H	4.308272	-1.515734	-0.688281
22	H	4.523566	3.432427	-0.665659
23	H	0.445355	-0.665295	1.979756
24	H	0.839198	0.688395	0.892584
25	H	-3.874980	0.213769	-0.264972
26	H	-3.910165	-0.774750	-1.704103
27	H	-3.384161	-3.602777	-0.174581
28	H	-1.628071	-3.477458	-0.394811
29	H	-2.713640	-3.051587	-1.728213
30	H	-1.547286	-1.993986	1.703256
31	H	-2.494884	-0.491455	1.765526
32	H	-3.308799	-2.069656	1.865580
33	H	-6.407162	0.371729	-0.676062
34	H	-6.502303	-0.924583	-1.864611
35	H	-7.374619	-1.092209	-0.318765

2. DFT calculation of reaction pathway

Modeling and calculation parameter setup were performed using Gauss View 5.0 software, and structural optimization calculations were conducted with Gaussian 09W software. The calculation method was set as the m062x functional combined with the def2tzvp basis set. The IEFPCM solvation model was applied with dichloromethane as the solvent.

Specific steps:

1) Build structural models using Gauss View 5.0 software and configure the above-mentioned calculation method;

2) Use Gaussian 09W software to perform structural optimization and transition state calculations for all structures along two reaction pathways;

3) Based on the calculated energy barrier data.

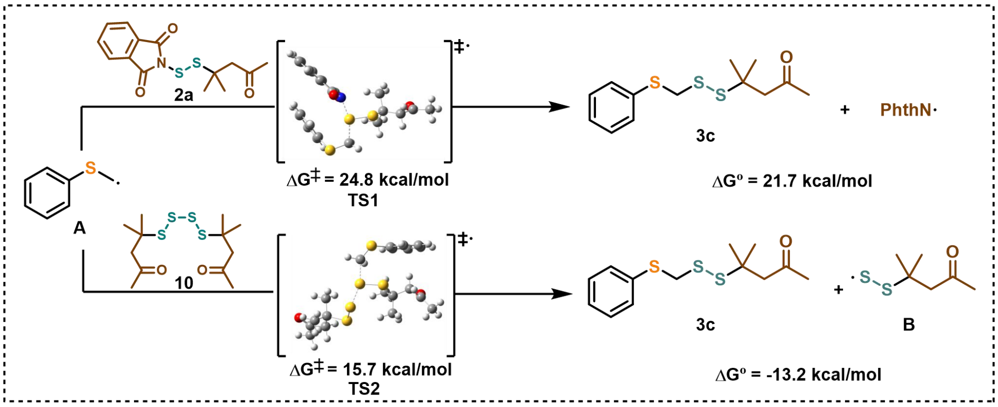

Figure S16. DFT calculation

Table S10. Calculate coordinates of **A**

Entry	Atom	X	Y	Z
1	C	-2.09497600	1.01637100	0.12727700
2	C	-0.73172400	1.25943600	0.07629000
3	C	0.15979300	0.19622300	-0.06361000
4	C	-0.32115800	-1.10656600	-0.15397400
5	C	-1.68839000	-1.34022400	-0.08817800
6	C	-2.57866000	-0.28460200	0.05138300
7	H	-2.78146300	1.84613100	0.23546100
8	H	-0.35376500	2.27179100	0.15233500
9	H	0.36439600	-1.93175400	-0.29241300
10	H	-2.05717700	-2.35567200	-0.15663500
11	H	-3.64305200	-0.47288600	0.09847800
12	S	1.87685200	0.59804800	-0.16112700
13	C	2.67114300	-0.83075100	0.29190600
14	H	3.73712400	-0.84948100	0.12866700
15	H	2.20813200	-1.53621300	0.96558000

Table S11. Calculate coordinates of **2a**

Entry	Atom	X	Y	Z
1	C	-2.99506000	0.69917000	0.47156500
2	C	-3.22490100	-0.20440900	-0.55379200
3	C	-4.30896600	-0.08759100	-1.39800700
4	C	-5.17146100	0.98300900	-1.17739100
5	C	-4.94249600	1.89191800	-0.14623600
6	C	-3.84375200	1.76311700	0.70035800
7	C	-1.75497000	0.29650300	1.18993000
8	C	-2.14821200	-1.22780200	-0.53498700
9	H	-4.47827400	-0.79975300	-2.19447600
10	H	-6.03522500	1.11345000	-1.81578800
11	H	-5.63326800	2.71195700	-0.00188500
12	H	-3.66072400	2.46575000	1.50233300
13	O	-1.98923300	-2.17617700	-1.24861400
14	O	-1.23401500	0.80868700	2.14021400
15	S	0.05782100	-1.77824000	0.99400500
16	S	1.43533000	-1.46992300	-0.45325200
17	C	2.46997700	-0.03480400	0.10323400
18	C	1.60939200	1.20999700	0.24639000
19	C	3.16441200	-0.37132600	1.41526600
20	C	3.47442900	0.08442700	-1.06422000
21	H	1.07783400	1.43069300	-0.68047400
22	H	0.88592400	1.07275400	1.05003600
23	H	2.23583200	2.06689200	0.49619600
24	H	3.80219200	-1.25070500	1.31521100
25	H	3.77555900	0.47499300	1.74008100

26	H	2.42606600	-0.56129000	2.19518600
27	H	3.95454900	-0.88193600	-1.23377300
28	H	2.93963600	0.37785200	-1.96974800
29	N	-1.29151900	-0.86052000	0.52724900
30	C	4.53824200	1.13626100	-0.79216200
31	O	4.28756700	2.31317900	-0.90126100
32	C	5.90475400	0.64374600	-0.40715600
33	H	6.53925700	1.47444500	-0.11006200
34	H	5.83515500	-0.09349900	0.39400100
35	H	6.34282000	0.13383500	-1.26909300

Table S12. Calculate coordinates of **TS1**

Entry	Atom	X	Y	Z
1	C	-2.61671100	-2.02403700	-0.81266700
2	C	-2.36374000	-1.85275600	0.53806800
3	C	-3.22343300	-2.33116400	1.50441400
4	C	-4.36645900	-2.99637500	1.06397200
5	C	-4.62244100	-3.16777400	-0.29514100
6	C	-3.74350400	-2.68128300	-1.26167700
7	C	-1.50693700	-1.36570900	-1.56902400
8	C	-1.09554000	-1.07211900	0.66673700
9	H	-3.02207000	-2.18874100	2.55814600
10	H	-5.07067300	-3.38587500	1.78739700
11	H	-5.52061000	-3.68706000	-0.60260800
12	H	-3.93848100	-2.80925200	-2.31834500
13	O	-0.58806700	-0.65785900	1.68076900
14	O	-1.40250800	-1.24292000	-2.76473900
15	S	0.44732700	0.72369400	-1.01146000
16	S	2.27330600	-0.01017700	-1.44800100
17	C	3.14815800	-0.38923500	0.15240300
18	C	2.96267100	0.74189600	1.15073100
19	C	2.61616200	-1.70844600	0.69930600
20	C	4.59702700	-0.51122300	-0.33100400
21	H	3.31526000	1.69123100	0.74429400
22	H	1.90968100	0.83257200	1.42313300
23	H	3.52855000	0.51312500	2.05260200
24	H	2.75739600	-2.51515900	-0.02146300
25	H	3.14470800	-1.95907100	1.61918300
26	H	1.55424700	-1.61713400	0.92487800
27	H	4.66583100	-1.21351200	-1.16937000
28	H	4.94866600	0.45030700	-0.72196200
29	N	-0.59527900	-0.89842800	-0.62235900
30	C	5.62843000	-0.95061600	0.69336900
31	O	5.40122300	-1.02668500	1.87739300

32	C	6.97885800	-1.28999500	0.12192700
33	H	6.90082300	-2.24171800	-0.40900600
34	H	7.29277700	-0.53948200	-0.60406100
35	H	7.71184900	-1.38018400	0.91917900
36	C	-2.56041800	2.44864600	2.28372000
37	C	-1.69192300	3.09334300	1.41468000
38	C	-1.74958300	2.81675100	0.05214500
39	C	-2.68167300	1.90588000	-0.44062700
40	C	-3.55319800	1.27013600	0.43487700
41	C	-3.48970900	1.53747300	1.79591900
42	H	-2.51337200	2.66157200	3.34368200
43	H	-0.97258300	3.81028100	1.79081900
44	H	-2.71783500	1.69502000	-1.50279000
45	H	-4.27474700	0.55901700	0.05145400
46	H	-4.16556700	1.03676600	2.47727500
47	S	-0.62376700	3.59502600	-1.08752600
48	C	0.84138600	2.74244600	-0.64424700
49	H	1.08368400	2.76945300	0.41207500
50	H	1.66985300	2.99482700	-1.3008890

Table S13. Calculate coordinates of **PhthN·**

Entry	Atom	X	Y	Z
1	C	0.01227400	0.14500300	0.69457300
2	C	0.01227400	0.14500300	-0.69457300
3	C	0.00766900	1.32258100	-1.42163500
4	C	-0.00045500	2.50832800	-0.69989700
5	C	-0.00045500	2.50832800	0.69989700
6	C	0.00766900	1.32258100	1.42163500
7	C	0.01201500	-1.26760500	1.14752700
8	C	0.01201500	-1.26760500	-1.14752700
9	H	0.00781500	1.31527300	-2.50310100
10	H	-0.00827600	3.45388900	-1.22534500
11	H	-0.00827600	3.45388900	1.22534500
12	H	0.00781500	1.31527300	2.50310100
13	O	-0.11159000	-1.70799200	-2.25703000
14	O	-0.11159000	-1.70799200	2.25703000
15	N	0.20118900	-2.10144500	0.00000000

Table S14. Calculate coordinates of **3c**

Entry	Atom	X	Y	Z
1	C	4.71655500	1.98395700	0.81596800
2	C	3.84099500	0.93553700	1.05978900
3	C	3.77231000	-0.13280800	0.16937200

4	C	4.58802900	-0.15161100	-0.95796400
5	C	5.46817800	0.89614100	-1.19386600
6	C	5.53203500	1.96410500	-0.30915400
7	H	4.76723800	2.81339600	1.50937700
8	H	3.21242000	0.93886000	1.94153100
9	H	4.53117900	-0.98666600	-1.64423500
10	H	6.10242700	0.87718200	-2.07064400
11	H	6.21872800	2.77991200	-0.49415000
12	S	2.66893300	-1.49382200	0.47898800
13	C	1.14027800	-0.76494300	-0.18283700
14	H	0.99549200	0.22514500	0.24366800
15	H	1.17908300	-0.71454900	-1.26871400
16	S	-0.20914200	-1.88187900	0.31070000
17	S	-1.72988200	-1.29028400	-0.89829500
18	C	-2.68936000	-0.05165100	0.09193900
19	C	-3.35309700	-0.74651600	1.27423100
20	C	-1.78893500	1.07756900	0.56787200
21	C	-3.72686000	0.43838600	-0.94158100
22	H	-4.02445300	-1.53987800	0.94326400
23	H	-2.59606000	-1.18244200	1.92718200
24	H	-3.92341100	-0.02331200	1.86393800
25	H	-1.27899800	1.55611100	-0.26920100
26	H	-2.38259800	1.83078000	1.08685800
27	H	-1.04635800	0.69113600	1.26750900
28	H	-3.21608700	1.01028600	-1.71832300
29	H	-4.21815800	-0.42188100	-1.40237400
30	C	-4.77524100	1.33825700	-0.30823900
31	O	-4.52221100	2.48851300	-0.03607400
32	C	-6.13038600	0.73894300	-0.05794000
33	H	-6.03618300	-0.22841000	0.43730700
34	H	-6.74407900	1.41468200	0.53176400
35	H	-6.60803100	0.55510900	-1.02400100

Table S15. Calculate coordinates of **10**

Entry	Atom	X	Y	Z
1	C	4.97210000	1.50894600	0.35143400
2	O	5.49081800	0.81215300	1.18981500
3	C	5.60790300	2.79140300	-0.11742900
4	H	6.39725500	3.09041300	0.56703000
5	H	6.03540400	2.62185700	-1.10873800
6	H	4.86504500	3.58298400	-0.21660800
7	C	3.65102200	1.17351500	-0.31757400
8	H	2.94325600	1.94835300	0.00021700

9	H	3.79334300	1.32688900	-1.39320400
10	C	3.06897900	-0.21760200	-0.04684300
11	C	4.00897000	-1.32526800	-0.50249600
12	C	2.65861200	-0.39591400	1.40779400
13	H	4.32358400	-1.17048700	-1.53557900
14	H	4.89026600	-1.34211600	0.13843000
15	H	3.51442300	-2.29466900	-0.43028900
16	H	1.95556400	0.37890500	1.71806300
17	H	2.18873200	-1.37011900	1.55190200
18	H	3.54360300	-0.35022600	2.04083500
19	S	1.55701400	-0.18964000	-1.13173400
20	S	0.66410700	-1.98796500	-0.84066800
21	S	-0.59663900	-1.84183400	0.80913800
22	S	-2.50022100	-1.70850400	0.11248400
23	C	-2.91440200	0.09709000	0.12580400
24	C	-2.83292100	0.63538200	1.54852900
25	C	-1.98081600	0.86162700	-0.79809600
26	C	-4.36800100	0.08791900	-0.39697400
27	H	-3.52034700	0.10953100	2.21256200
28	H	-1.82018300	0.52577700	1.93816600
29	H	-3.07630700	1.70142400	1.55870600
30	H	-1.99453500	0.44497700	-1.80634300
31	H	-2.28512100	1.90765600	-0.84957400
32	H	-0.96094900	0.81857600	-0.41579900
33	H	-4.37292900	-0.22379200	-1.44321300
34	H	-4.96294700	-0.61865200	0.18589200
35	C	-5.00397800	1.46675000	-0.31520200
36	O	-4.71970100	2.33035600	-1.11117800
37	C	-6.00603900	1.69097800	0.78199300
38	H	-5.60324700	1.36018600	1.74048400
39	H	-6.29530000	2.73757200	0.82720900
40	H	-6.88428600	1.07301700	0.57759400

Table S16. Calculate coordinates of **TS2**

Entry	Atom	X	Y	Z
1	C	-6.04443000	0.12034200	1.65253800
2	O	-6.45632000	-1.00318600	1.48392500
3	C	-6.61067800	1.01827100	2.72086700
4	H	-7.49305400	0.56311500	3.16254600
5	H	-5.85316600	1.17194300	3.49232100
6	H	-6.85463000	1.99768800	2.30836900
7	C	-4.92772300	0.73574100	0.82974000
8	H	-5.35875100	1.61413700	0.33575100

9	H	-4.19296600	1.12899700	1.54091900
10	C	-4.24258700	-0.16610900	-0.20043000
11	C	-3.54367400	-1.34998400	0.45369200
12	C	-5.19591600	-0.61583000	-1.29906400
13	H	-2.83989100	-1.01832700	1.21930400
14	H	-4.28619300	-2.00398900	0.91012100
15	H	-2.99672600	-1.92670500	-0.29414700
16	H	-5.72220300	0.23671400	-1.73071200
17	H	-4.64219800	-1.12076700	-2.09165200
18	H	-5.92635400	-1.31216400	-0.88920600
19	S	-2.97263200	0.99100200	-0.91654900
20	S	-1.95112800	-0.09030800	-2.27188600
21	S	-0.28139400	-1.10994200	-1.08592100
22	S	1.35871100	-0.02374000	-1.54229300
23	C	1.42635400	1.42545800	-0.37528900
24	C	0.62294100	2.57792300	-0.96749400
25	C	0.90836500	1.06618200	1.00569200
26	C	2.94121900	1.72507300	-0.35478000
27	H	0.99004200	2.85232500	-1.95749400
28	H	-0.42675500	2.29721100	-1.05323900
29	H	0.68279600	3.45351700	-0.31463400
30	H	1.49779600	0.26445800	1.45070100
31	H	0.97884500	1.93940400	1.65508800
32	H	-0.13743000	0.76025800	0.95497100
33	H	3.45545800	0.89767700	0.13853500
34	H	3.31206900	1.80994600	-1.37971000
35	C	3.26798200	2.99839200	0.40524600
36	O	3.28476900	3.00852300	1.61545600
37	C	3.58860300	4.22170800	-0.40528100
38	H	4.53665200	4.05202400	-0.92209900
39	H	2.83321300	4.37780600	-1.17655900
40	H	3.67014300	5.09610600	0.23484800
41	C	4.45190800	-0.78489500	2.27117300
42	C	3.43848900	-1.60439500	1.79261000
43	C	3.48868700	-2.07168600	0.48240600
44	C	4.56823500	-1.73969700	-0.33389400
45	C	5.58909600	-0.94046500	0.15976000
46	C	5.53017900	-0.45360000	1.46000900
47	H	4.40208500	-0.41584600	3.28732700
48	H	2.62312800	-1.88724200	2.44589800
49	H	4.59972200	-2.09865500	-1.35491400
50	H	6.42400300	-0.68564800	-0.47979900
51	H	6.32042600	0.18028200	1.83980900
52	S	2.27298300	-3.16917500	-0.18556000

53	C	0.77225000	-2.58729100	0.40922200
54	H	0.78171000	-1.94254300	1.27805000
55	H	-0.00580200	-3.34062100	0.38958000

Table S17. Calculate coordinates of **B**

Entry	Atom	X	Y	Z
1	S	-3.11218700	0.05808600	0.26345800
2	S	-1.55535300	0.40473900	-0.84474600
3	C	-0.05306000	-0.15481900	0.09883400
4	C	0.02918300	0.61601900	1.40891600
5	C	-0.15344900	-1.65290800	0.34498000
6	C	1.09543100	0.20967200	-0.86247300
7	H	0.10764700	1.69088600	1.23771000
8	H	-0.85582700	0.42162500	2.01507000
9	H	0.90260900	0.28508100	1.97647100
10	H	-0.20944200	-2.20408400	-0.59444200
11	H	0.72507600	-1.99482300	0.89248600
12	H	-1.04078700	-1.87370900	0.93917300
13	H	1.01883900	-0.39669900	-1.76743000
14	H	1.02194200	1.26410200	-1.13762000
15	C	2.45667200	-0.05844400	-0.23858600
16	O	2.88184300	-1.18531700	-0.14395500
17	C	3.23939900	1.13438200	0.23186400
18	H	2.61957800	1.77088800	0.86552600
19	H	4.13286300	0.81892000	0.76396600
20	H	3.51833700	1.73175000	-0.63986900

11.Characteristic data of compound

2-((2-Methyl-4-oxopentan-2-yl)disulfaneyl)isoindoline-1,3-dione (2a)[2]

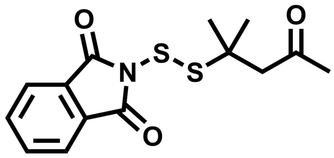

White solid, **Rf** = 0.3 (PE : EA = 6 : 1). **^{1}H NMR** (600 MHz, $CDCl_3$) δ 7.89 (d, *J* = 9.6 Hz, 2H), 7.77 (d, *J* = 9.0 Hz, 2H), 2.92 (s, 2H), 2.09 (s, 3H), 1.45 (s, 6H). **^{13}C NMR** (151 MHz, $CDCl_3$) δ 205.92, 167.46, 134.77, 132.03, 123.95, 52.87, 50.13, 31.74, 27.20. **HRMS (ESI)** calcd for $[M + Na]^+$ ($C_{14}H_{15}NaNO_3S_2$): 332.0391; found: 332.0396.

***SS*-(2-Methyl-4-oxopentan-2-yl) benzenesulfono(dithioperoxoate) (2b)**[2]

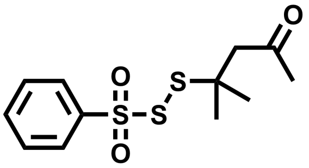

Colorless oil, yield 71%, **Rf** = 0.4 (PE : EA = 5 : 1). **^{1}H NMR** (600 MHz, $CDCl_3$) δ 7.90 (d, *J* = 7.5 Hz, 2H), 7.65 (t, *J* = 7.4 Hz, 1H), 7.56 (t, *J* = 7.7 Hz, 2H), 2.90 (s, 2H), 2.17 (s, 3H), 1.48 (s, 6H). **^{13}C NMR** (151 MHz, $CDCl_3$) δ 205.60, 142.81, 134.06, 129.20, 127.88, 53.55, 50.63, 31.22, 27.67. **HRMS** (ESI) calcd for $[M + Na]^+$ ($C_{12}H_{16}NaO_3S_3$): 327.0159, found 327.0150.

4-Methyl-4-((phenylamino)disulfaneyl)pentan-2-one (2c)

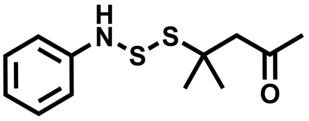

Colorless oil, yield 79%, **Rf** = 0.2 (PE : EA = 5 : 1). **^{1}H NMR** (600 MHz, $CDCl_3$) δ 7.26 (t, *J* = 7.8 Hz, 2H), 7.04 (d, *J* = 8.4 Hz, 2H), 6.94 (td, *J* = 7.3, 0.6 Hz, 1H), 5.18 (s, 1H), 2.74 (s, 2H), 2.08 (s, 3H), 1.46 (s, 6H). **^{13}C NMR** (151 MHz, $CDCl_3$) δ 206.94, 145.75, 129.30, 121.73, 117.12, 54.59, 49.54, 32.14, 28.34. **HRMS (ESI)** calcd for $[M + Na]^+$ ($C_{12}H_{17}NaNOS_2$): 278.0649; found: 278.0656.

4-((Diethylamino)disulfaneyl)-4-methylpentan-2-one (2d)

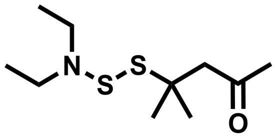

Colorless oil, yield 72%, **Rf** = 0.4 (PE : EA = 5 : 1). **^{1}H NMR** (600 MHz, $CDCl_3$) δ 2.84 (q, *J* = 7.0 Hz, 4H), 2.76 (s, 2H), 2.16 (s, 3H), 1.46 (s, 6H), 1.17 (t, *J* = 7.1 Hz, 6H). **^{13}C NMR** (151 MHz, $CDCl_3$) δ 206.81, 54.87, 51.50, 46.58, 32.27, 28.81, 13.75. **GC-MS** (EI, 70 ev): m/z(%) = 235 (M^+, 10), 137 (50), 72 (100), 99 (20), 58 (30). **HRMS (ESI)** calcd for $[M + Na]^+$ ($C_{10}H_{21}NaNOS_2$): 258.0962; found: 258.0949.

4-Methyl-4-(morpholinodisulfaneyl)pentan-2-one (2e)

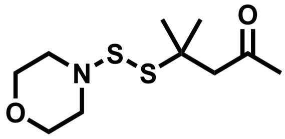

Colorless oil, yield 80%, **Rf** = 0.5 (PE : EA = 5 : 1). **^{1}H NMR** (600 MHz, $CDCl_3$) δ 3.70 – 3.60 (m, 4H), 2.87 – 2.80 (m, 4H), 2.72 (s, 2H), 2.13 (s, 3H), 1.45 (s, 6H). **^{13}C NMR** (151 MHz, $CDCl_3$) δ 206.31, 67.12, 55.63, 54.68, 47.01, 32.12, 28.55. **GC-MS** (EI, 70 ev): m/z(%) = 249 (M^+, 10), 151 (30), 99 (30), 87 (30), 86 (100), 56 (20).

HRMS (ESI) calcd for $[M + Na]^+$ ($C_{10}H_{19}NaNO_2S_2$): 272.0755; found: 272.0753.

4,4'-Tetrasulfanediylbis(4-methylpentan-2-one) (10)[4]

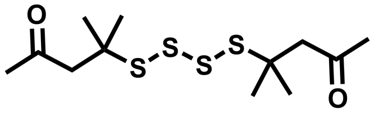

Colorless oil, yield: 60%, **Rf** = 0.3 (PE : EA = 10 : 1). **^{1}H NMR** (600 MHz, $CDCl_3$) δ 2.81 (s, 4H), 2.15 (s, 6H), 1.45 (s, 12H). **^{13}C NMR** (151 MHz, $CDCl_3$) δ 205.60, 53.62, 50.34, 31.87, 27.40. **HRMS (ESI)** calcd for $[M + Na]^+$ ($C_{12}H_{22}O_2S_4Na$): 349.0400; found: 349.0402.

Methyl-4-(((pyridin-3-ylthio)methyl)disulfaneyl)pentan-2-one (3a)

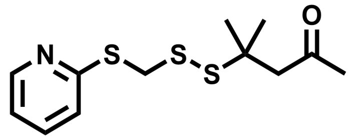

Colorless oil, yield: 71%, **Rf** = 0.5 (PE : EA = 10 : 1). **^{1}H NMR** (600 MHz, $CDCl_3$) δ 8.44 (d, *J* = 4.26 Hz, 1H), 7.49 (d, *J* = 6.6 Hz, 1H), 7.19 (d, *J* = 7.5 Hz, 1H), 7.02 (t, *J* = 6.0 Hz, 1H), 4.50 (s, 2H), 2.81 (s, 2H), 2.13 (s, 3H), 1.46 (s, 6H). **^{13}C NMR** (151 MHz, $CDCl_3$) δ 206.83, 156.11, 149.50, 136.27, 122.61, 120.17, 53.51, 49.47, 40.53, 32.06, 27.29. **GC-MS** (EI, 70ev): m/z(%) = 287 (M^+, 2), 189 (25), 156 (55), 124 (100), 112 (35), 99 (10), 78 (65). **HRMS (ESI)** calcd for $[M + H]^+$ ($C_{12}H_{18}NOS_3$): 288.0551; found: 288.0552.

4-((((5-Bromopyridin-2-yl)thio)methyl)disulfaneyl)-4-methylpentan-2-one (3b)

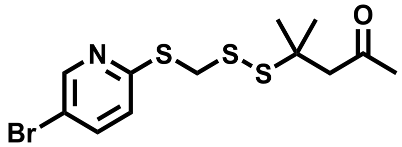

Colorless oil, yield: 64%, **Rf** = 0.5 (PE : EA = 10 : 1). **^{1}H NMR** (600 MHz, $CDCl_3$) δ 8.52 (s, 1H), 7.63 (d, *J* = 9.0 Hz, 1H), 7.11 (d, *J* = 9.6 Hz, 1H), 4.46 (s, 2H), 2.80 (s, 2H), 2.16 (s, 3H), 1.47 (s, 6H). **^{13}C NMR** (151 MHz, $CDCl_3$) δ 206.23, 155.05, 150.38, 138.82, 123.62, 116.87, 53.55, 49.51, 40.79, 32.00, 27.33. **GC-MS** (EI, 70ev): m/z(%) = 365 (M^+, 2), 110 (100), 91 (48), 68 (60). **HRMS (ESI)** calcd for $[M + H]^+$ ($C_{12}H_{17}BrNOS_3$): 365.9656; found: 365.9651.

4-Methyl-4-(((phenylthio)methyl)disulfaneyl)pentan-2-one (3c)

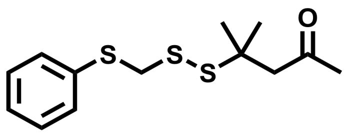

Colorless oil, yield: 64%, **Rf** = 0.3 (PE : EA = 40 : 1). **^{1}H NMR** (600 MHz, DMSO) δ 7.43 (d, *J* = 7.9 Hz, 2H), 7.31 (t, *J* = 7.2 Hz, 2H), 7.25 (t, *J* = 6.9 Hz, 1H), 4.36 (s, 2H), 2.74 (s, 2H), 2.06 (s, 3H), 1.33 (s, 6H). **^{13}C NMR** (151 MHz, DMSO) δ 206.62, 134.38, 129.77, 129.61, 127.28, 53.01, 49.71, 43.95, 32.13, 27.50. **GC-MS** (EI, 70ev): m/z(%) = 286 (M^+,5), 188 (5), 123 (100), 99 (26), 77 (8), 51 (5). **HRMS (ESI)** calcd for $[M + H]^+$ ($C_{13}H_{19}OS_3$): 287.0598; found: 287.0597.

4-((((4-Fluorophenyl)thio)methyl)disulfaneyl)-4-methylpentan-2-one (3d)

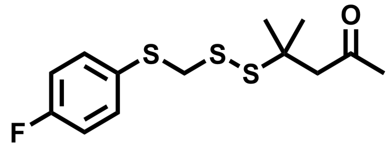

Colorless oil, yield: 51%, **Rf** = 0.3 (PE : EA = 40 : 1). **^{1}H NMR** (600 MHz, DMSO) δ 7.51 (d, *J* = 7.2 Hz, 2H), 7.21 (d, *J* = 18 Hz, 2H), 4.32 (s, 2H), 2.72 (s, 2H), 2.05 (s, 3H), 1.30 (s, 6H). **^{13}C NMR** (151 MHz, DMSO) δ 206.63, 162.00 (d, *J* = 244.62 Hz), 133.15 (*J* = 4.53 Hz), 129.69, 116.67 (*J* = 22.65 Hz), 53.02, 49.70, 44.99, 32.12, 27.49. **^{19}F NMR** (564 MHz, DMSO) δ -114.69. **GC-MS** (EI, 70ev): m/z(%) = 304 (M^+, 5), 206 (5), 141 (100), 99 (60), 83 (10). **HRMS (ESI)** calcd for $[M + H]^+$ ($C_{13}H_{18}FOS_3$): 305.0504; found: 305.0491.

4-((((4-Chlorophenyl)thio)methyl)disulfaneyl)-4-methylpentan-2-one (3e)

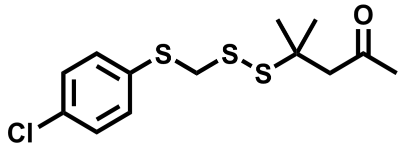

Colorless oil, yield: 59%, **Rf** = 0.3 (PE : EA = 40 : 1). **^{1}H NMR** (600 MHz, $CDCl_3$) δ 7.38 (d, *J* = 7.4 Hz, 2H), 7.28 (d, *J* = 7.2 Hz, 2H), 4.12 (s, 2H), 2.71 (s, 2H), 2.12 (s, 3H), 1.40 (s, 6H). **^{13}C NMR** (151 MHz, $CDCl_3$) δ 206.08, 133.58, 132.67, 132.16, 129.22, 53.40, 49.61, 45.74, 32.01, 27.31. **GC-MS** (EI, 70ev): m/z(%) = 320 (M^+, 8), 222 (8), 157 (100), 99 (90). **HRMS (ESI)** calcd for $[M - H]^-$ ($C_{13}H_{16}ClOS_3$): 319.0052; found: 319.0052.

4-((((2-Chlorophenyl)thio)methyl)disulfaneyl)-4-methylpentan-2-one (3f)

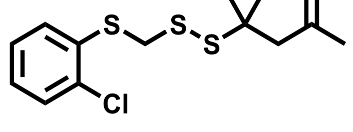

Colorless oil, yield: 64%, **Rf =** 0.3 (PE : EA = 40: 1). **^{1}H NMR** (600 MHz, $CDCl_3$) δ 7.44 (d, *J* = 53.8 Hz, 2H), 7.23 (d, *J* = 36.7 Hz, 2H), 4.20 (s, 2H), 2.74 (s, 2H), 2.13 (s, 3H), 1.42 (s, 6H). **^{13}C NMR** (151 MHz, $CDCl_3$) δ 206.10, 134.84, 133.30, 131.02, 129.93, 128.10, 127.22, 53.46, 49.57, 43.97, 31.98, 27.28. **GC-MS** (EI, 70ev): m/z(%)

= 268 (M^+, 3), 99 (100). **HRMS (ESI)** calcd for $[M - H]^-$ ($C_{13}H_{16}ClOS_3$): 319.0052; found: 319.0052.

4-((((3-Chlorophenyl)thio)methyl)disulfaneyl)-4-methylpentan-2-one (3g)

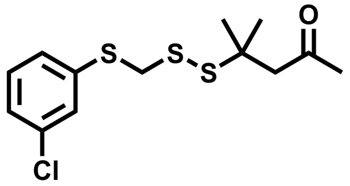

Colorless oil, yield: 65%, **Rf** = 0.3 (PE : EA = 40:1). **^{1}H NMR** (600 MHz, $CDCl_3$) δ 7.42 (d, *J* = 22.4 Hz, 1H), 7.31 (d, *J* = 7.0 Hz, 1H), 7.27 – 7.19 (m, 2H), 4.16 (s, 2H), 2.75 (s, 2H), 2.13 (s, 3H), 1.42 (s, 6H). **^{13}C NMR** (151 MHz, $CDCl_3$) δ 206.16, 134.78, 130.06, 129.89, 128.51, 128.27, 127.34, 53.40, 49.66, 45.02, 32.02, 27.30. **GC-MS** (EI, 70ev): m/z(%) = 320 (M^+, 3), 157 (60), 99(100), 75 (10). **HRMS (ESI)** calcd for $[M - H]^-$ ($C_{13}H_{16}ClOS_3$): 319.0052; found: 319.0052.

4-Methyl-4-(((*p*-tolylthio)methyl)disulfaneyl)pentan-2-one (3h)

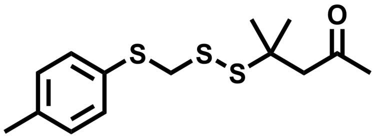

Yellow oil, yield: 61%, **Rf** = 0.3 (PE : EA = 40:1). **^{1}H NMR** (600 MHz, $CDCl_3$) δ 7.37 (d, *J* = 7.8 Hz, 2H), 7.13 (d, *J* = 8.4 Hz, 2H), 4.11 (s, 2H), 2.71 (s, 2H), 2.32 (s, 3H), 2.11 (s, 3H), 1.39 (s, 6H). **^{13}C NMR** (151 MHz, $CDCl_3$) δ 206.25, 137.71, 131.55, 130.48, 129.84, 53.40, 49.50, 46.37, 32.01, 27.25, 21.07. **GC-MS** (EI, 70ev): m/z(%) = 300 (M^+, 4), 202 (4), 137 (100), 128(8), 99(5), 91(11), 77(5). **HRMS (ESI)** calcd for $[M + H]^+$ ($C_{14}H_{21}OS_3$): 301.0755; found: 301.0750.

4-((((4-(*tert*-Butyl)phenyl)thio)methyl)disulfaneyl)-4-methylpentan-2-one (3i)

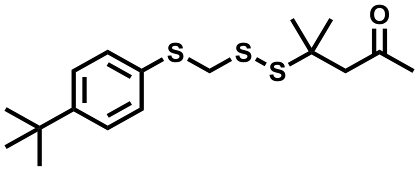

Colorless oil, yield: 85%, **Rf** = 0.3 (PE : EA = 40 : 1). **^{1}H NMR** (600 MHz, $CDCl_3$) δ 7.40 (d, *J* = 9.0 Hz, 2H), 7.34 (d, *J* = 7.8 Hz, 2H), 4.14 (s, 2H), 2.72 (s, 2H), 2.12 (s, 3H), 1.39 (s, 6H), 1.30 (s, 9H). **^{13}C NMR** (151 MHz, $CDCl_3$) δ 206.21, 150.85, 130.97, 130.66, 126.12, 53.42, 49.51, 46.12, 34.55, 32.03, 31.22, 27.24. **GC-MS** (EI, 70ev): m/z(%) = 342 (M^+, 5), 244 (3), 179 (100), 128(78), 57(100). **HRMS (ESI)** calcd for $[M + H]^+$ ($C_{17}H_{27}OS_3$): 343.1224; found: 343.1194.

4-Methyl-4-((((4-(methylthio)phenyl)thio)methyl)disulfaneyl)pentan-2-one (3j)

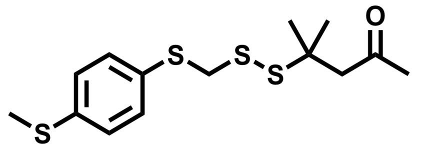

Colorless oil, yield: 68%, **R_f** = 0.4 (PE : EA = 10 : 1). **^{1}H NMR** (600 MHz, DMSO) δ 7.39 (d, *J* = 10.2 Hz, 2H), 7.24 (d, *J* = 9.0 Hz, 2H), 4.31 (s, 2H), 2.73 (s, 2H), 2.45 (s, 3H), 2.06 (s, 3H), 1.31 (s, 6H). **^{13}C NMR** (151 MHz, DMSO) δ 206.63, 138.04, 131.23, 130.12, 127.04, 53.02, 49.71, 44.70, 32.12, 27.51, 15.15. **GC-MS** (EI, 70ev): m/z(%) = 332 (M^+, 6), 234 (2), 169 (100), 155 (15), 125 (40), 77(5). **HRMS (ESI)** calcd for $[M + H]^+$ ($C_{14}H_{21}OS_4$): 333.0475; found: 333.0463.

4-((((4-Methoxyphenyl)thio)methyl)disulfaneyl)-4-methylpentan-2-one (3k)

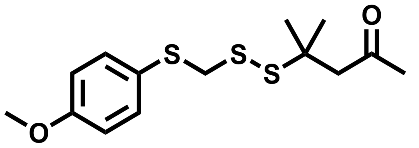

Colorless oil, yield: 53%, **Rf** = 0.4 (PE : EA = 20 : 1). **^{1}H NMR** (600 MHz, DMSO) δ 7.42 (d, *J* = 12.0 Hz, 2H), 6.93 (d, *J* = 12.0 Hz, 2H), 4.21 (s, 2H), 3.74 (s, 3H), 2.69 (s, 2H), 2.05 (s, 3H), 1.29 (s, 6H). **^{13}C NMR** (151 MHz, DMSO) δ 206.59, 159.67, 133.92, 124.25, 115.27, 55.70, 52.97, 49.65, 46.29, 32.11, 27.45. **GC-MS** (EI, 70 ev): m/z(%) = 316 (M^+, 4), 153 (100), 139 (15), 109 (15), 77 (4). **HRMS (ESI)** calcd for $[M + H]^+$ ($C_{14}H_{21}O_2S_3$): 317.0704; found: 317.0674.

4-Methyl-4-((((4-(trifluoromethyl)phenyl)thio)methyl)disulfaneyl)pentan-2-one (3l)

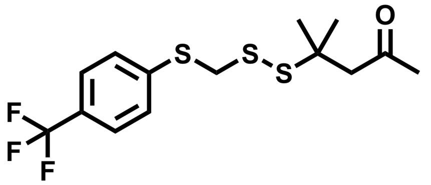

Colorless oil, yield: 69%, **Rf** = 0.3 (PE : EA = 40 : 1). **^{1}H NMR** (600 MHz, $CDCl_3$) δ 7.56 (d, *J* = 7.8 Hz, 2H), 7.48 (d, *J* = 7.9 Hz, 2H), 4.21 (s, 2H), 2.74 (s, 2H), 2.13 (s, 3H), 1.44 (s, 6H). **^{13}C NMR** (151 MHz, $CDCl_3$) δ 205.99, 139.62, 128.91, 125.86, 125.83, 123.94 (CF_3, *J* = 271.95 Hz), 53.44, 49.71, 44.12, 31.98, 27.38. **^{19}F NMR** (564 MHz, $CDCl_3$) δ -62.60. **GC-MS** (EI, 70ev): m/z(%) = 354 (M^+, 3), 191 (52), 171 (14), 99 (100), 83 (2), 57 (3). **HRMS (ESI)** calcd for $[M + Na]^+$ ($C_{14}H_{17}F_3NaOS_3$): 377.0291; found: 377.0320.

4-((((3,5-Bis(trifluoromethyl)phenyl)thio)methyl)disulfaneyl)-4-methylpentan-2-o

ne (3m)

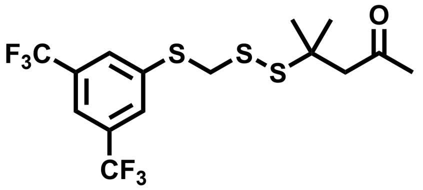

Colorless oil, yield: 52%, **Rf** = 0.3 (PE : EA = 40 :1). **^{1}H NMR** (600 MHz, DMSO) δ 8.08 (s, 2H), 7.92 (s, 1H), 4.62 (s, 2H), 2.76 (s, 2H), 2.02 (s, 3H), 1.36 (s, 6H). **^{13}C NMR** (151 MHz, DMSO) δ 206.53, 139.30, 131.33 (d, *J* = 33.22 Hz), 129.08, 123.28 (d, *J* = 277.84 Hz), 120.13 (t, *J* = 3.02 Hz), 53.08, 49.81, 42.22, 32.04, 27.49. **^{19}F NMR** (564 MHz, DMSO) δ -61.52. **GC-MS** (EI, 70ev): m/z(%) = 422 (M^+, 5), 410 (8), 259 (45), 239 (60), 99 (100), 57 (15). **HRMS (ESI)** calcd for $[M + Na]^+$ ($C_{15}H_{16}F_6NaOS_3$): 445.0165; found: 445.0204.

4-((((4-Fluoro-2-methylphenyl)thio)methyl)disulfaneyl)-4-methylpentan-2-one (3n)

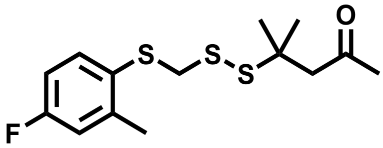

Colorless oil, yield: 55%, **Rf** = 0.3 (PE : EA = 40 : 1). **^{1}H NMR** (600 MHz, $CDCl_3$) δ 7.48 (dd, *J* = 8.3, 5.6 Hz, 1H), 6.93 (t, J = 12.0 Hz, 1H), 6.89 (t, *J* = 6 Hz, 1H), 4.06 (s, 3H), 2.70 (s, 3H), 2.45 (s, 3H), 2.12 (s, 3H), 1.38 (s, 6H). **^{13}C NMR** (151 MHz, DMSO) δ 206.13, 162.48 (d, *J* = 249.15 Hz), 142.68, 134.63 (d, *J* = 7.55 Hz), 128.31 (d, *J* = 34.73 Hz), 117.35 (d, *J* = 21.14 Hz), 113.63 (d, *J* = 21.14 Hz), 53.34, 49.54, 46.33, 32.00, 27.21, 21.00. **^{19}F NMR** (564 MHz, $CDCl_3$) δ -113.92. **GC-MS** (EI, 70ev): m/z(%) = 318 (M^+, 4), 220 (13), 155 (100), 83 (5). **HRMS (ESI)** calcd for $[M + H]^+$ ($C_{14}H_{20}FOS_3$): 319.0660; found: 319.0661.

4-((((4-((*tert*-Butyldimethylsilyl)oxy)-2-methylphenyl)thio)methyl)disulfaneyl)-4-methylpentan-2-one (3o)

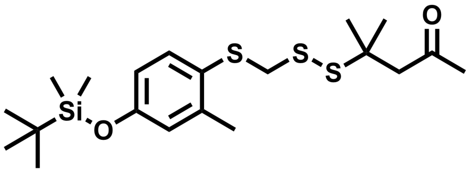

Colorless oil, yield: 79%, **Rf** = 0.4 (PE : EA = 20: 1). **^{1}H NMR** (600 MHz, $CDCl_3$) δ 7.39 (d, *J* = 8.4 Hz, 1H), 6.72 (s, 1H), 6.65 (d, *J* = 12.0 Hz, 1H), 4.02 (s, 2H), 2.68 (s, 2H), 2.41 (s, 3H), 2.11 (s, 3H), 1.35 (s, 6H), 0.97 (s, 9H), 0.19 (s, 6H). **^{13}C NMR** (151 MHz, $CDCl_3$) δ 206.61, 156.01, 142.25, 135.28, 124.40, 122.11, 118.23, 53.31, 49.42,

46.98, 32.00, 25.62, 21.05, 18.16, -4.42. **GC-MS** (EI, 70 ev): m/z(%) = 430 (M^+, 3), 267 (100), 253 (7), 211 (7), 115 (6), 73 (68). **HRMS (ESI)** calcd for $[M + H]^+$ ($C_{20}H_{35}O_2S_3Si$): 431.1568; found: 431.1573.

4-Methyl-4-((1-(phenylthio)ethyl)disulfaneyl)pentan-2-one (3p)

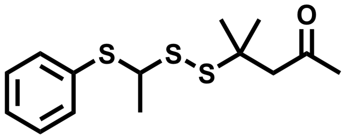

Colorless oil, yield: 65%, **Rf** = 0.3 (PE : EA = 40 : 1). **^{1}H NMR** (600 MHz, DMSO) 7.54 (d, J = 8.4 Hz, 2H), 7.38 (t, J = 14.7 Hz, 2H), 7.34 (t, J = 15.0 Hz, 1H), 4.42-4.38 (m, J = 18.3 Hz, 1H), 2.63 (s, 2H), 2.01 (s, 3H), 1.58 (s, 3H), 1.23 (s, 6H). **^{13}C NMR** (151 MHz, DMSO) δ 206.51, 133.07, 129.57, 128.63, 54.11, 53.01, 49.56, 32.06, 27.57, 22.01. **GC-MS** (EI, 70ev): m/z(%) = 300 (M^+, 1), 137 (100), 109 (18), 59 (12). **HRMS (ESI)** calcd for $[M + H]^+$ ($C_{14}H_{21}OS_3$): 301.0755; found: 301.0760.

4-Methyl-4-((phenyl(phenylthio)methyl)disulfaneyl)pentan-2-one (3q)

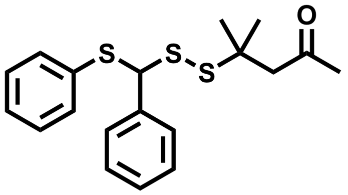

Colorless oil, yield: 56%, **Rf** = 0.3 (PE : EA = 20 : 1). **^{1}H NMR** (600 MHz, DMSO) δ 7.54 (d, J = 7.2 Hz, 2H), 7.45 (d, J = 7.2 Hz, 2H), 7.35 (t, J = 13.8 Hz, 4H), 7.33-7.29 (m, J = 21 Hz, 2H), 5.44 (s, 1H), 2.53 (s, 2H), 1.97 (s, 3H), 1.17 (s, 6H). **^{13}C NMR** (151 MHz, DMSO) δ 206.50, 139.43, 133.86, 132.53, 129.59, 128.99, 128.79, 128.57, 62.13, 53.08, 49.72, 32.00, 27.53, 27.48. **GC-MS** (EI, 70ev): m/z(%) = 300 (M^+, 1), 137 (100), 109 (18), 59 (12). **HRMS (ESI)** calcd for $[M + Na]^+$ ($C_{19}H_{22}NaOS_3$): 385.0730; found: 385.0732.

4-Methyl-4-((tetrahydrothiophen-2-yl)disulfaneyl)pentan-2-one (3r)

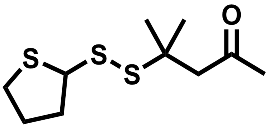

Colorless oil, yield: 72%, **Rf** = 0.5 (PE : EA = 40 : 1). **^{1}H NMR** (600 MHz, DMSO) δ 4.55 (s, 1H), 2.95 (t, J = 18.0 Hz, 1H), 2.75 (s, 3H), 2.32 - 2.29 (d, J = 18.0 Hz, 1H), 2.07 (s, 3H), 2.02 - 2.01 (m, 2H), 2.01 - 1.98 (m, 1H), 1.33 (s, 6H). **^{13}C NMR** (151 MHz, DMSO) δ 206.69, 58.66, 53.15, 49.53, 37.61, 33.25, 32.14, 27.78, 27.49. **GC-MS** (EI, 70ev): m/z(%) = 250 (M^+, 1), 87(100), 53(2). **HRMS (ESI)** calcd for [M

+ Na]+ ($C_{10}H_{18}NaOS_3$): 273.0417; found: 273.0421.

4-Methyl-4-((tetrahydro-2H-thiopyran-2-yl)disulfaneyl)pentan-2-one (3s)

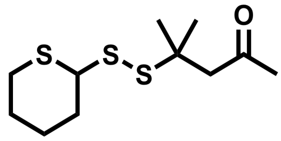

Colorless oil, yield: 82%, **Rf** = 0.5 (PE : EA = 40 : 1). **^{1}H NMR** (600 MHz, $CDCl_3$) δ 4.02 (t, *J* = 7.8 Hz, 1H), 2.99 - 2.95 (m, 1H), 2.79 (s, 2H), 2.44 - 2.42 (m, 1H), 2.29 - 2.26 (m, 1H), 2.16 (s, 3H), 2.09 - 2.05 (m, 1H), 1.89 - 1.80 (m, 2H), 1.74 - 1.70 (m, 1H), 1.52 (d, *J* = 18.0 Hz, 1H), 1.43 (s, 6H). **^{13}C NMR** (151 MHz, $CDCl_3$) δ 205.86, 53.13, 50.13, 50.00, 31.98, 30.36, 27.24, 26.52, 26.32, 21.27. **GC-MS** (EI, 70 ev): m/z(%) = 264 (M+, 5), 207 (10), 101 (100), 99 (20). **HRMS (ESI)** calcd for [M + Na]+ ($C_{11}H_{20}NaOS_3$): 287.0574; found: 287.0577.

4-(((Isopropylthio)methyl)disulfaneyl)-4-methylpentan-2-one (3t)

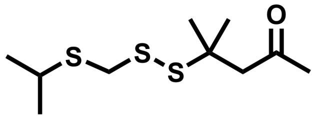

Colorless oil, yield: 60%, **Rf** = 0.5 (PE : EA = 40 : 1). **^{1}H NMR** (600 MHz, $CDCl_3$) δ 3.86 (s, 2H), 3.19 - 3.15 (m, 1H), 2.77 (s, 2H), 2.16 (s, 3H), 1.44 (s, 6H), 1.30 (d, *J* = 6.9 Hz, 6H). **^{13}C NMR** (151 MHz, $CDCl_3$) δ 206.30, 53.52, 49.43, 42.33, 35.08, 32.05, 27.31, 23.09. **GC-MS** (EI, 70ev): m/z(%) = 252 (M^+, 3), 154 (14), 99 (30), 89 (100), 79 (5), 55 (40). **HRMS (ESI)** calcd for [M + Na]+ ($C_{10}H_{20}NaOS_3$): 275.0574; found: 275.0574.

4-((1-(Ethylthio)ethyl)disulfaneyl)-4-methylpentan-2-one (3u)

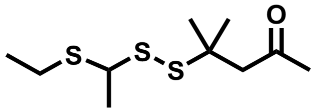

Colorless oil, yield: 55%, **Rf** = 0.4 (PE : EA = 40 : 1). **^{1}H NMR** (600 MHz, $CDCl_3$) δ 3.89 (d, *J* = 6.2 Hz, 1H), 2.80 – 2.73 (m, 4H), 2.14 (s, 3H), 1.62 (t, *J* = 13.5 Hz, 3H), 1.41 (s, 6H), 1.28 (t, *J* = 7.0 Hz, 3H). **^{13}C NMR** (151 MHz, $CDCl_3$) δ 206.21, 53.66, 51.56, 49.13, 32.05, 27.63, 27.08, 25.75, 22.05, 14.64. **GC-MS** (EI, 70ev): m/z(%) = 252 (M^+, 1), 89 (100), 61 (20). **HRMS (ESI)** calcd for [M + Na]+ ($C_{10}H_{20}NaOS_3$): 275.0574; found: 275.0574.

4-((1-(Isobutylthio)-2-methylpropyl)disulfaneyl)-4-methylpentan-2-one (3v)

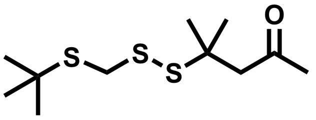

Colorless oil, yield: 57%, **Rf** = 0.5 (PE : EA = 40 : 1). **^{1}H NMR** (600 MHz, $CDCl_3$) δ 3.89 (s, 2H), 2.77 (s, 2H), 2.15 (s, 3H), 1.44 (s, 6H), 1.37 (s, 9H). **^{13}C NMR** (151 MHz, $CDCl_3$) δ 206.16, 53.51, 49.45, 44.01, 40.61, 32.06, 31.19, 27.36. **GC-MS** (EI, 70ev): m/z(%) = 266 (M^+, 3), 168 (3), 103 (63), 57 (100). **HRMS (ESI)** calcd for $[M - H]^-$ ($C_{11}H_{21}OS_3$): 265.0755; found: 265.0757.

4-(((((3s,5s,7s)-Adamantan-1-yl)thio)methyl)disulfaneyl)-4-methylpentan-2-one (3w)

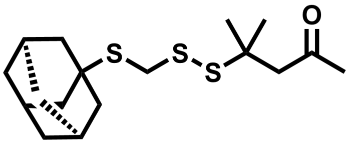

Colorless oil, yield: 64%, **Rf** = 0.5 (PE : EA = 40 : 1). **^{1}H NMR** (600 MHz, DMSO) δ 3.94 (s, 2H), 2.77 (s, 2H), 2.08 (s, 3H), 1.99 (s, 3H), 1.83 (s, 6H), 1.63 (s, 6H), 1.35 (s, 6H). **^{13}C NMR** (151 MHz, DMSO) δ 206.65, 53.18, 49.62, 46.16, 43.65, 38.09, 35.99, 32.17, 29.60, 27.66. **GC-MS** (EI, 70ev): m/z(%) = 344 (M^+, 3), 181 (18), 135 (100). **HRMS (ESI)** calcd for $[M + H]^+$ ($C_{17}H_{29}OS_3$): 345.1381; found:345.1381.

4-((((2-Ethylhexyl)thio)methyl)disulfaneyl)-4-methylpentan-2-one (3x)

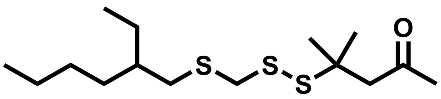

Colorless oil, yield: 51%, **Rf** = 0.5 (PE : EA = 15 : 1). **^{1}H NMR** (600 MHz, $CDCl_3$) δ 3.83 (s, 2H), 2.78 (s, 2H), 2.68 (d, *J* = 6.3 Hz, 2H), 2.16 (s, 3H), 1.46 – 1.43 (m, 7H), 1.37 - 1.20 (m, 8H), 0.92 – 0.86 (m, 6H). **^{13}C NMR** (151 MHz, $CDCl_3$) δ 206.54, 53.60, 49.54, 44.49, 39.19, 36.75, 32.46, 32.20, 28.92, 27.38, 25.65, 23.03, 14.16, 10.89. **GC-MS** (EI, 70ev): m/z(%) = 322 (M^+, 6), 159 (80), 111 (35), 69 (100), 61 (76). **HRMS (ESI)** calcd for $[M + Na]^+$ ($C_{15}H_{30}NaNOS_3$): 345.1356; found: 345.1357.

3,5-Dimethyl-4-((((phenylthio)methyl)disulfaneyl)methyl)isoxazole (5a)

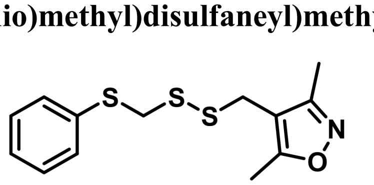

Colorless oil, yield: 82%, **Rf** = 0.3 (PE : EA = 10 : 1). **^{1}H NMR** (600 MHz, DMSO) δ 7.39 (d, *J* = 6.0 Hz, 2H), 7.34(t, *J* = 15.0 Hz, 2H), 7.24(t, *J* = 16.8 Hz, 1H), 4.29 (s, 2H), 3.85 (s, 2H), 2.28 (s, 3H), 2.16 (s, 3H). **^{13}C NMR** (151 MHz, DMSO) δ 167.48,

159.58, 134.41, 129.62, 129.48, 127.19, 110.80, 42.02, 29.86, 11.12, 10.12. **GC-MS** (EI, 70ev): m/z(%) = 297 (M^+, 20), 207 (10), 123 (100), 110 (50), 68 (49). **HRMS (ESI)** calcd for $[M + Na]^+$ ($C_{13}H_{15}NNaOS_3$): 320.0213; found:320.0200.

4-(((1-(Ethylthio)ethyl)disulfaneyl)methyl)-3,5-dimethylisoxazole (5b)

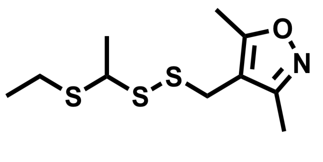

Yellow oil, yield: 79%, **Rf** = 0.3 (PE : EA = 10 : 1). **^{1}H NMR** (600 MHz, $CDCl_3$) δ 3.72 (s, 2H), 2.67 - 2.59 (m, 2H), 2.38 (s, 3H), 2.27 (s, 3H), 1.71 - 1.67 (m, 1H), 1.57 (d, J = 6.8 Hz, 3H), 1.25 - 1.23 (m, 3H). **^{13}C NMR** (151 MHz, $CDCl_3$) δ 166.95, 159.39, 110.24, 51.35, 31.58, 25.86, 22.01, 14.55, 11.27, 10.17. **GC-MS** (EI, 70ev): m/z(%) = 263 (M^+, 1), 175 (17), 110 (8), 89 (100), 68 (20), 61 (25). **HRMS (ESI)** calcd for $[M + H]^+$ ($C_{10}H_{18}NOS_3$): 264.0551; found: 264.0552.

1-(1-(Ethylthio)ethyl)-2-(4-methoxybenzyl)disulfane (5c)

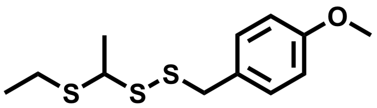

Colorless oil, yield: 77%, **Rf** = 0.5 (PE : EA = 40 : 1). **^{1}H NMR** (600 MHz, $CDCl_3$) δ 7.24 (d, J = 12.0 Hz, 2H), 6.84 (d, J = 5.9 Hz, 2H), 3.96 - 3.90 (m, 2H), 3.79 (s, 3H), 3.57 - 3.55 (m, 1H), 2.63 (s, 2H), 1.54 (d, J = 4.2 Hz, 3H), 1.23 - 1.22 (m, 3H). **^{13}C NMR** (151 MHz, $CDCl_3$) δ 159.88, 130.42, 129.51, 113.94, 55.25, 50.92, 43.76, 25.91, 22.01, 14.69. **GC-MS** (EI, 70ev): m/z(%) = 271 (M^+, 1), 121 (100), 89 (95), 77 (5), 61 (23). **HRMS (ESI)** calcd for $[M + H]^+$ ($C_{12}H_{19}OS_3$): 275.0598; found: 275.0599.

1-Ethyl-2-(1-(ethylthio)ethyl)disulfane (5d)[7]

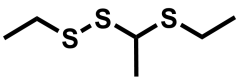

Colorless oil, yield: 63%, **Rf** = 0.8 (PE). **^{1}H NMR** (600 MHz, CDCl$_3$) δ 3.94 (d, J = 6.9 Hz, 1H), 2.85-2.73 (m, 3H), 2.72 – 2.64 (m, 1H), 1.65 (d, J = 6.8 Hz, 3H), 1.35-1.29 (t, J = 7.3 Hz, 3H), 1.29-1.24 (d, J = 7.4 Hz, 3H). **^{13}C NMR** (101 MHz, $CDCl_3$) δ 51.43, 33.68, 25.87, 22.29, 14.57. **GC-MS** (EI, 70ev): m/z (%) = 182 (M^+, 1), 121 (2), 89 (100), 61 (41). **HRMS (ESI)** calcd for $[M + Na]^+$ ($C_6H_{14}OS_3Na$): 205.0155; found: 205.0155.

Methyl(*E*)-2-(2-(((1-(ethylthio)ethyl)disulfaneyl)methyl)phenyl)-2-(methoxy-imino)acetate (5e)

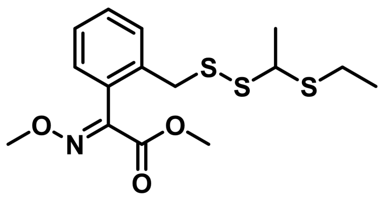

Colorless oil, yield: 74%, **Rf** = 0.5 (PE : EA = 8 : 1). ^{1}**H NMR** (600 MHz, $CDCl_3$) δ 7.40 (d, *J* = 6.6 Hz, 1H), 7.35 - 7.33 (m, 2H), 7.15 (d, *J* = 6.0 Hz, 1H), 4.04 (s, 3H), 3.86 (s, 3H), 2.62 - 2.60 (m, 2H), 1.71 - 1.69 (m, 1H), 1.50 (d, *J* = 6.6 Hz, 3H), 1.25 - 1.23 (m, 3H). ^{13}**C NMR** (151 MHz, $CDCl_3$) δ 163.28, 149.38, 135.80, 130.24, 129.25, 128.87, 127.38, 109.99, 63.76, 52.96, 51.11, 42.74, 25.85, 22.17, 14.66. **GC-MS** (EI, 70ev): m/z(%) = 359 (M^+, 1), 271 (4), 116 (8), 89 (100), 61 (13). **HRMS (ESI)** calcd for $[M + Na]^+$ ($C_{15}H_{21}NNaO_3S_3$): 382.0581; found: 382.0591.

2-(3-((1-(Ethylthio)ethyl)disulfaneyl)propyl)isoindoline-1,3-dione (5f)

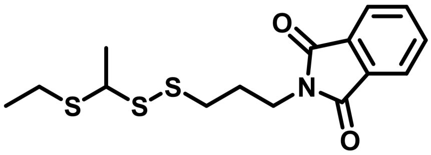

Na

Colorless oil, yield: 50%, **Rf** = 0.5 (PE : EA = 10 : 1).^{1}**H NMR** (600 MHz, $CDCl_3$) δ 7.88 – 7.79 (m, 2H), 7.73 – 7.68 (m, 2H), 3.95 (d, *J* = 6.8 Hz, 1H), 3.82 – 3.76 (m, 2H), 2.78 (dp, *J* = 18.4, 6.2 Hz, 2H), 2.73 – 2.67 (m, 1H), 2.63 (dd, *J* = 12.8, 7.1 Hz, 1H), 2.09 (p, *J* = 6.9 Hz, 2H), 1.62 (d, *J* = 6.8 Hz, 3H), 1.23 (d, *J* = 8.2 Hz, 3H). ^{13}**C NMR** (151 MHz, $CDCl_3$) δ 168.21, 133.95, 132.04, 123.23, 51.37, 36.94, 36.81, 28.36, 25.83, 22.23, 14.50. **HRMS (ESI)** calcd for $[M + Na]^+$ ($C_{15}H_{19}NNaO_2S_3$): 364.0476; found: 364.0443.

5-(3,5-Dichlorophenyl)-3-(4-(((1-(ethylthio)ethyl)disulfaneyl)methyl)-3-methylphenyl)-5-(trifluoromethyl)-4,5-dihydroisoxazole (5g)

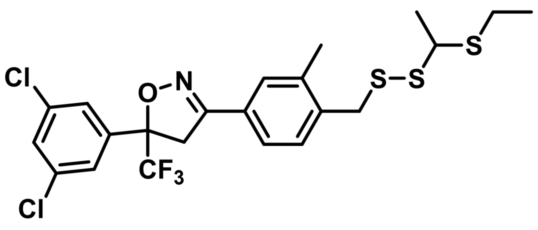

Colorless oil, yield: 57%, **Rf** = 0.6 (PE : EA = 10 : 1). ^{1}**H NMR** (600 MHz, $CDCl_3$) δ 7.50 (s, 3H), 7.42 (d, *J* = 13.8 Hz, 2H), 7.29 (d, *J* = 9.0 Hz, 1H), 4.19 -4.22 (m, 1H), 4.08 (s, 1H), 4.04 (d, *J* = 6.0 Hz, 2H), 3.68 (d, *J* = 12.0 Hz, 1H), 2.71 - 2.67 (m, 1H), 2.63 - 2.59 (m, 2H), 2.42 (s, 3H), 1.68 (d, *J* = 9.0 Hz, 3H), 1.26 - 1.24 (m, 3H). ^{13}**C NMR** (151 MHz, $CDCl_3$) δ 155.72, 139.19, 137.72, 137.65, 135.56, 131.22, 131.13, 129.65, 128.88, 127.06, 125.27, 124.58, 51.85, 44.19, 40.90, 25.93, 22.13, 19.22,

14.46. **^{19}F NMR** (564 MHz, $CDCl_3$) δ -79.37. **HRMS (ESI)** calcd for $[M + Na]^+$ ($C_{22}H_{22}Cl_2F_3NaNOS_3$): 562.0090; found: 562.0128.

2-((((4-((4-Fluorobenzyl)oxy)-3-methoxybenzyl)disulfaneyl)methyl)thio)pyridine (5h)

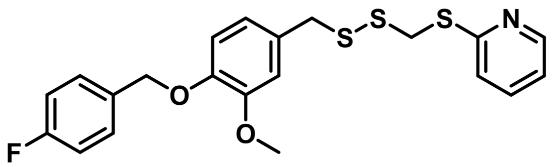

Colorless oil, yield: 80%, **Rf** = 0.3 (PE : EA = 10 : 1). **^{1}H NMR** (600 MHz, $CDCl_3$) δ 8.45 (d, *J* = 4.8 Hz, 1H), 7.45 (t, *J* = 18.0 Hz,1H), 7.38 (t, *J* = 18.0 Hz, 2H), 7.15 (d, *J* = 8.4 Hz, 1H), 7.03 (t, *J* = 16.8 Hz, 2H), 6.98 (s, 2H), 6.88 (d, *J* = 10.2 Hz, 1H), 6.78 (d, *J* = 12 Hz, 1H), 5.07 (s, 2H), 4.38 (s, 2H), 3.88 (s, 2H), 3.84 (s, 3H). **^{13}C NMR** (151 MHz, $CDCl_3$) δ 163.21, 161.59, 158.85, 149.33, 147.14, 135.96, 132.91, 131.23, 129.14, 122.15, 121.05, 119.58, 115.45, 114.10, 112.69, 74.56, 70.49, 55.84, 34.35. **^{19}F NMR** (564 MHz, $CDCl_3$) δ -114.56. **HRMS (ESI)** calcd for $[M + Na]^+$ ($C_{21}H_{20}FNNaO_2S_3$): 456.0538; found: 456.0536.

5-(3-((1-(Ethylthio)ethyl)disulfaneyl)propoxy)benzo[*d*][1,3]dioxole (5i)

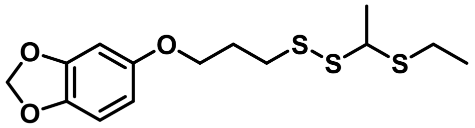

Colorless oil, yield: 49%, **Rf** = 0.5 (PE). **^{1}H NMR** (600 MHz, $CDCl_3$) δ 6.68 (d, *J* = 7.5 Hz, 1H), 6.47 (s, 1H), 6.30 (d, *J* = 7.5 Hz, 1H), 5.90 (s, 2H), 3.97 (s, 2H), 3.94 (d, *J* = 5.7 Hz, 1H), 2.92 (s, 2H), 2.70 (dd, *J* = 37.6, 6.7 Hz, 2H), 2.13 (s, 2H), 1.65 (d, *J* = 5.4 Hz, 3H), 1.29 - 1.25 (m, 3H). **^{13}C NMR** (151 MHz, $CDCl_3$) δ 154.27, 148.22, 141.68, 107.91, 105.72, 101.09, 98.08, 66.82, 51.35, 36.13, 29.03, 25.85, 22.25, 14.53. **GC-MS** (EI, 70ev): m/z(%) = 332 (M^+, 2), 270 (2), 137 (8), 89 (100), 61 (15). **HRMS (ESI)** calcd for $[M - H]^-$ ($C_{14}H_{19}O_3S_3$): 331.0497; found: 331.0497.

2-(((Phenyldisulfaneyl)methyl)thio)pyridine (9)

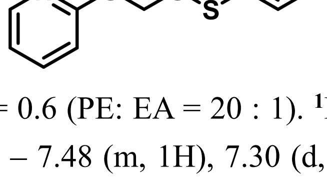

Colorless oil, yield: 42%, **Rf** = 0.6 (PE: EA = 20 : 1). **^{1}H NMR** (600 MHz, $CDCl_3$) δ 8.55 (d, *J* = 3.5 Hz, 1H), 7.58 – 7.48 (m, 1H), 7.30 (d, *J* = 6.4 Hz, 1H), 7.28 (d, *J* = 6.9 Hz, 2H), 7.23 – 7.18 (m, 1H), 7.08 (t, *J* = 7.6 Hz, 2H), 4.16 (s, 2H). **^{13}C NMR** (151 MHz, $CDCl_3$) δ 160.99, 149.33, 139.50, 136.44, 129.09, 128.55, 126.35, 123.05,

121.18, 44.74. **GC-MS** (EI, 70ev): m/z(%) = 265 (M^+, 8), 186 (60), 126 (18), 111 (20), 69 (33), 57 (32). **HRMS (ESI)** calcd for $[M + H]^+$ ($C_{12}H_{13}NS_3$): 266.0132; found: 266.0137.

References

1. Gao, W., Tian, J., Shang, Yu. & Jiang, X. Steric and stereoscopic disulfide construction for cross–linkage via *N*–dithiophthalimides. *Chem. Sci*. **11**, 3903-3908 (2020).
2. Wu, Z. &Pratt, D. A. A divergent strategy for site-selective radical disulfuration of carboxylic acids with trisulfide-1,1-dioxides. *Angew. Chem. Int. Ed*. **60**, 15598-15605 (2021).
3. Asanuma, H. & Kanemoto, K. Amination of *N*-(organodithio)phthalimides for the modular synthesis of aminodisulfides. *Org. Lett*., **26**, 438-443 (2024).
4. Gong, K., Zhou, Y. & Jiang, X. From symmetrical tetrasulfides to trisulfide dioxides via photocatalysis. *Green Chem*. **23**, 9865-9869 (2021).
5. Tian, Q. & Li, Y. Design of a bilateral disulfurating reagent for unsymmetrical polysulfidation. *Angew. Chem. Int. Ed*. **62**, e202302861 (2023).
6. Wang, X., Wang, G., Zhao, J., Zhu, Z. & Rao, J. Main–chain sulfonium–containing homopolymers with negligible hemolytic toxicity for eradication of bacterial and fungal biofilms. *ACS Macro Letters*. **10**, 1643–1649 (2021).
7. Li, J., Schieberle, P. & Steinhaus, M. Characterization of the major odor–active compounds in thai durian (*Durio zibethinus* L. ‘Monthong’) by aroma extract dilution analysis and headspace gas chromatography–olfactometry. *J. Agric. Food Chem*. **60**, 11253–11262 (2012).

12.NMR spectra

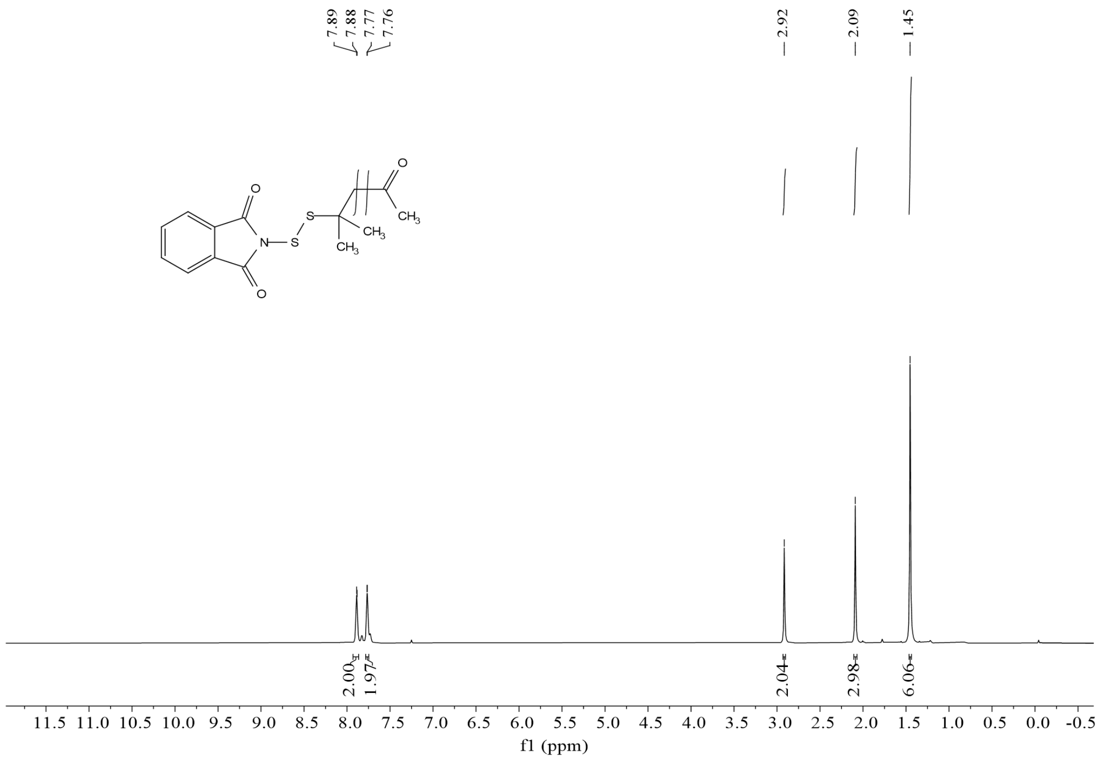

^{1}H NMR of compound 2a

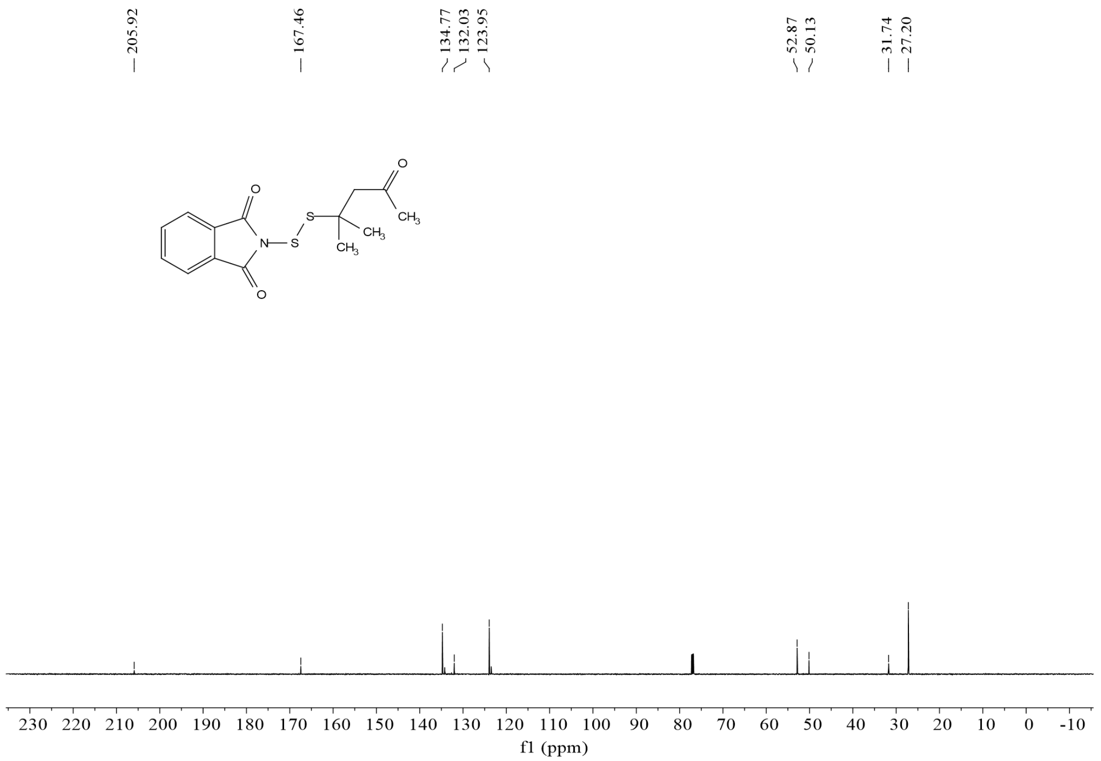

^{13}C NMR of compound 2a

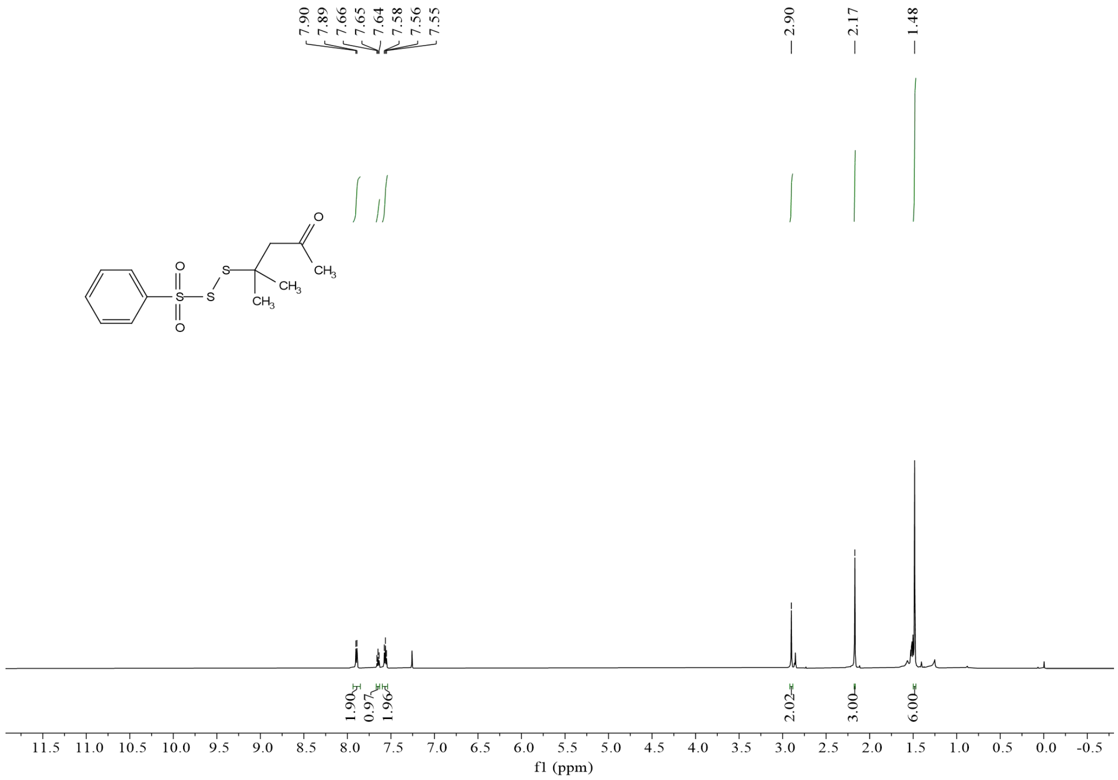

^{1}H NMR of compound 2b

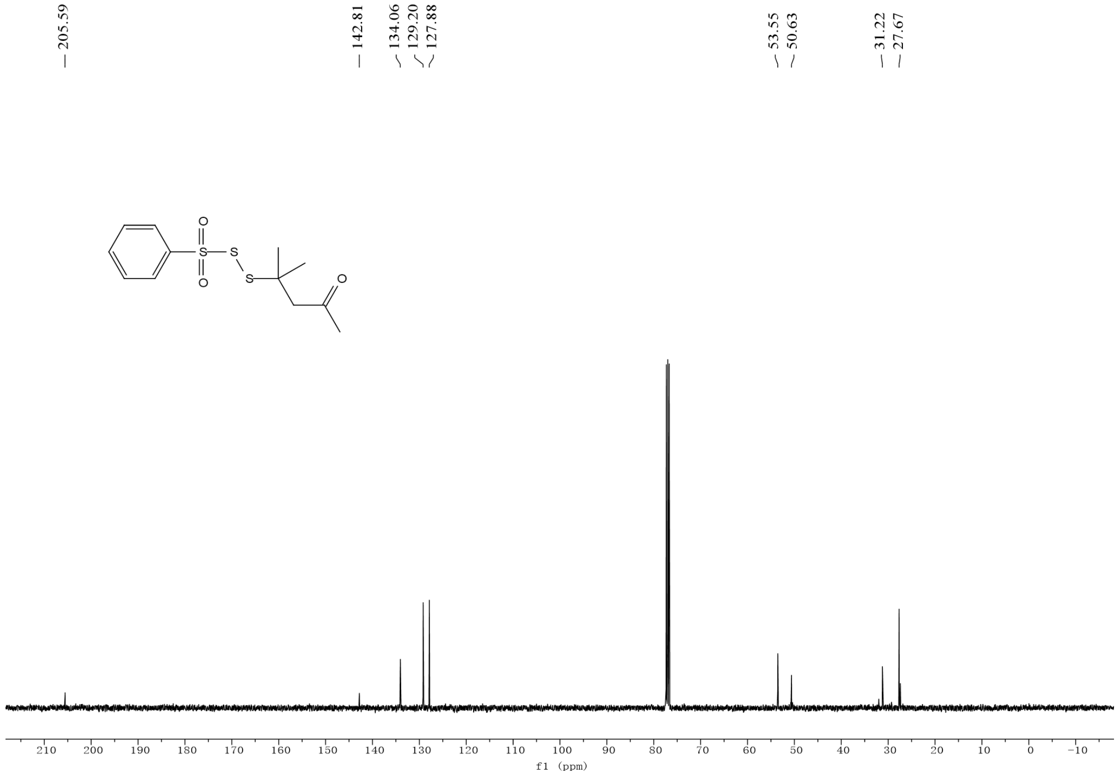

^{13}C NMR of compound 2b

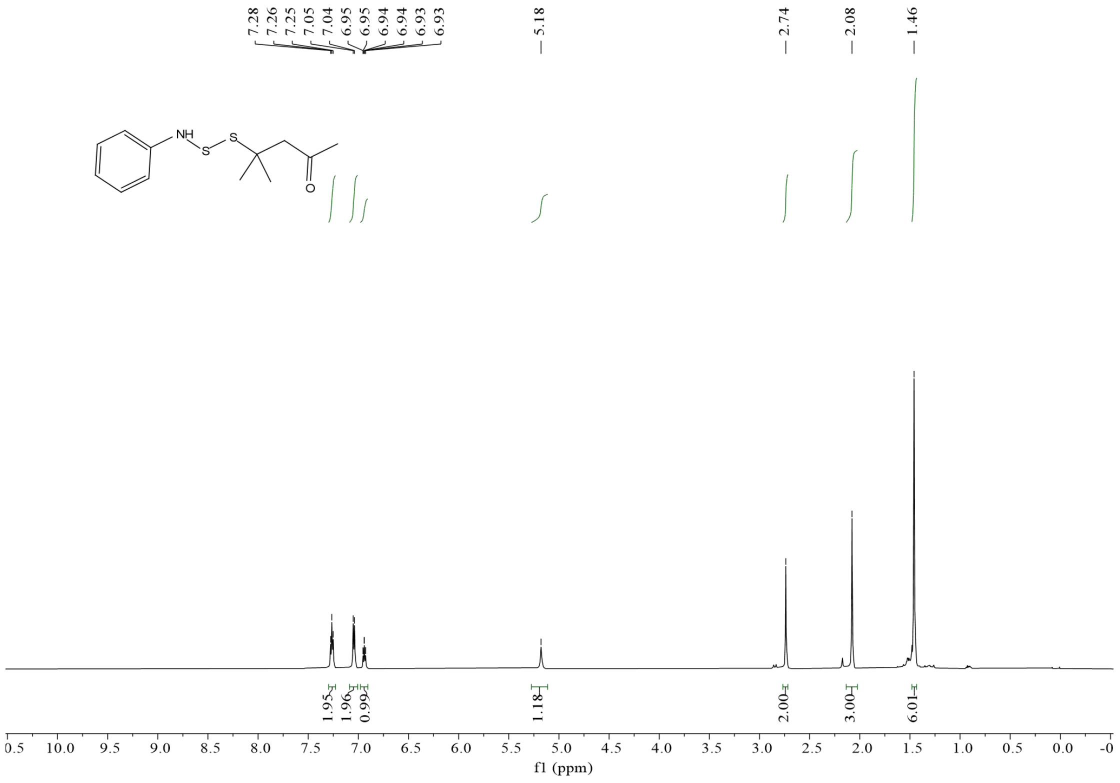

^{1}H NMR of compound 2c

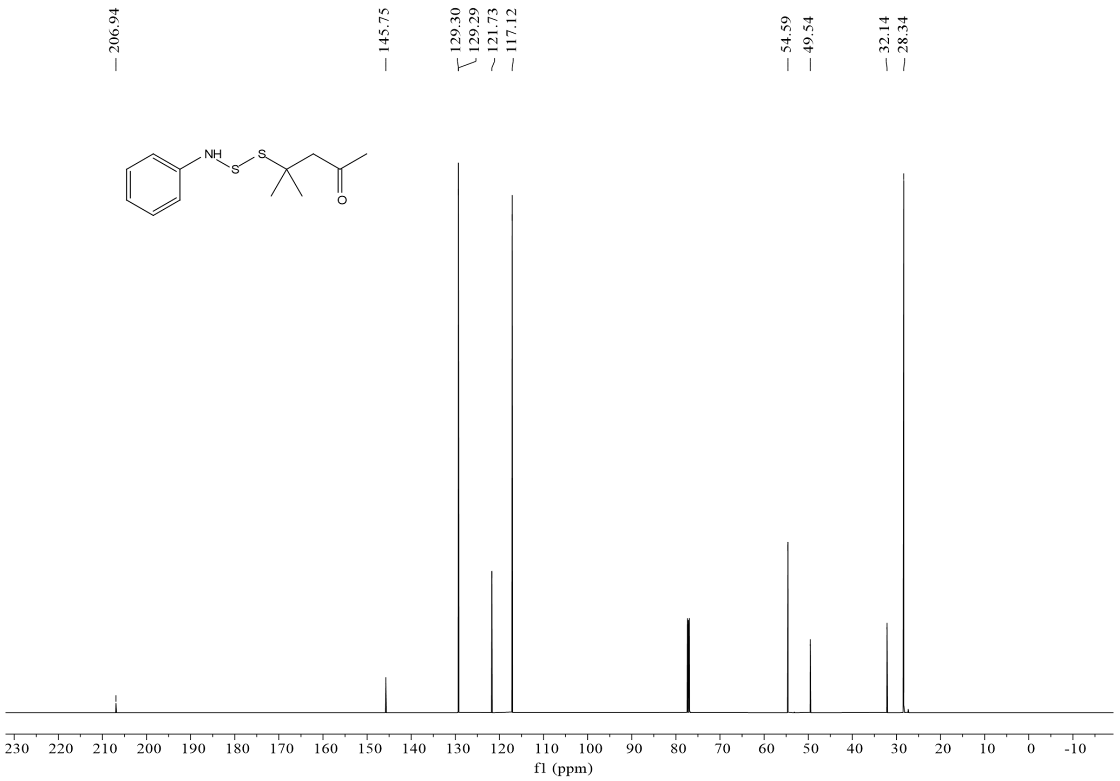

^{13}C NMR of compound 2c

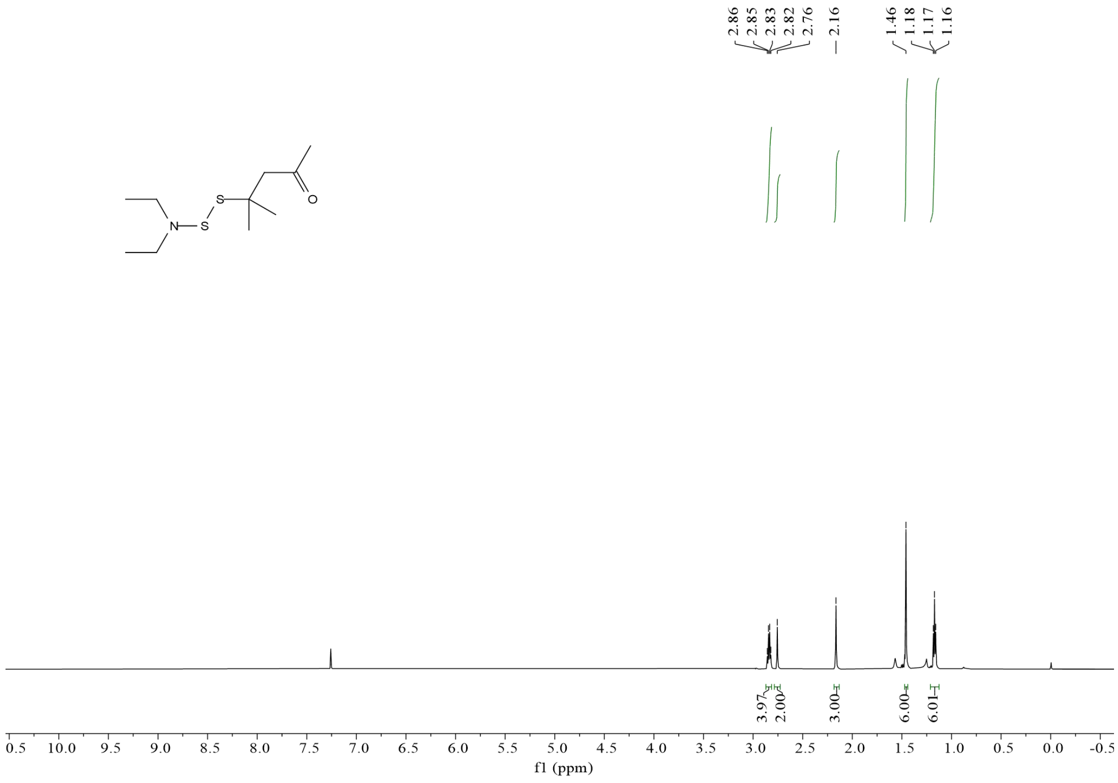

^{1}H NMR of compound 2d

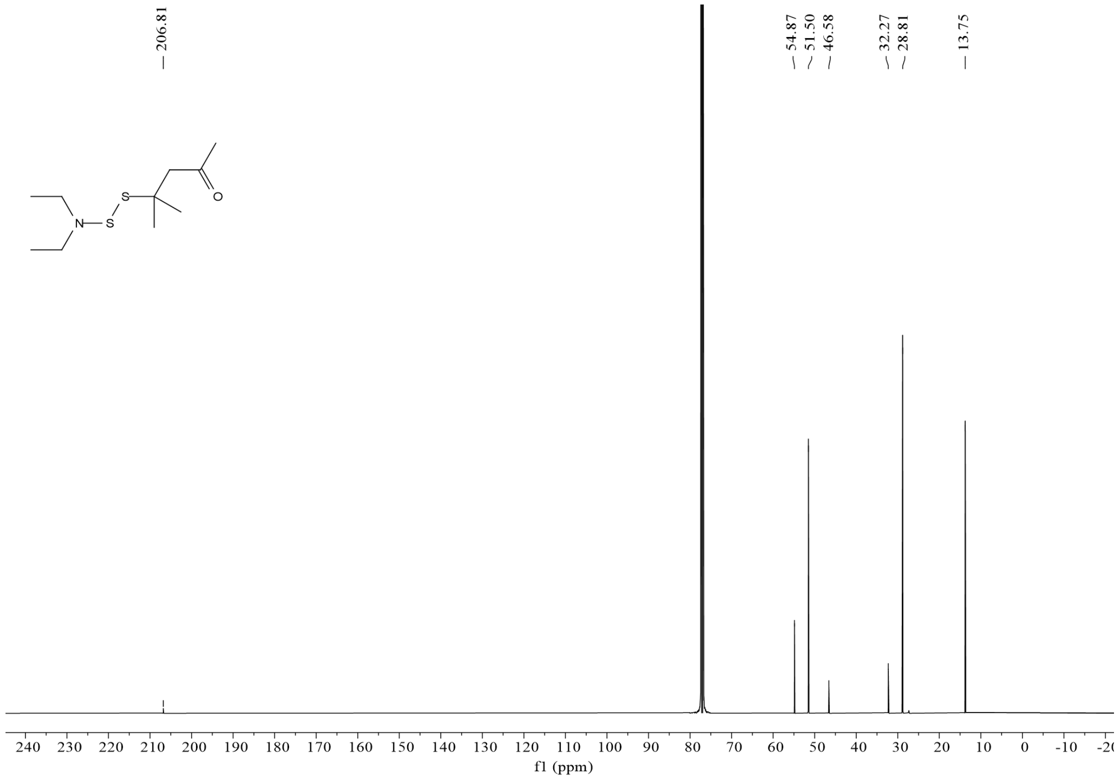

^{13}C NMR of compound 2d

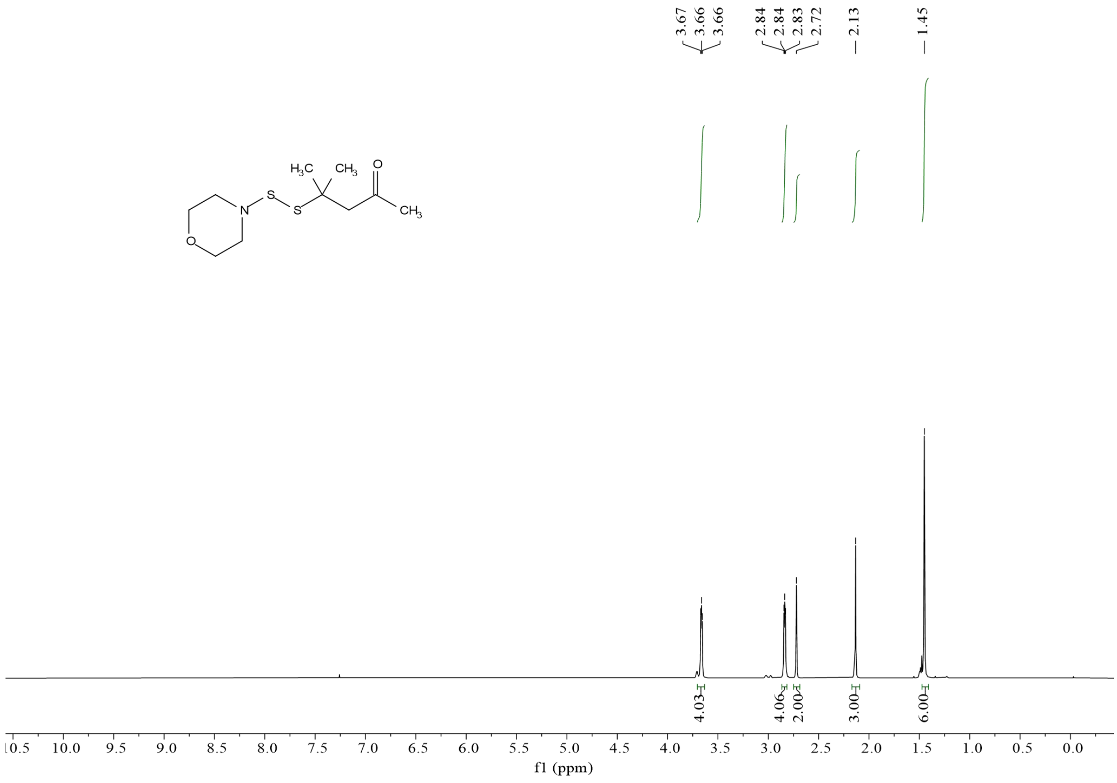

^{1}H NMR of compound 2e

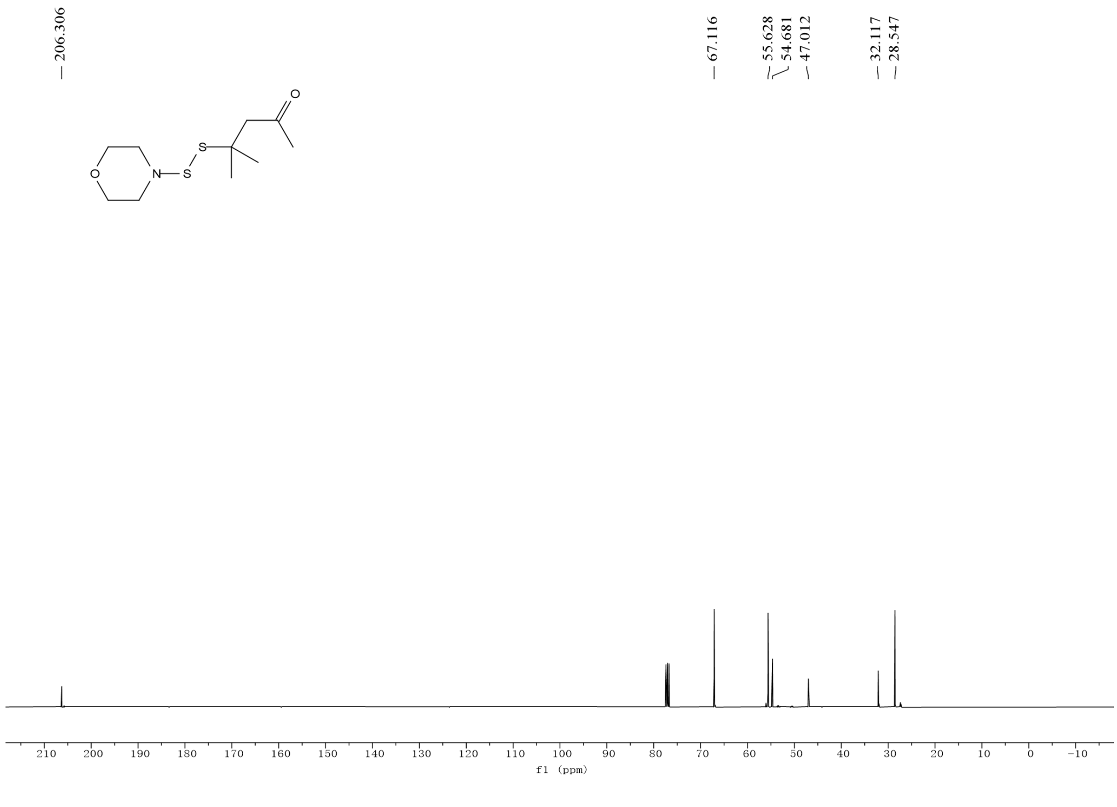

^{13}C NMR of compound 2e

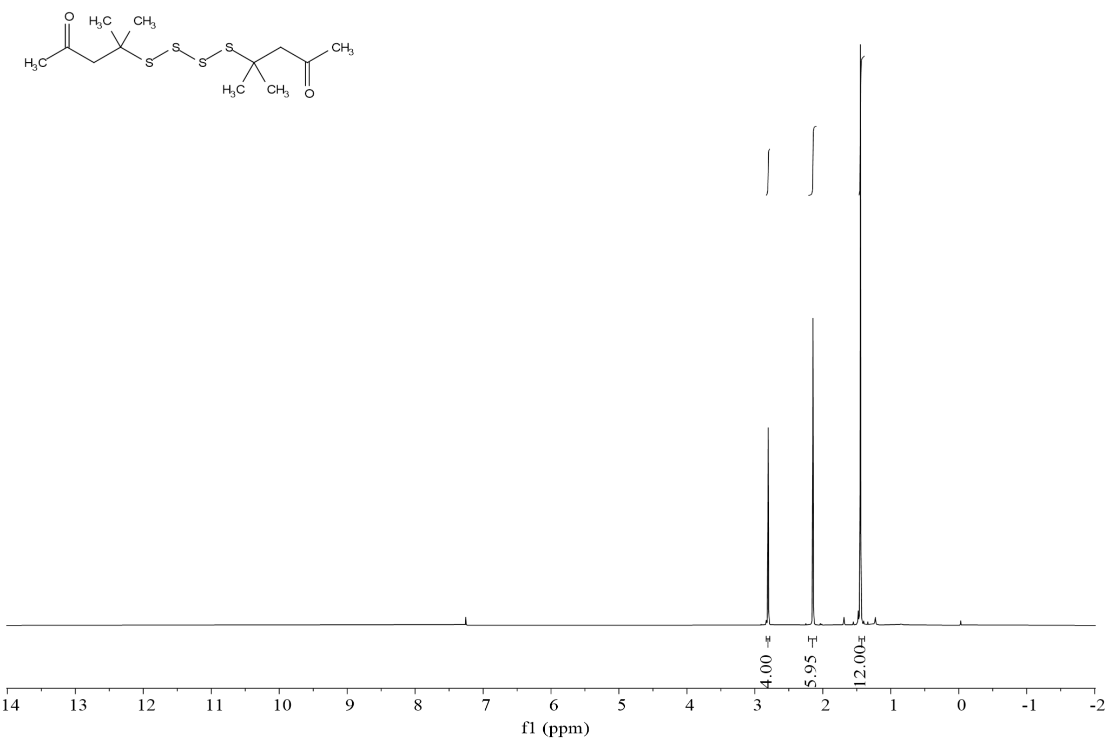

^{1}H NMR of compound 10

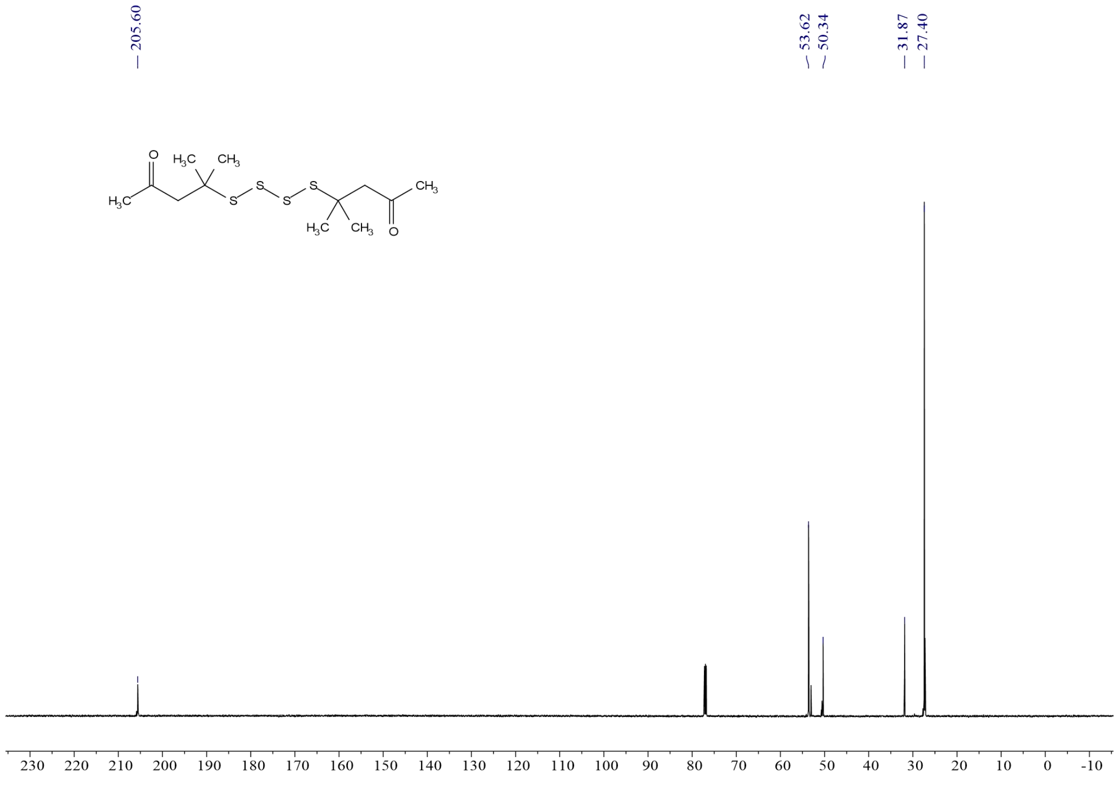

^{13}C NMR of compound 10

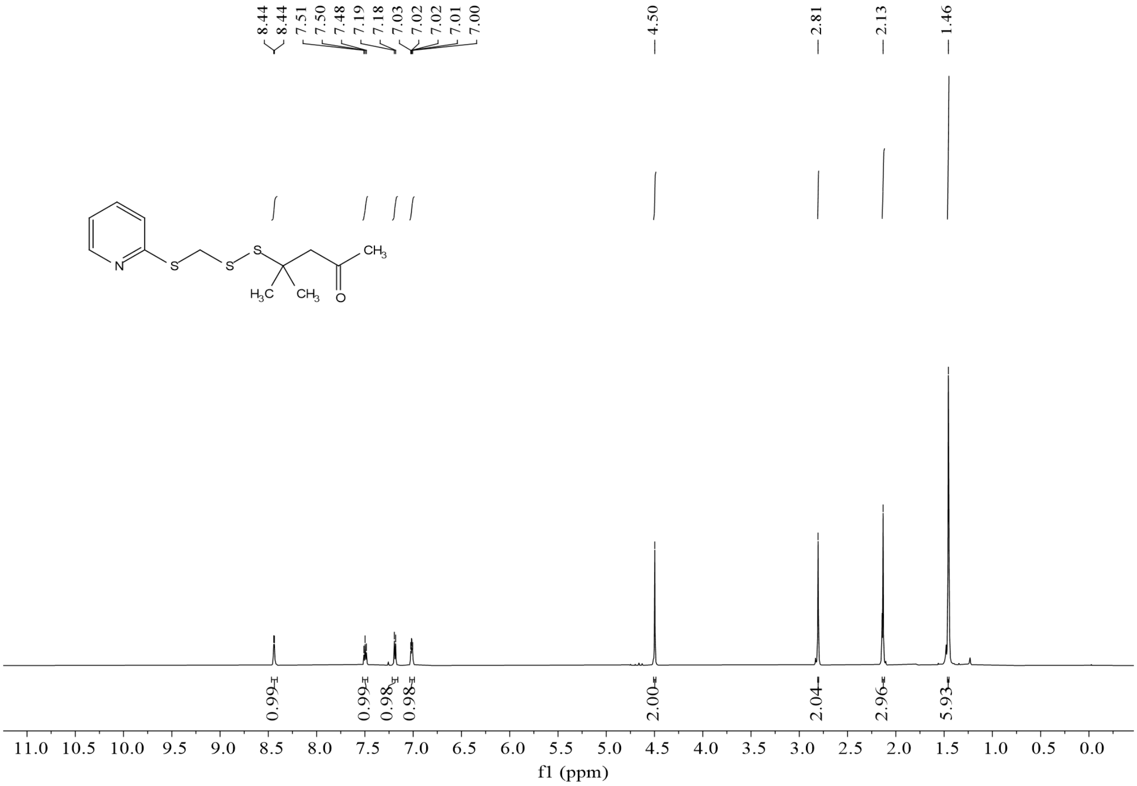

^{1}H NMR of compound 3a

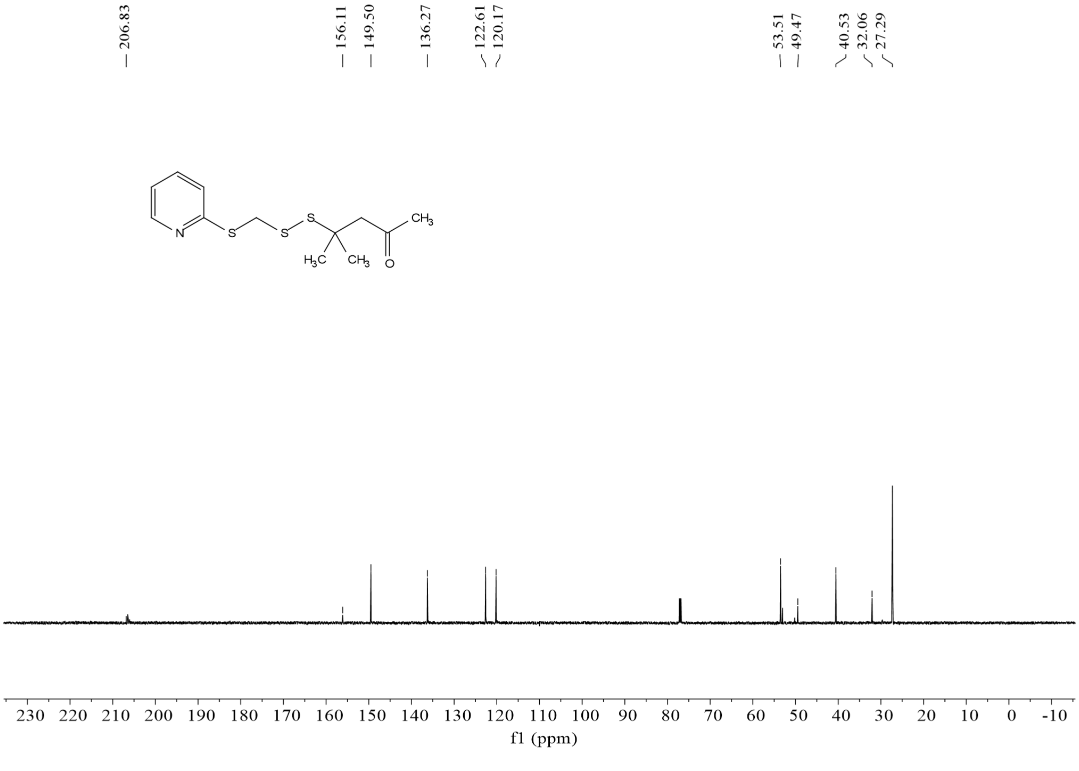

^{3}C NMR of compound 3a

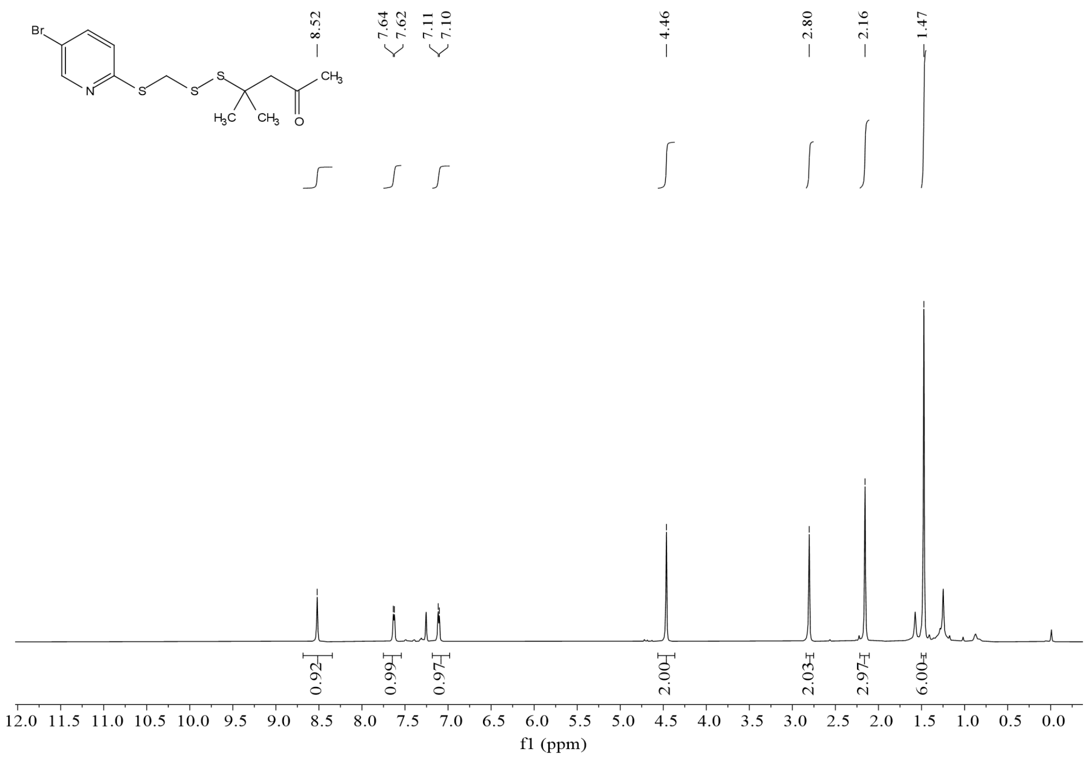

^{1}H NMR of compound 3b

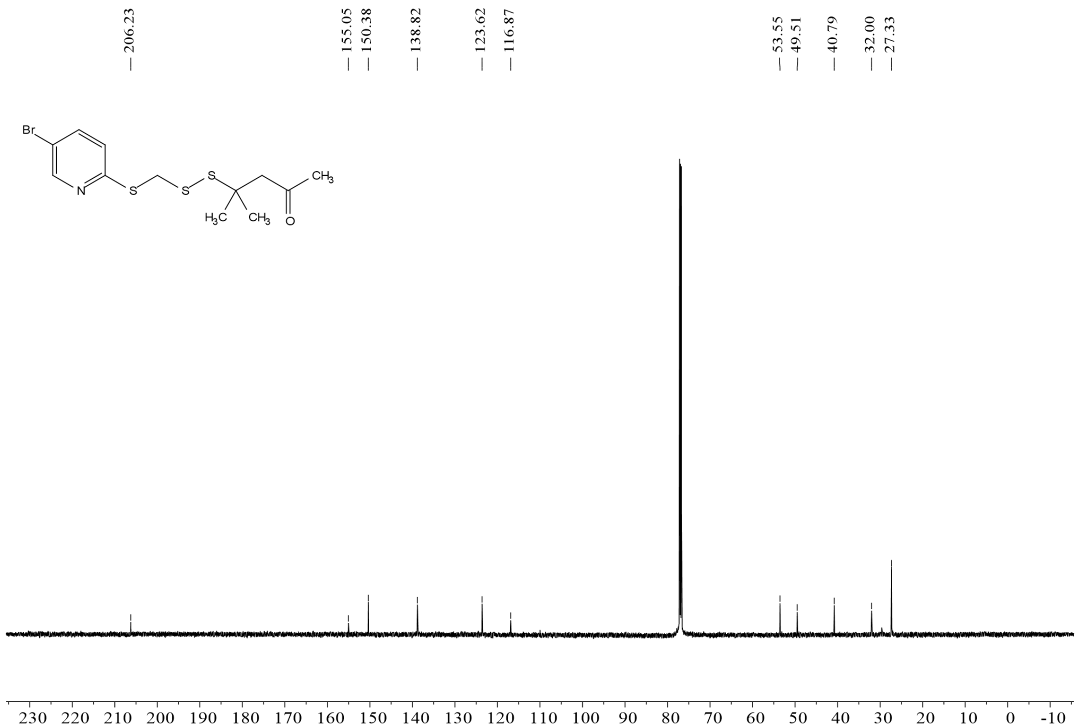

^{13}C NMR of compound 3b

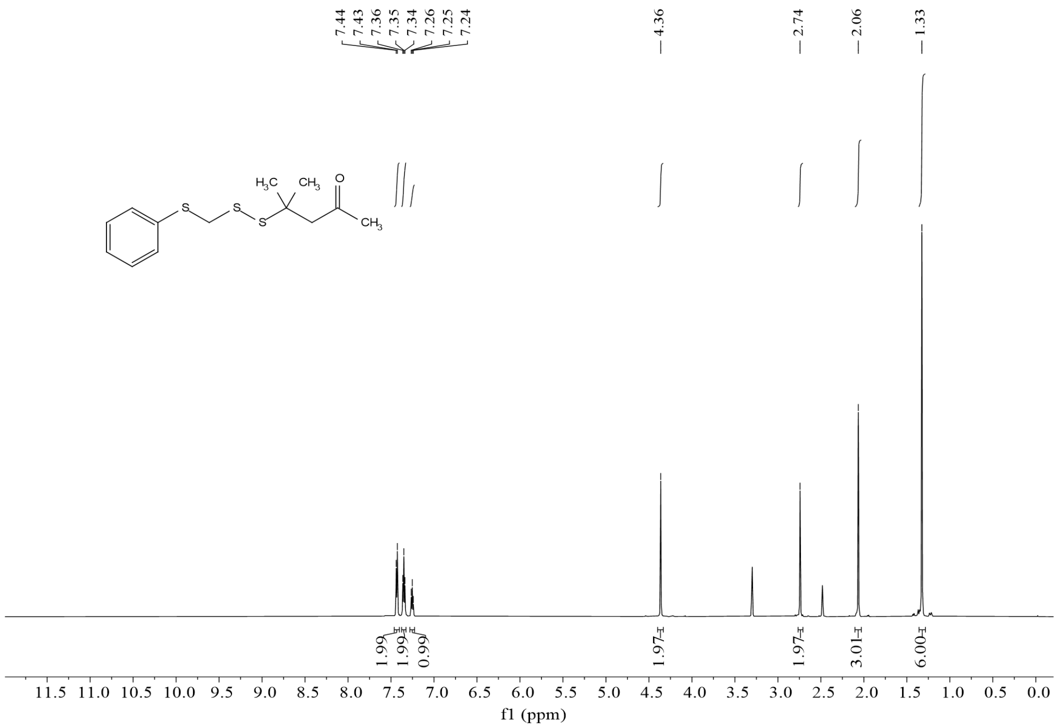

^{1}H NMR of compound 3c

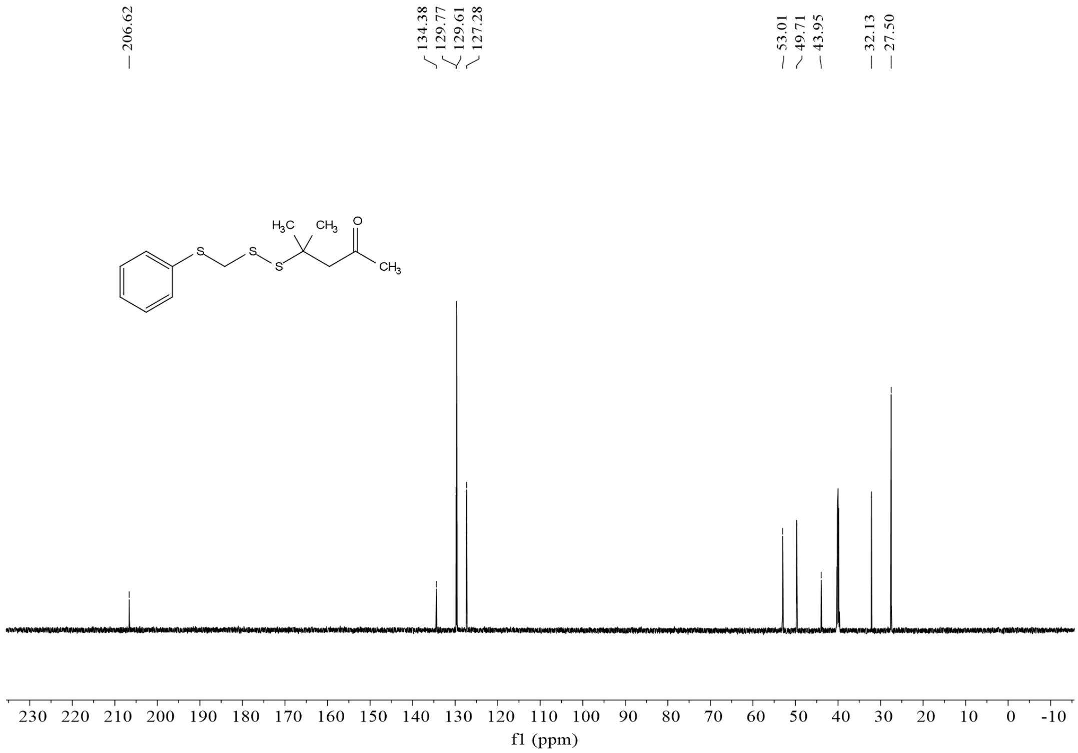

^{13}C NMR of compound 3c

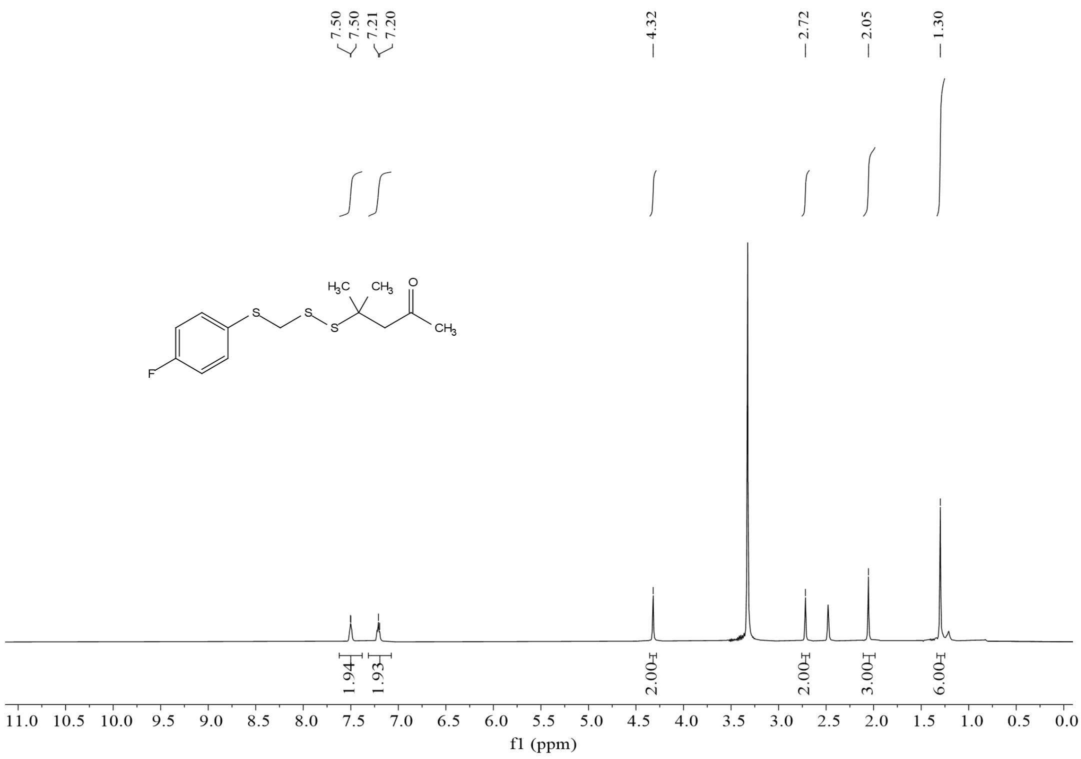

^{1}H NMR of compound 3d

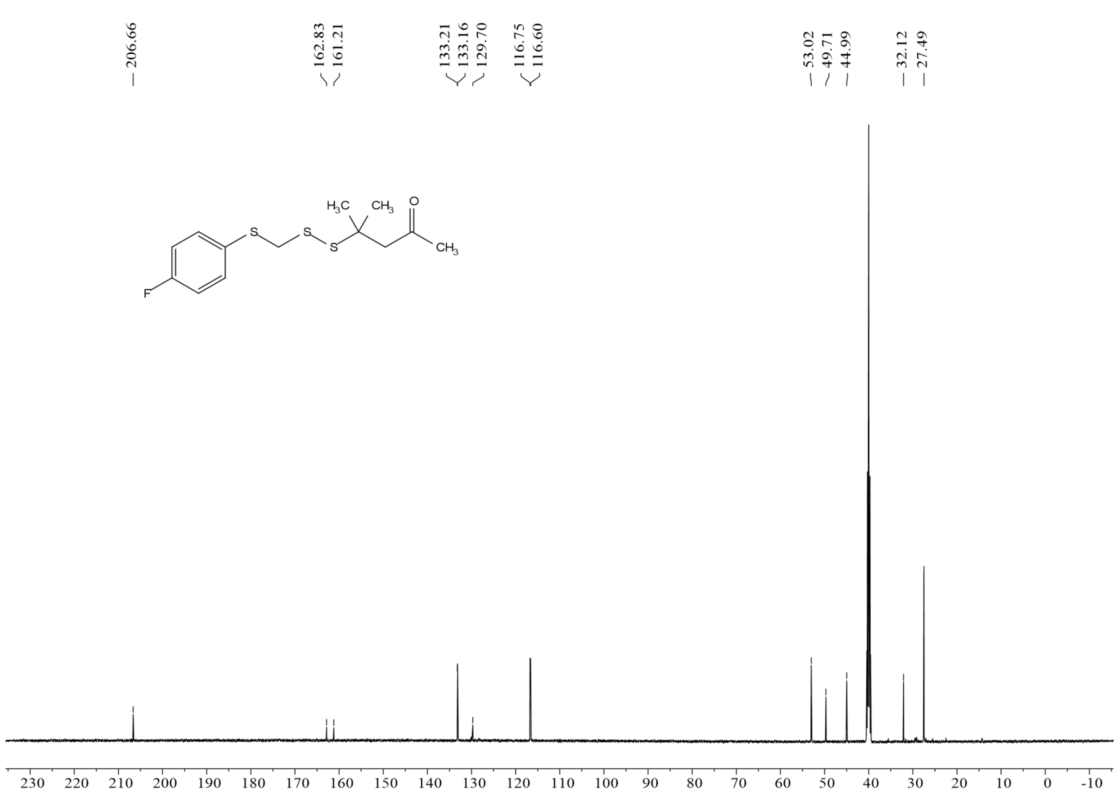

^{13}C NMR of compound 3d

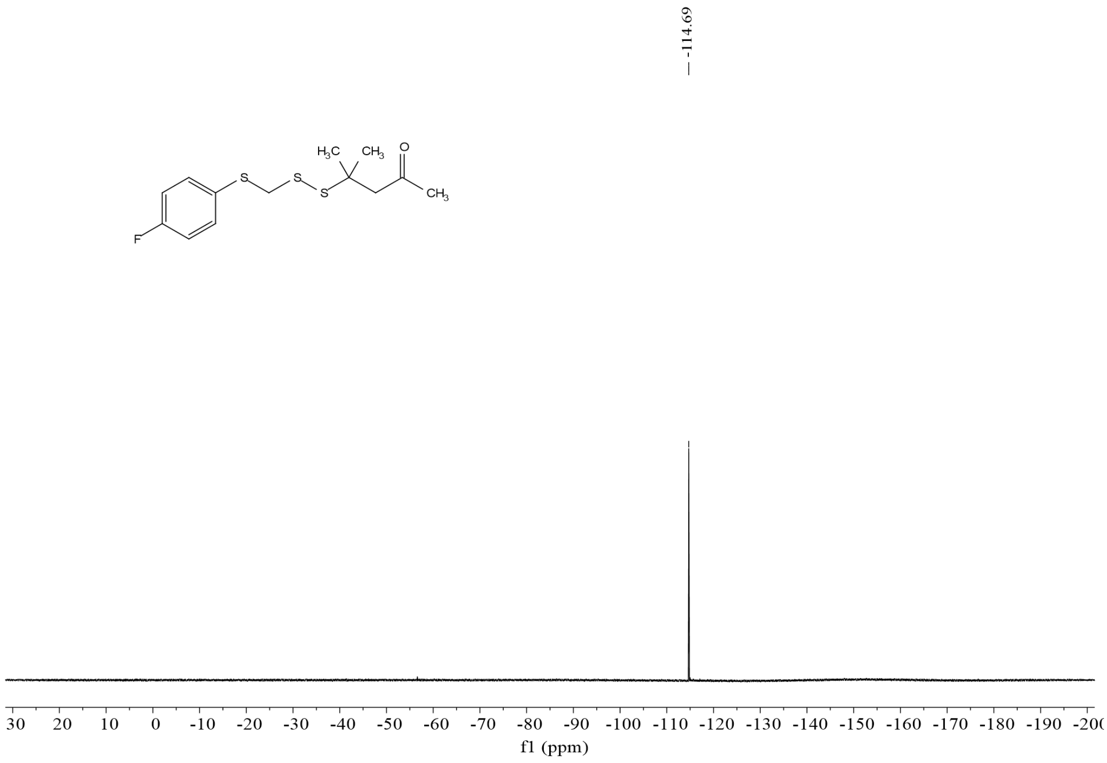

^{19}F NMR of compound 3d

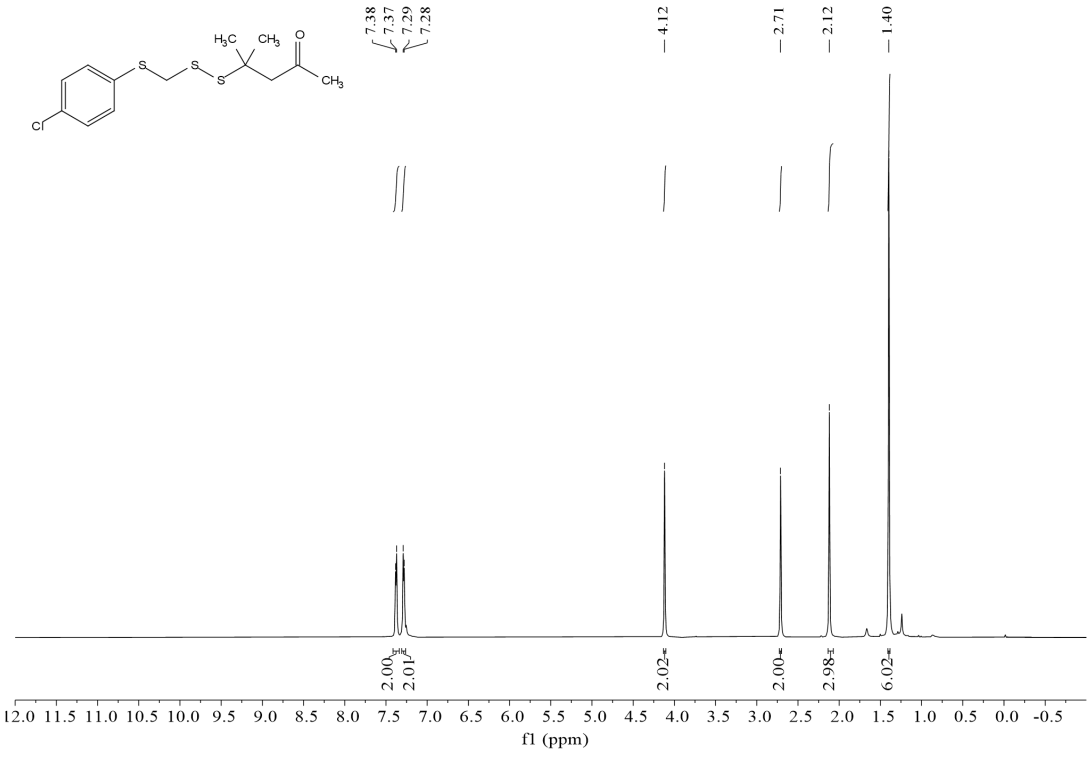

^{1}H NMR of compound 3e

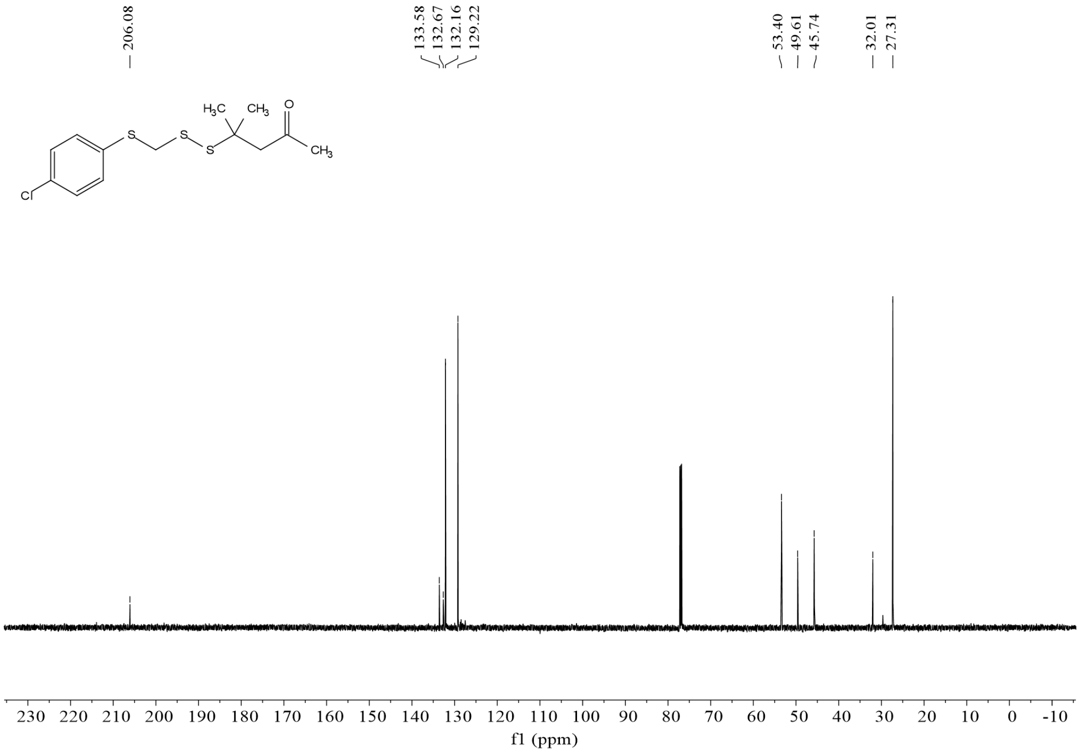

^{13}C NMR of compound 3e

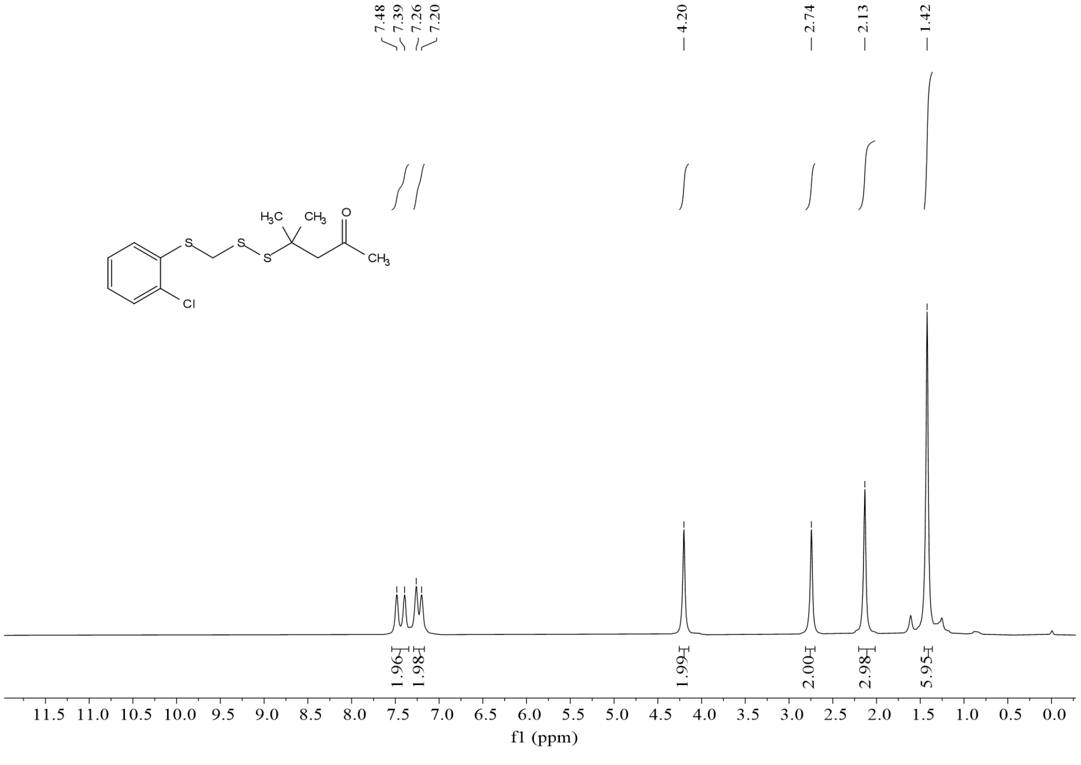

^{1}H NMR of compound 3f

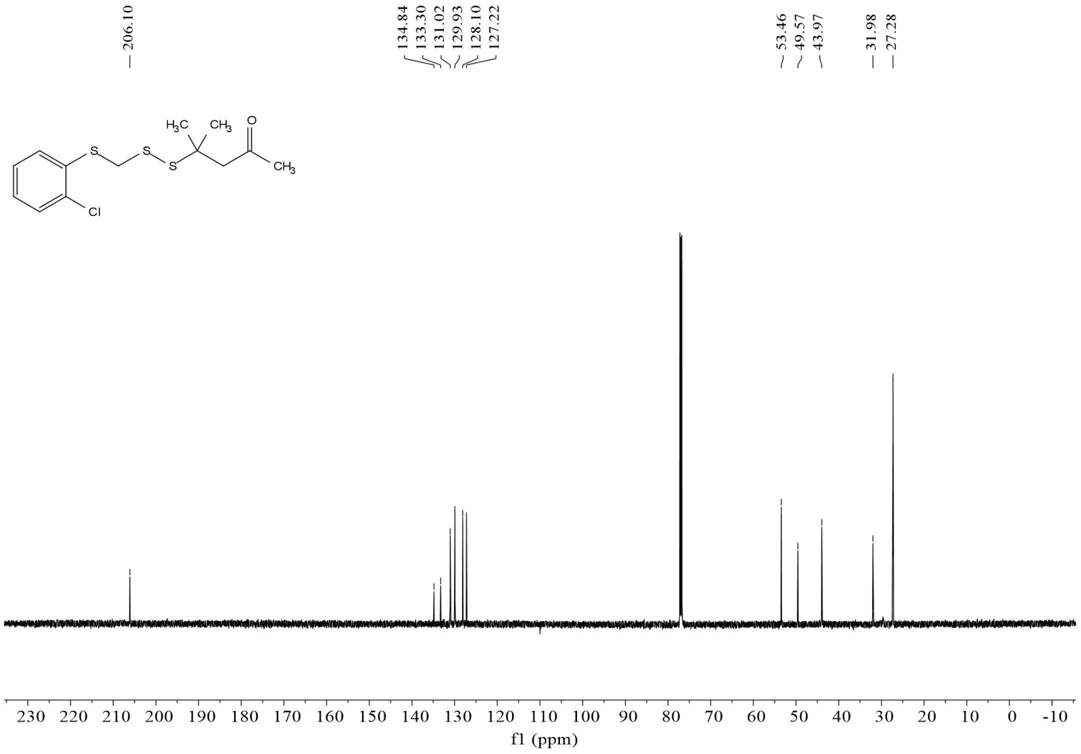

^{13}C NMR of compound 3f

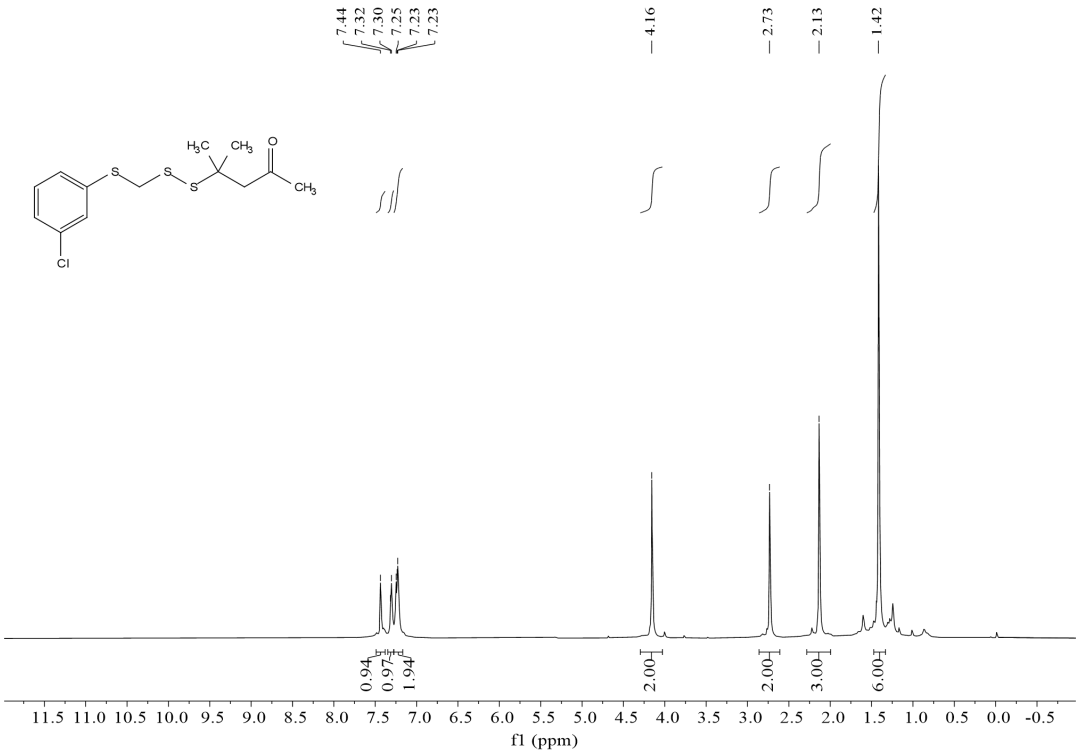

^{1}H NMR of compound 3g

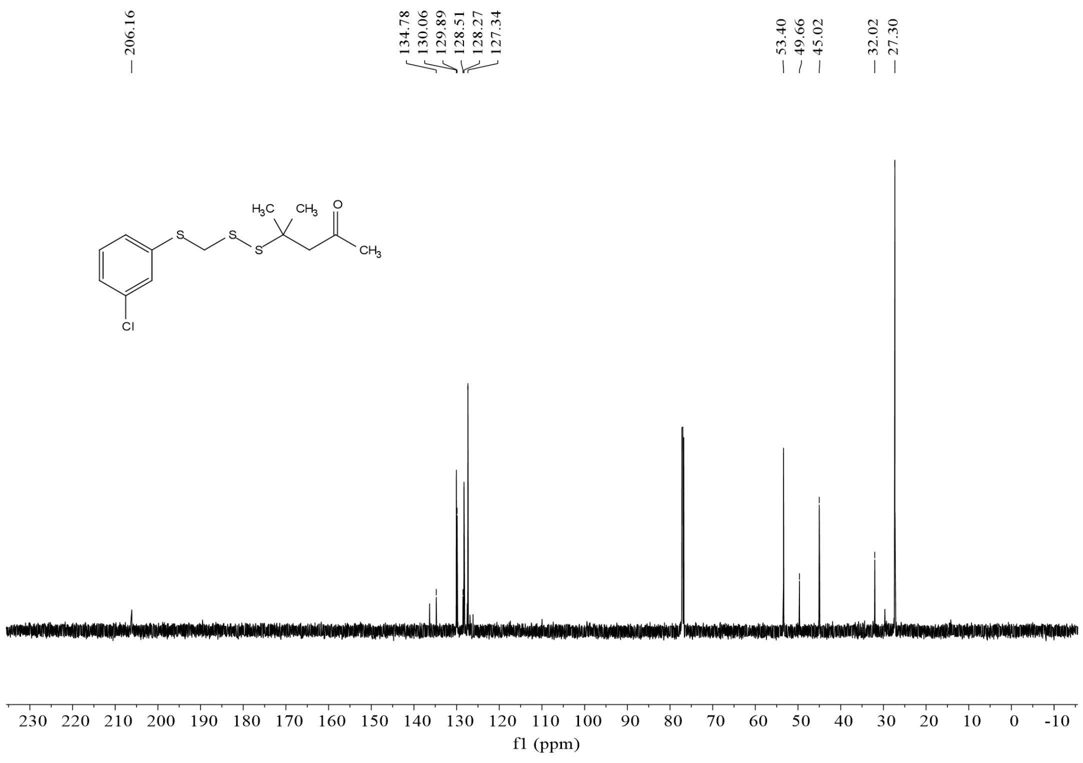

^{13}C NMR of compound 3g

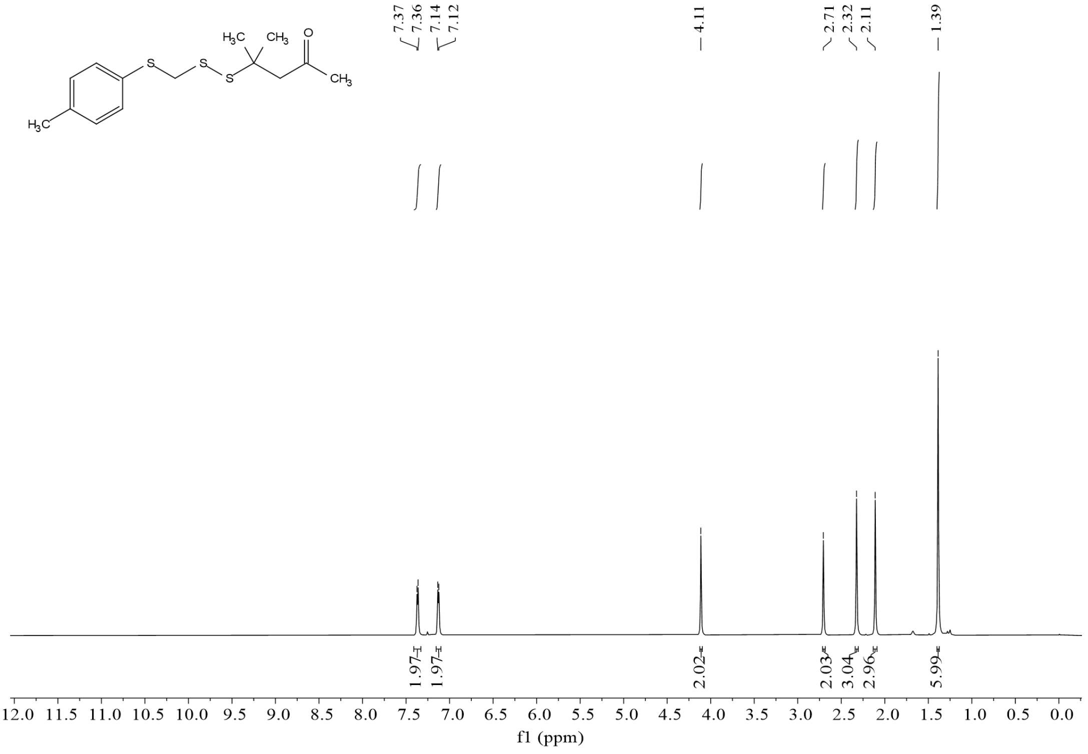

H NMR of compound 3h

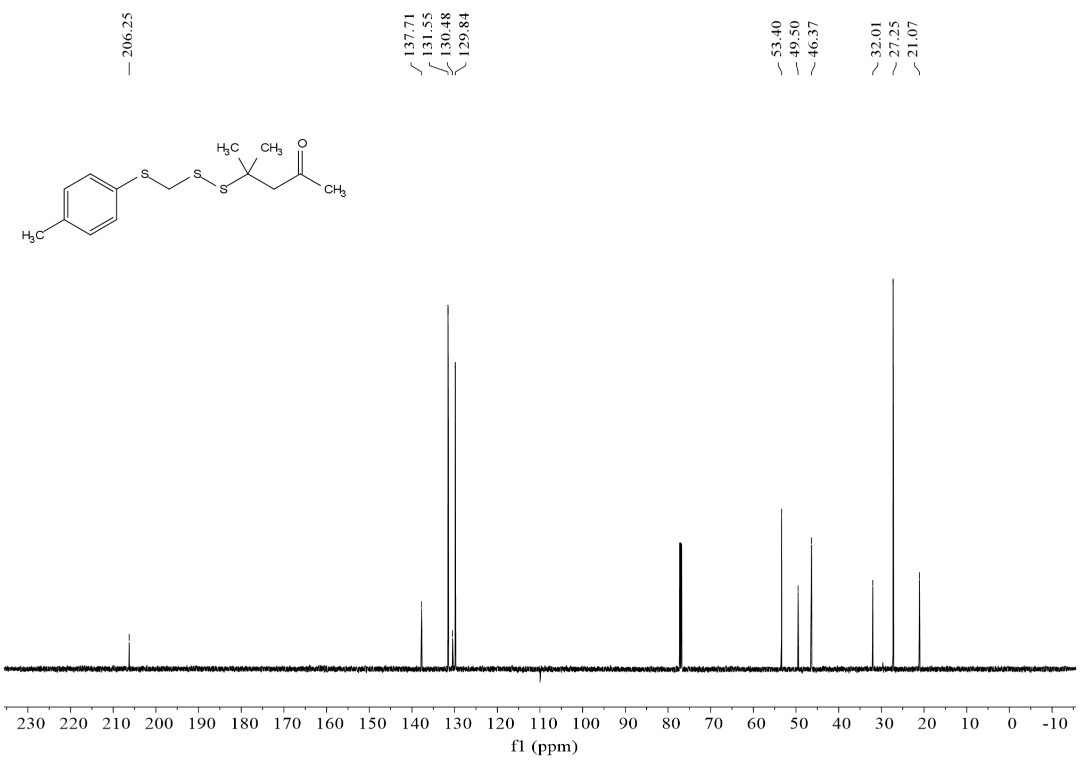

1

^{3}C NMR of compound 3h

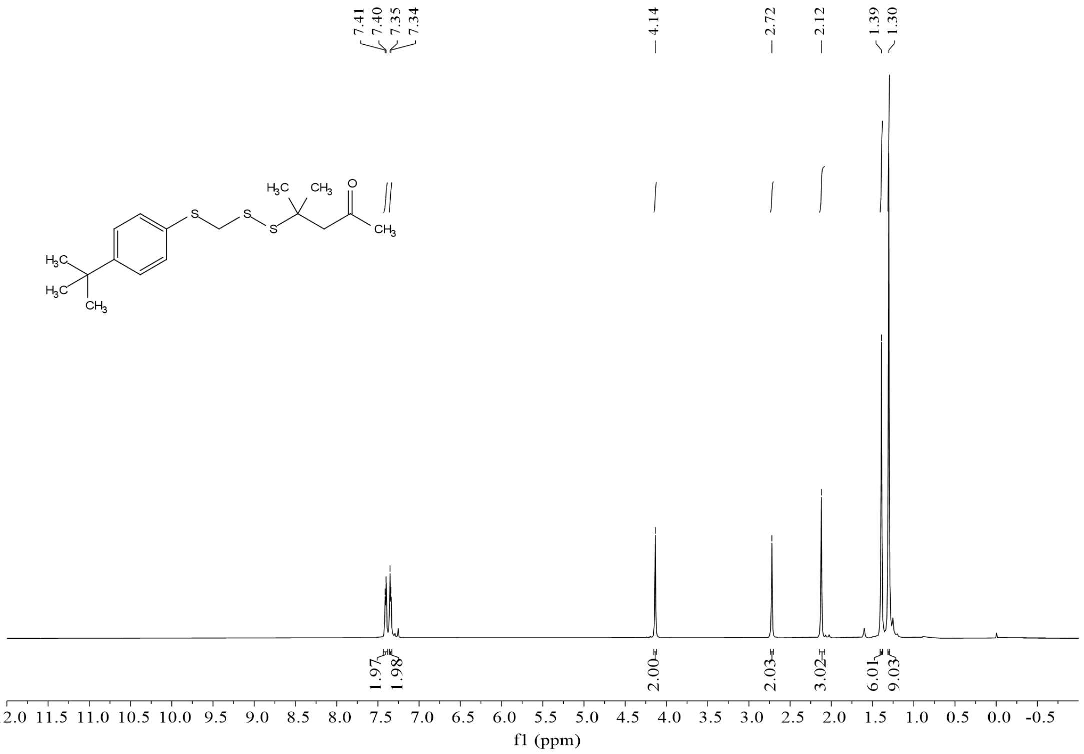

^{1}H NMR of compound 3i

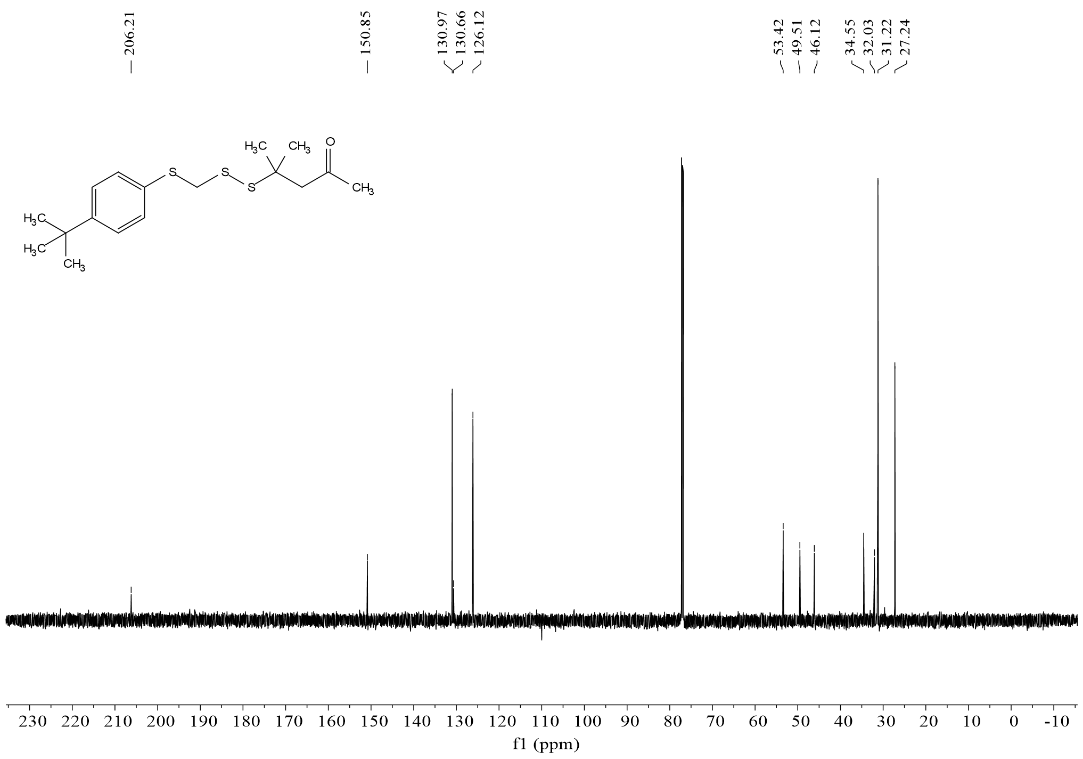

^{13}C NMR of compound 3i

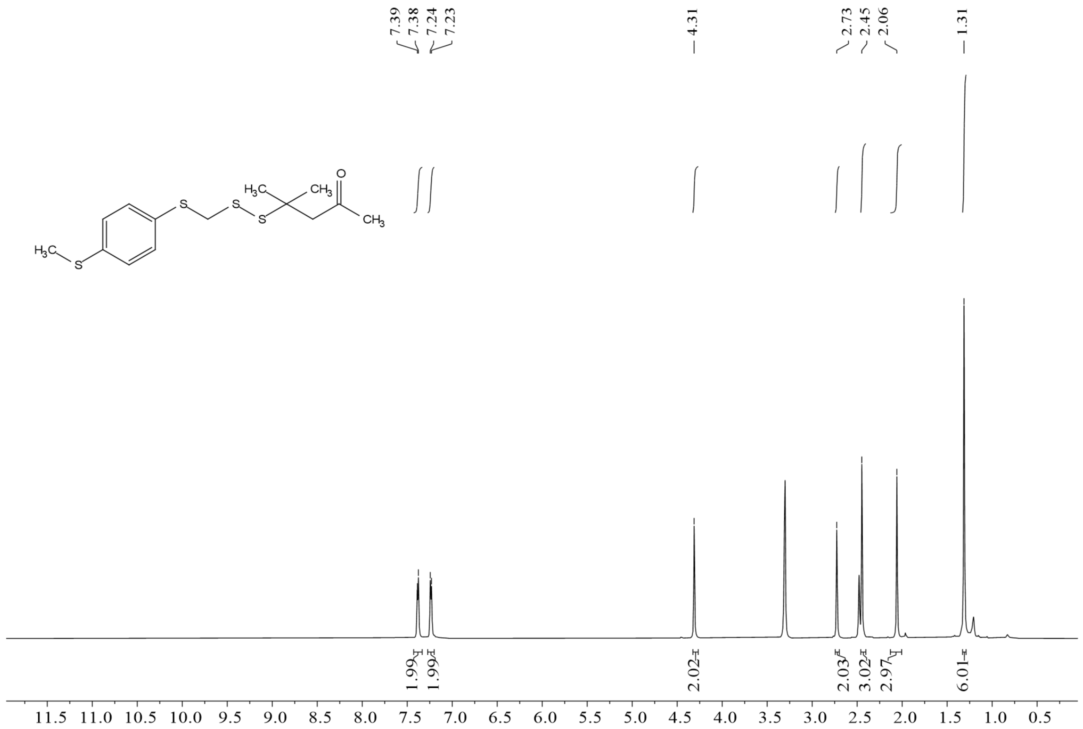

^{1}H NMR of compound 3j

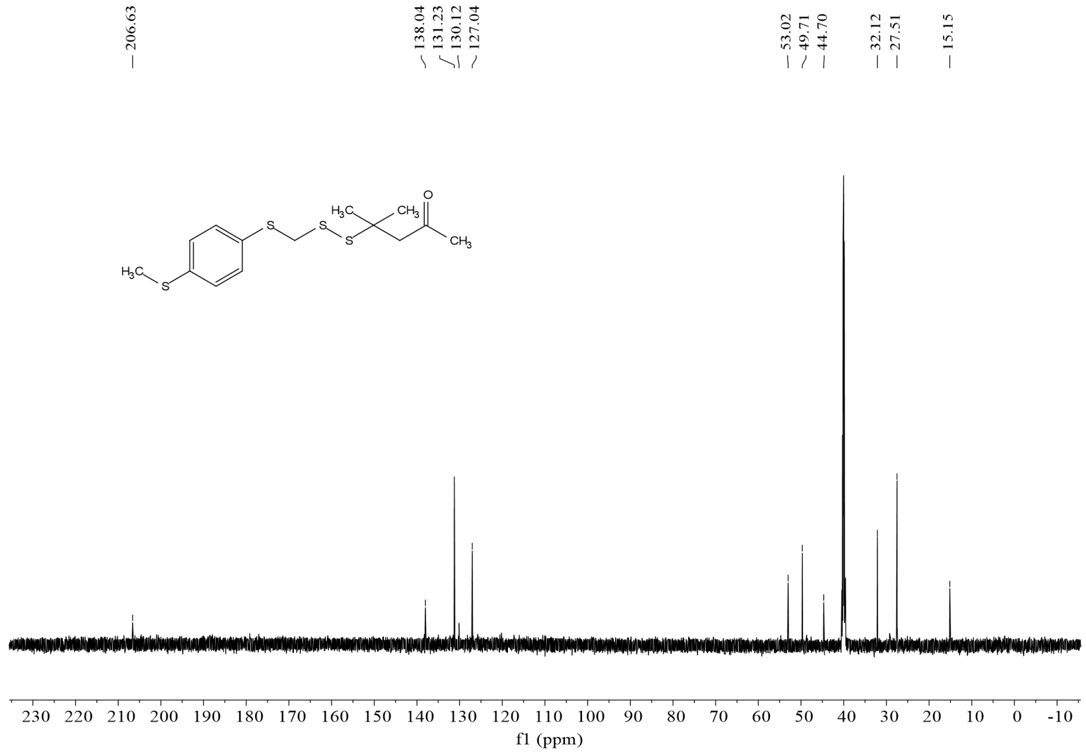

^{13}C NMR of compound 3j

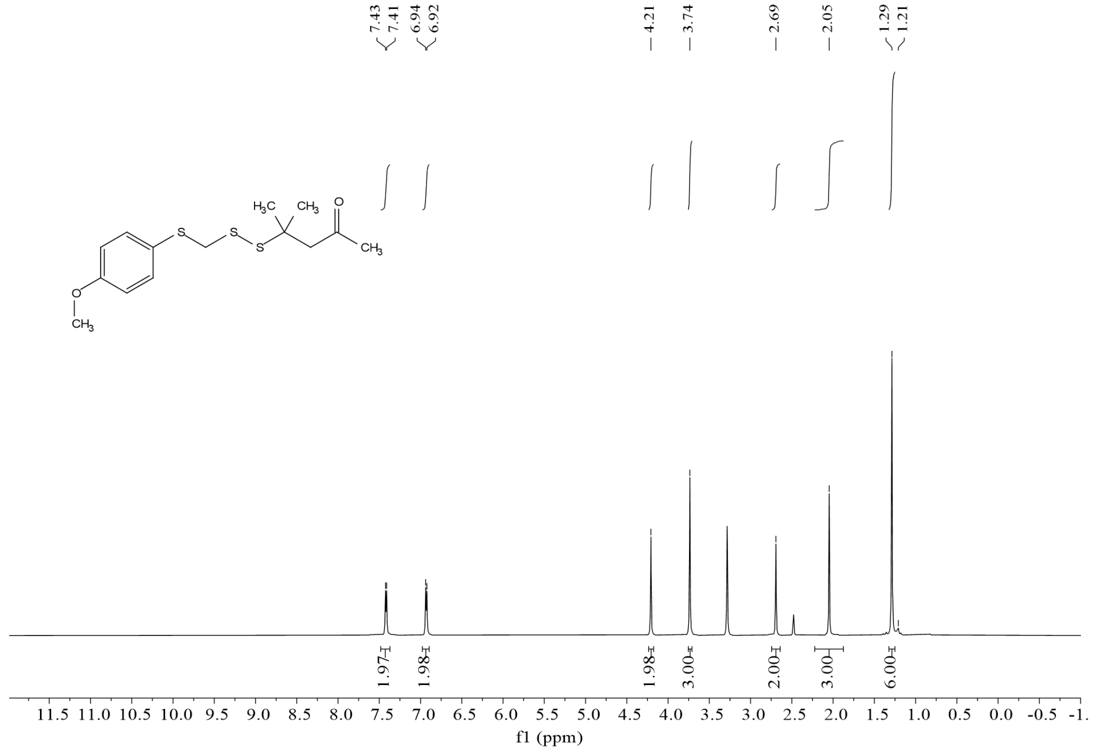

^{1}H NMR of compound 3k

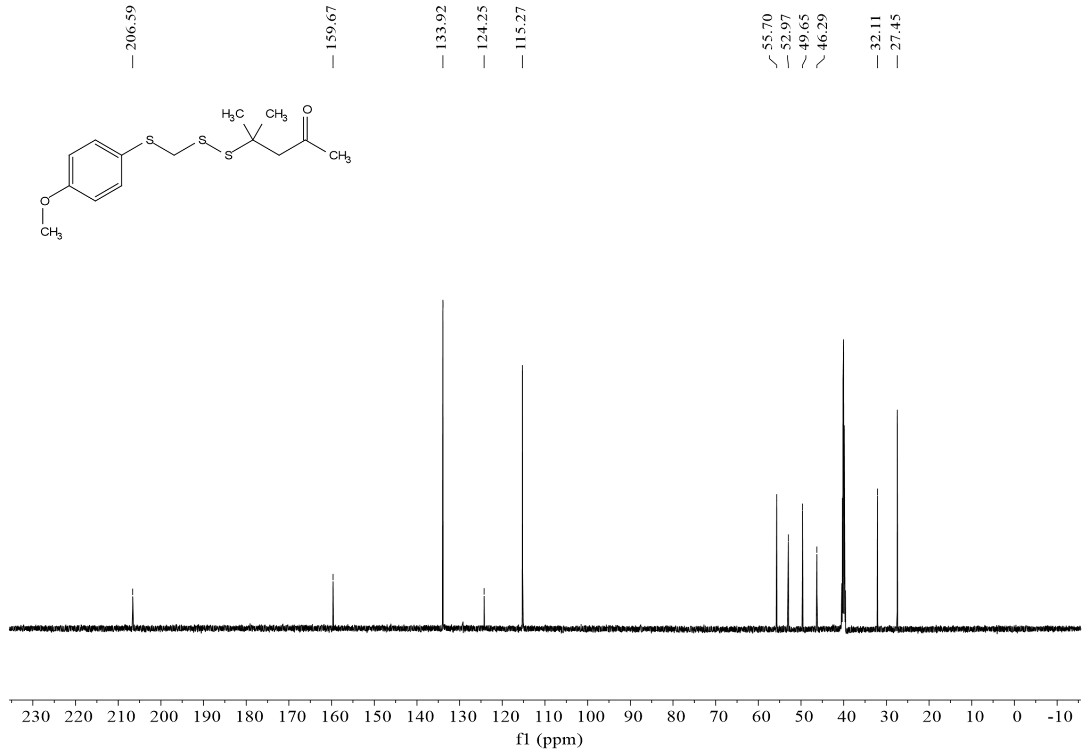

^{13}C NMR of compound 3k

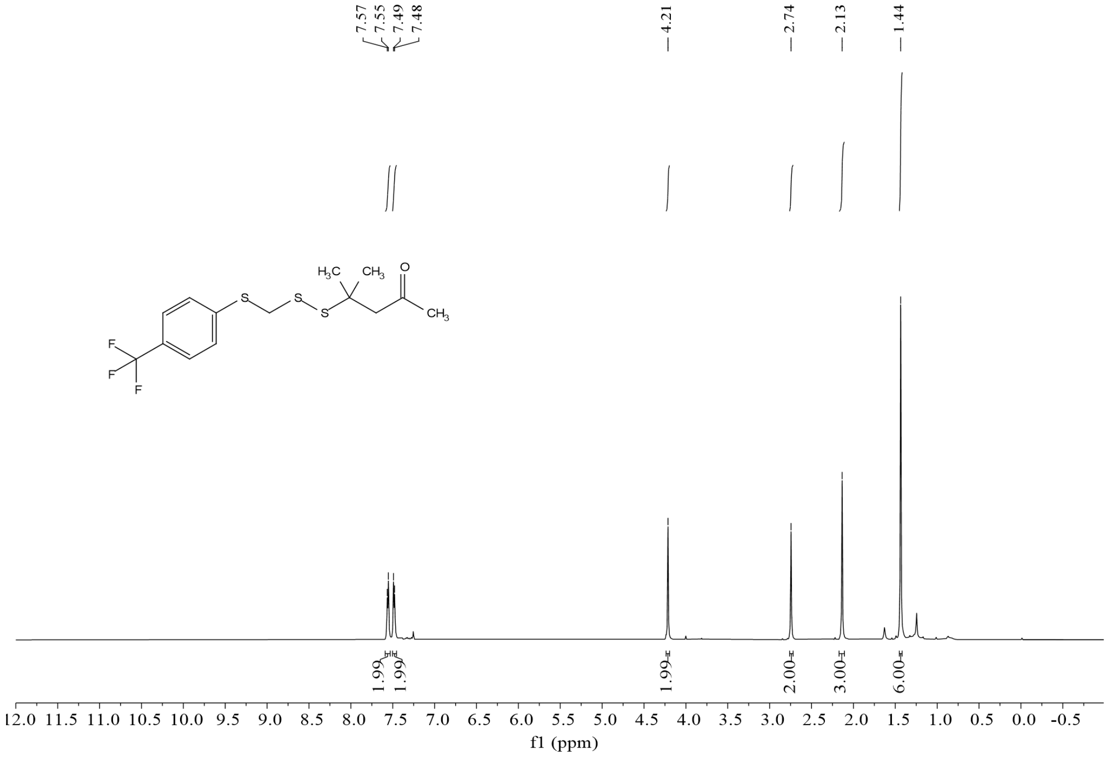

^{1}H NMR of compound 3l

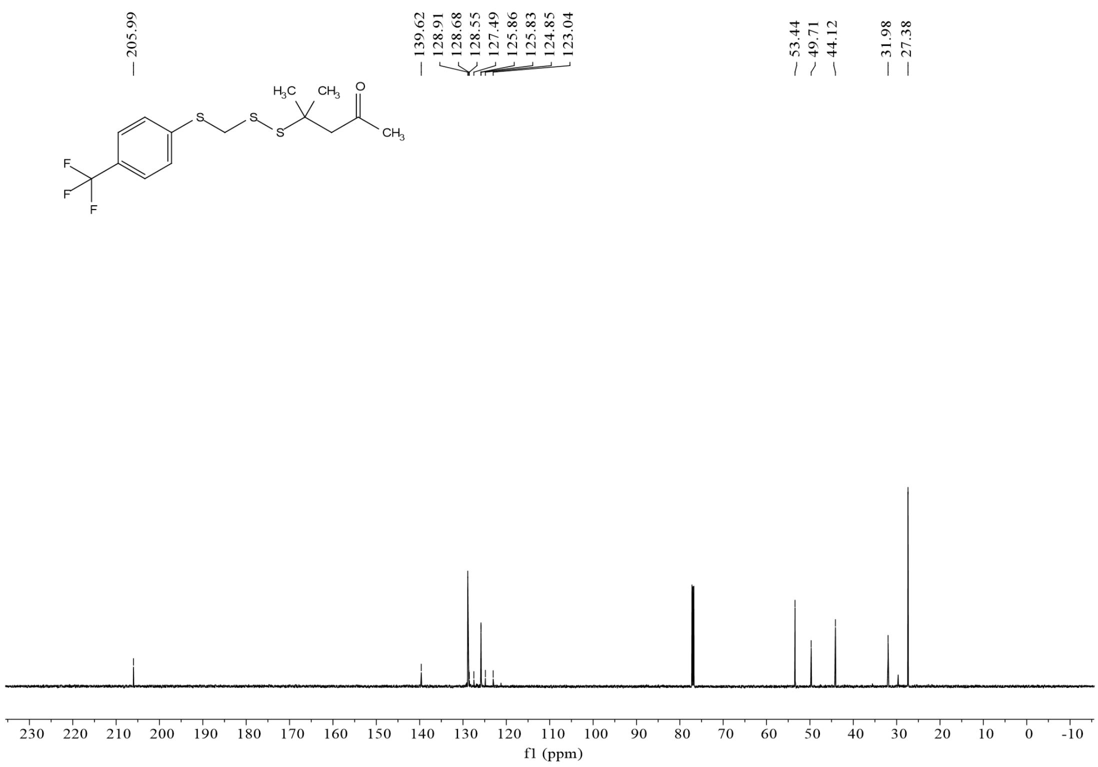

^{13}C NMR of compound 3l

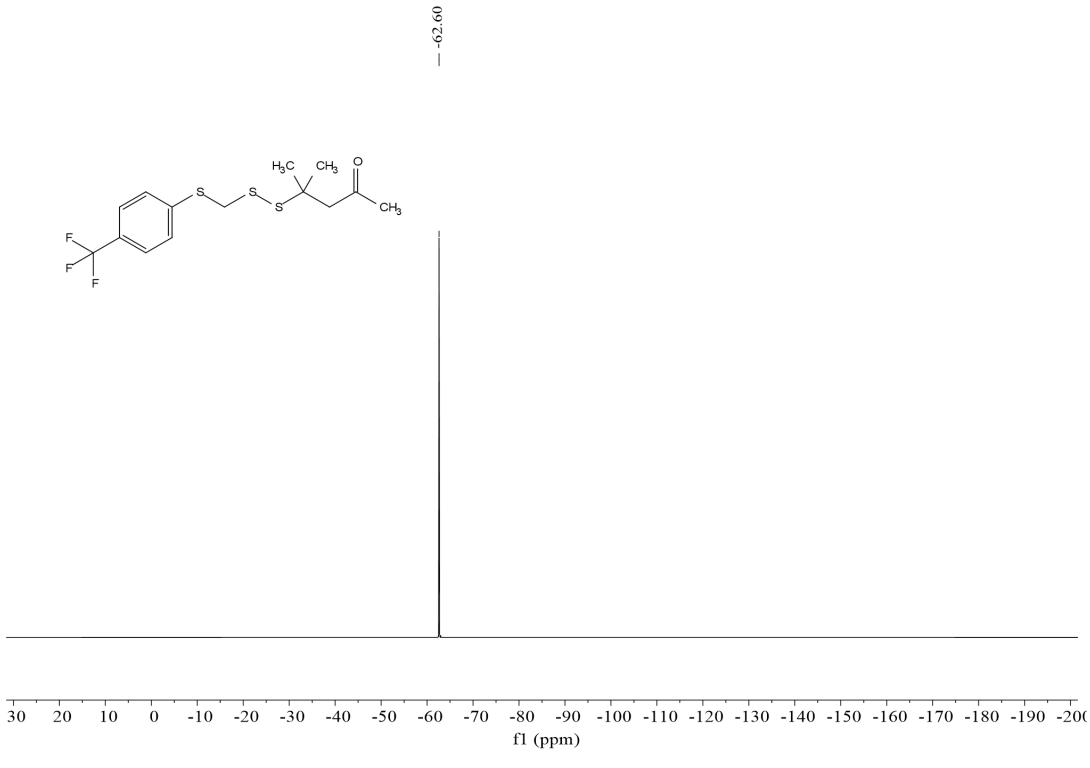

^{19}F NMR of compound 3l

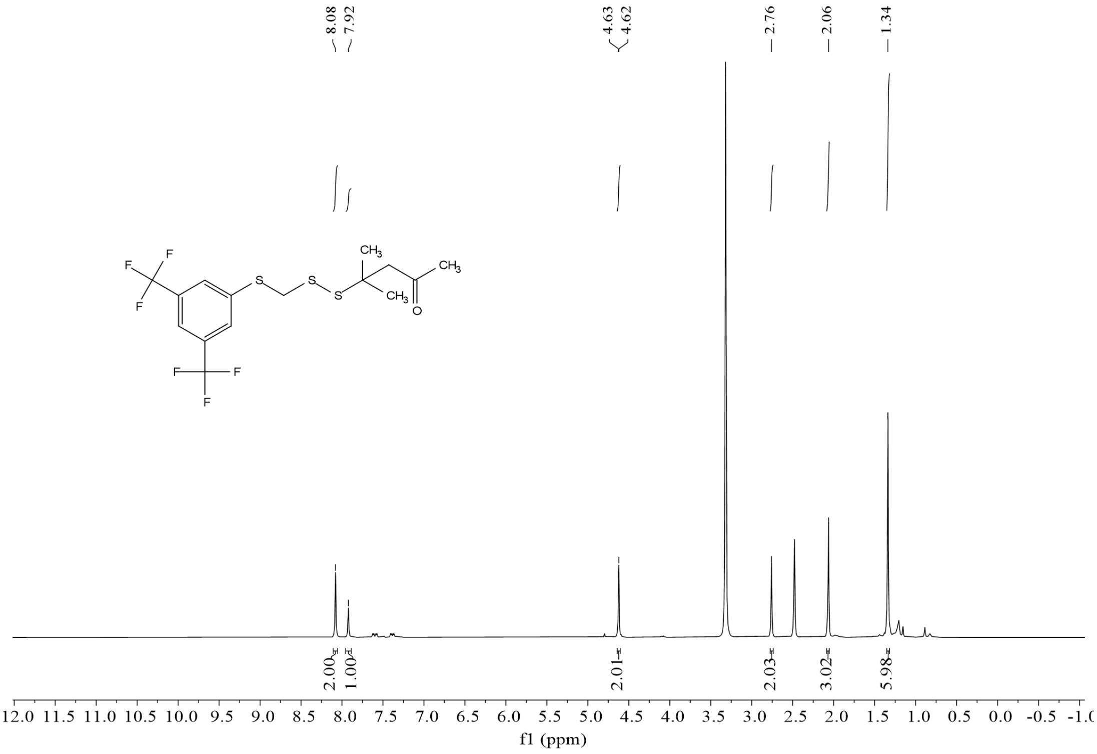

^{1}H NMR of compound 3m

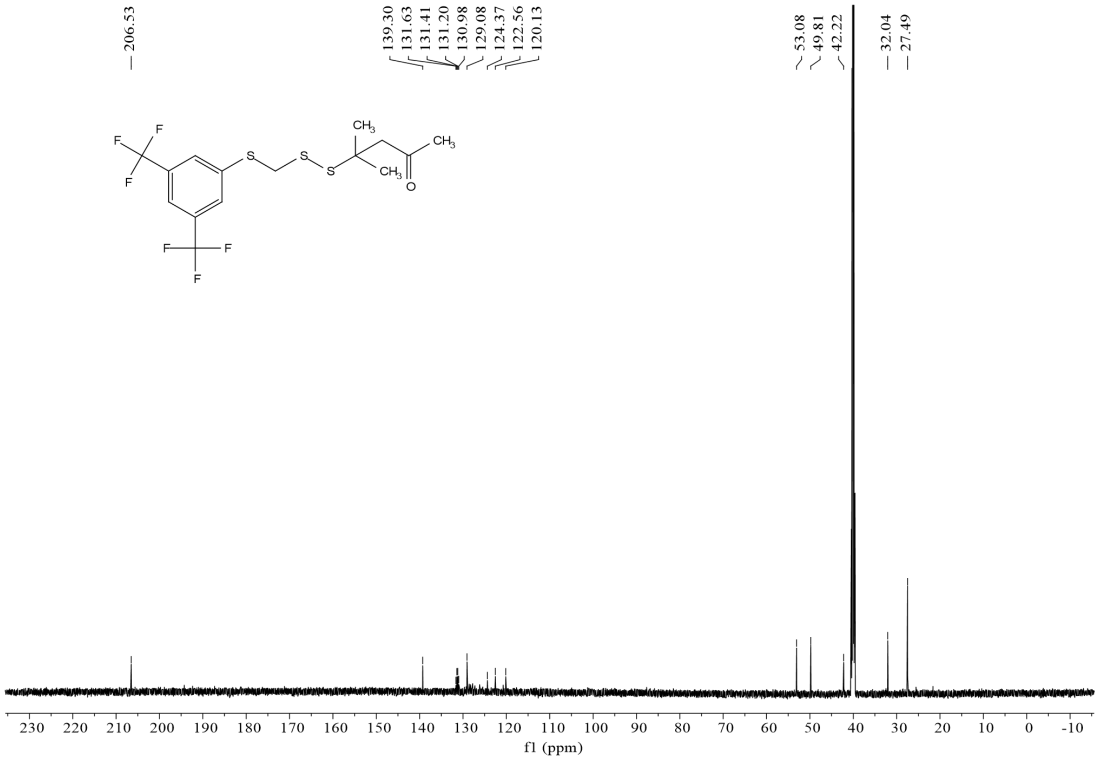

^{13}C NMR of compound 3m

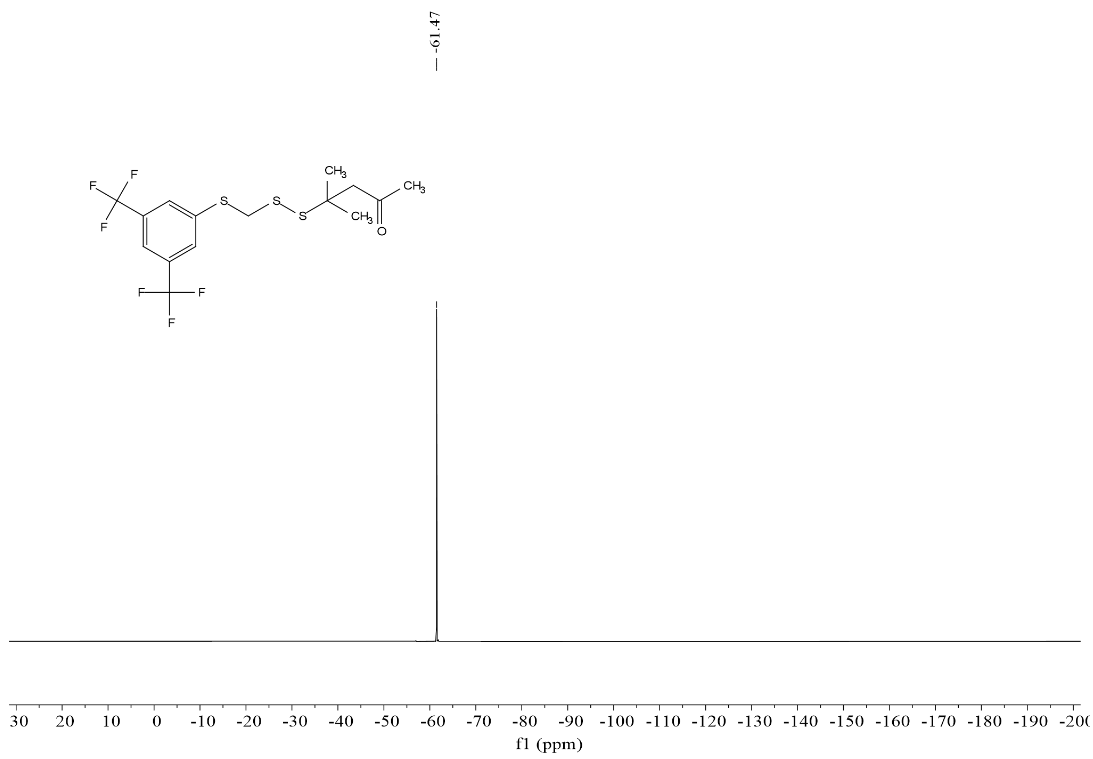

^{19}F NMR of compound 3m

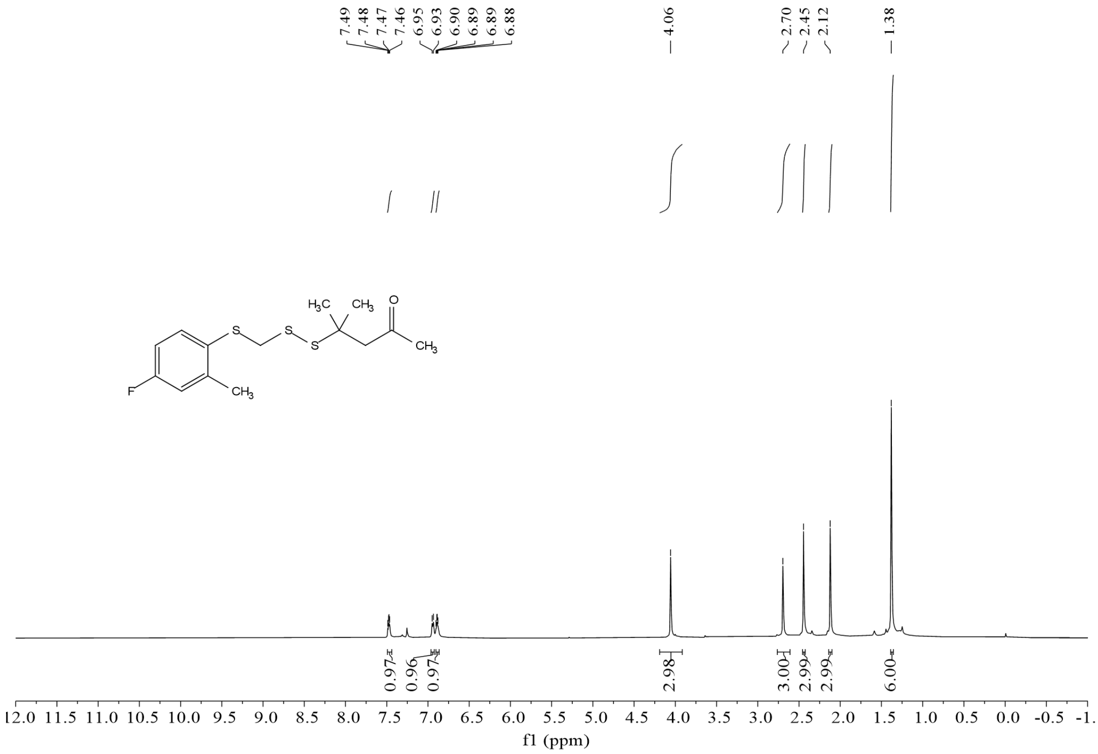

^{1}H NMR of compound 3n

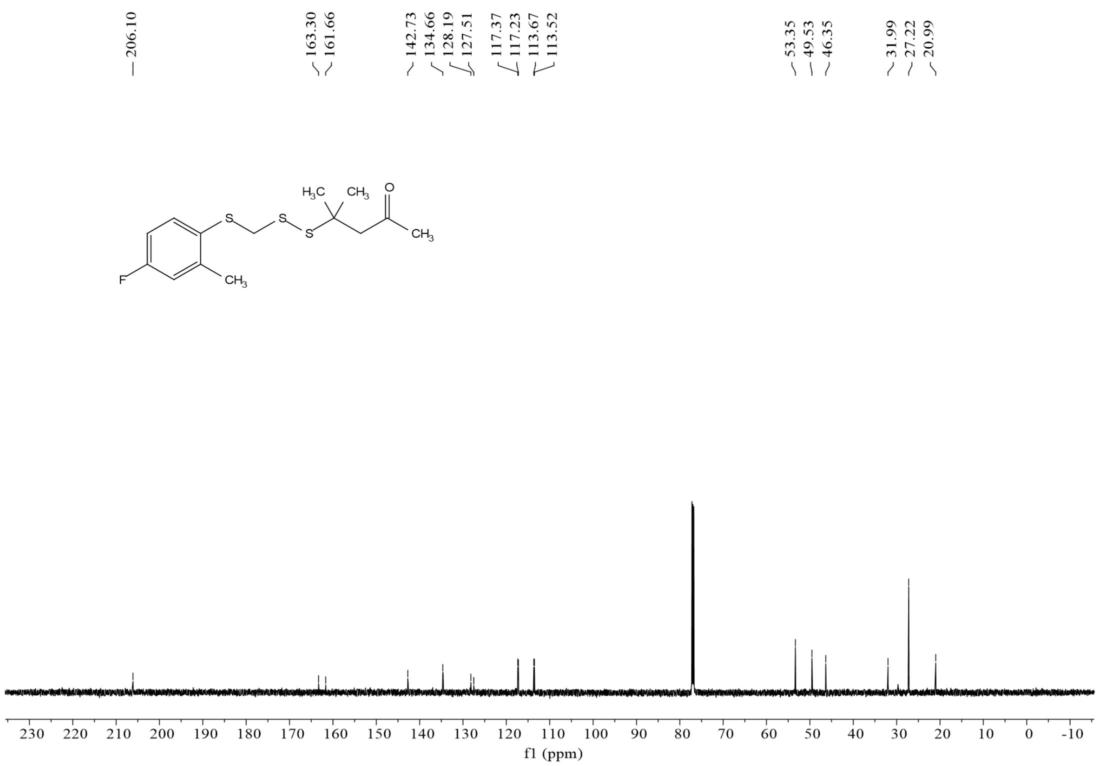

^{13}C NMR of compound 3n

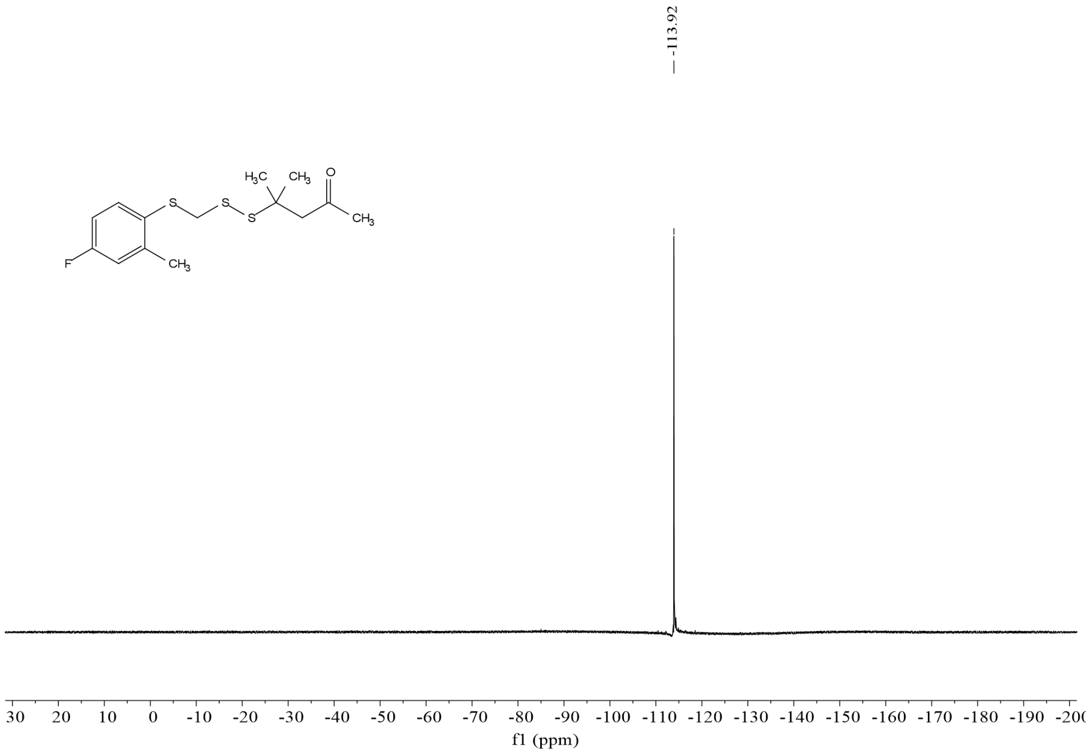

^{19}F NMR of compound 3n

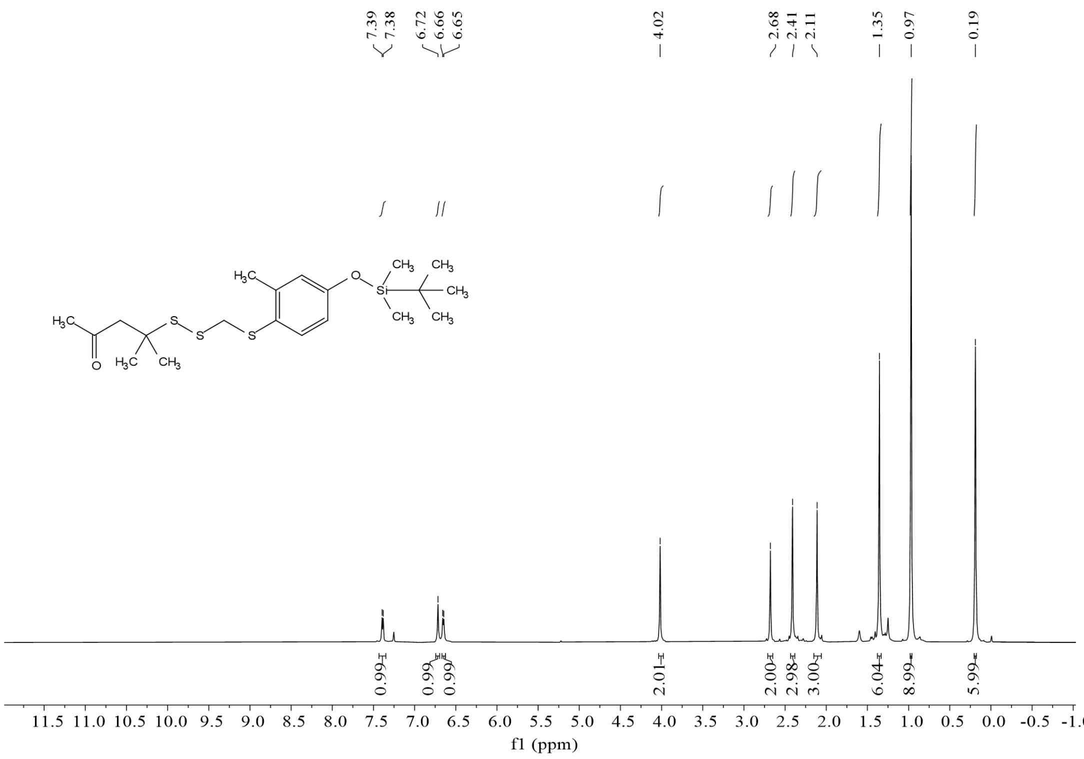

^{1}H NMR of compound 3o

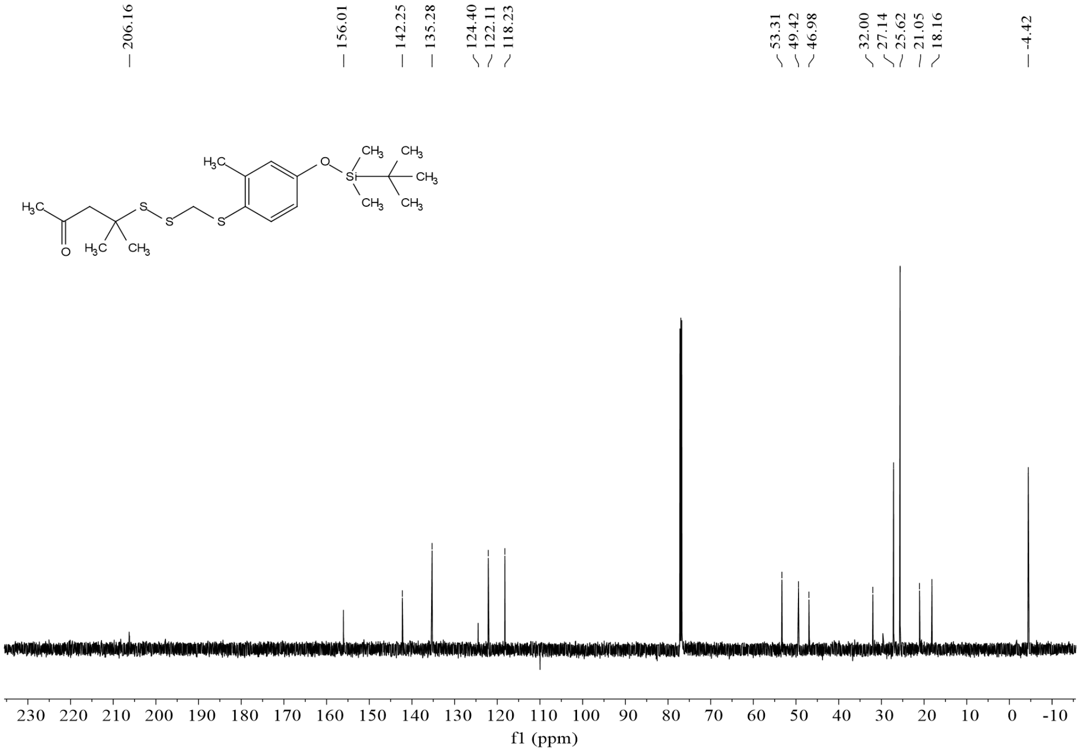

^{13}C NMR of compound 3o

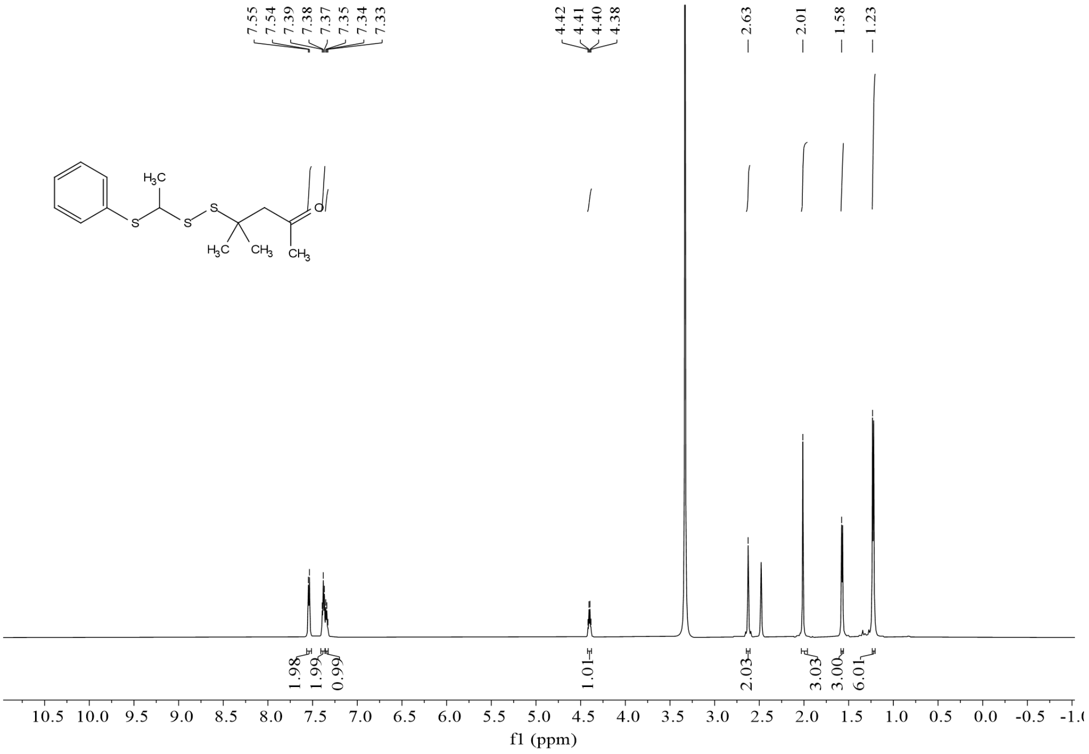

^{1}H NMR of compound 3p

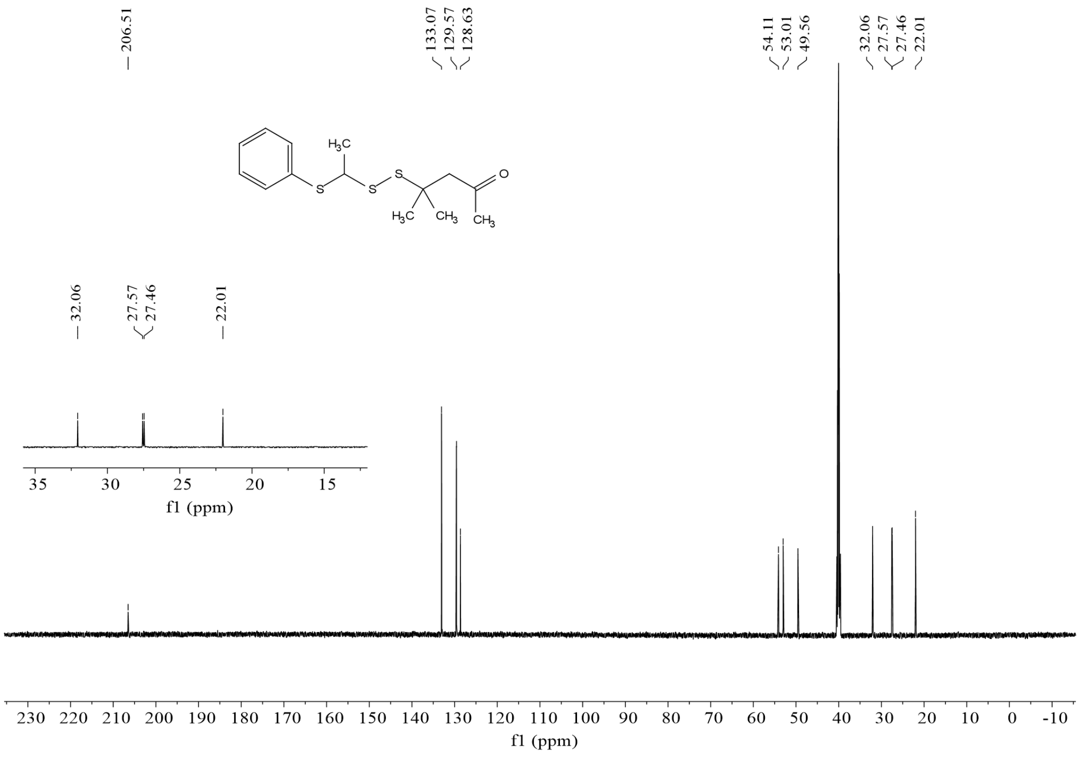

^{13}C NMR of compound 3p

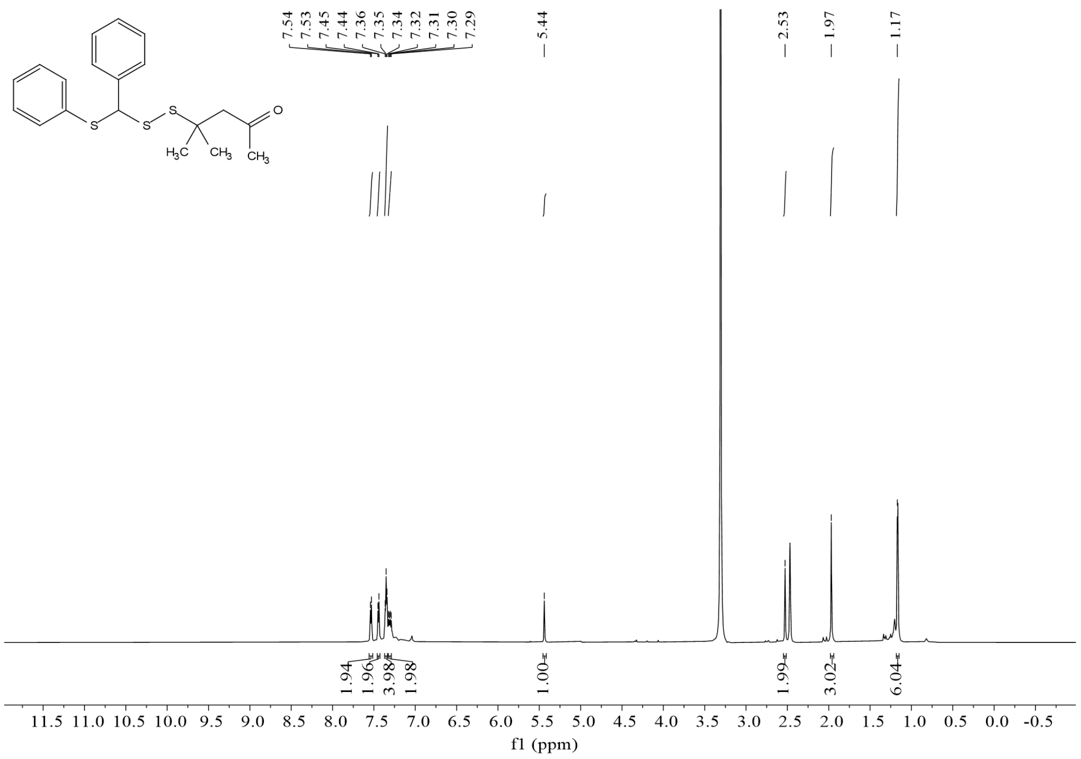

^{1}H NMR of compound 3q

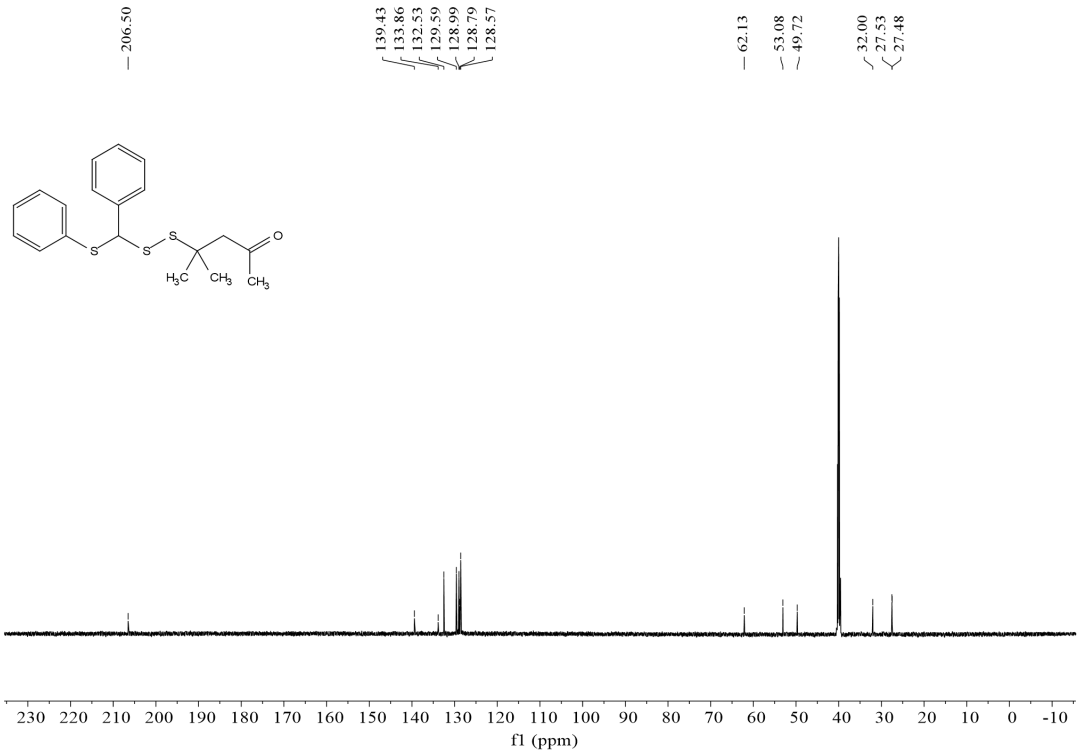

^{13}C NMR of compound 3q

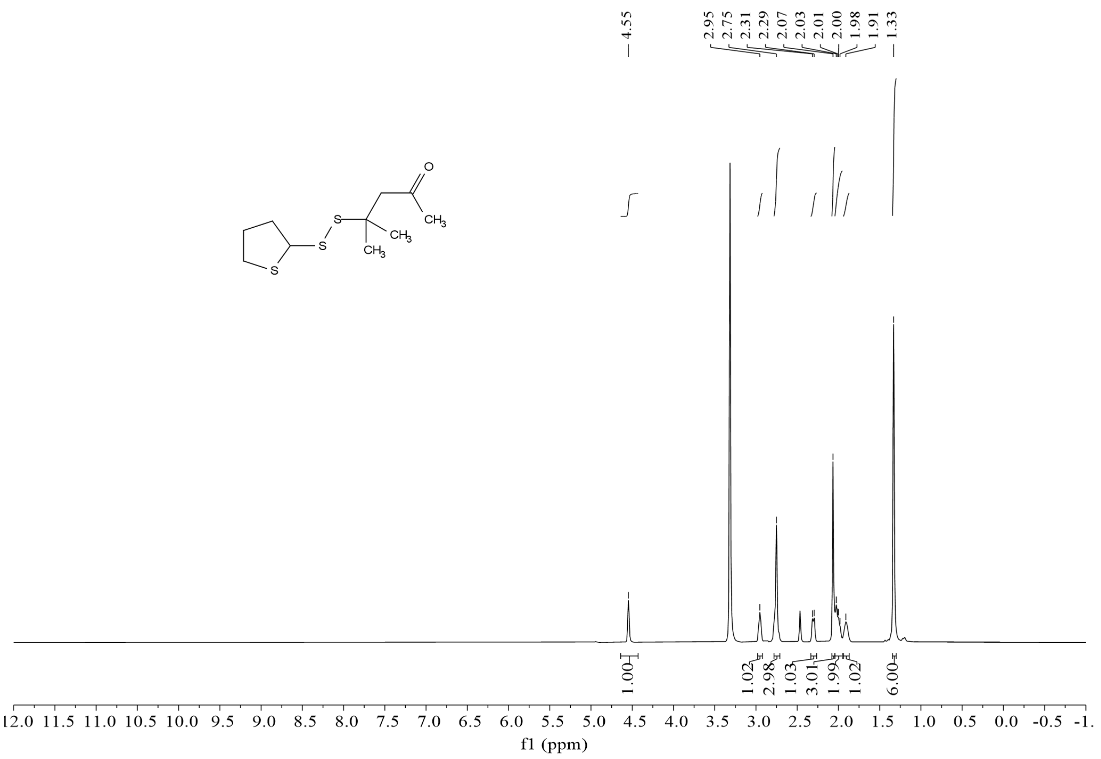

^{1}H NMR of compound 3r

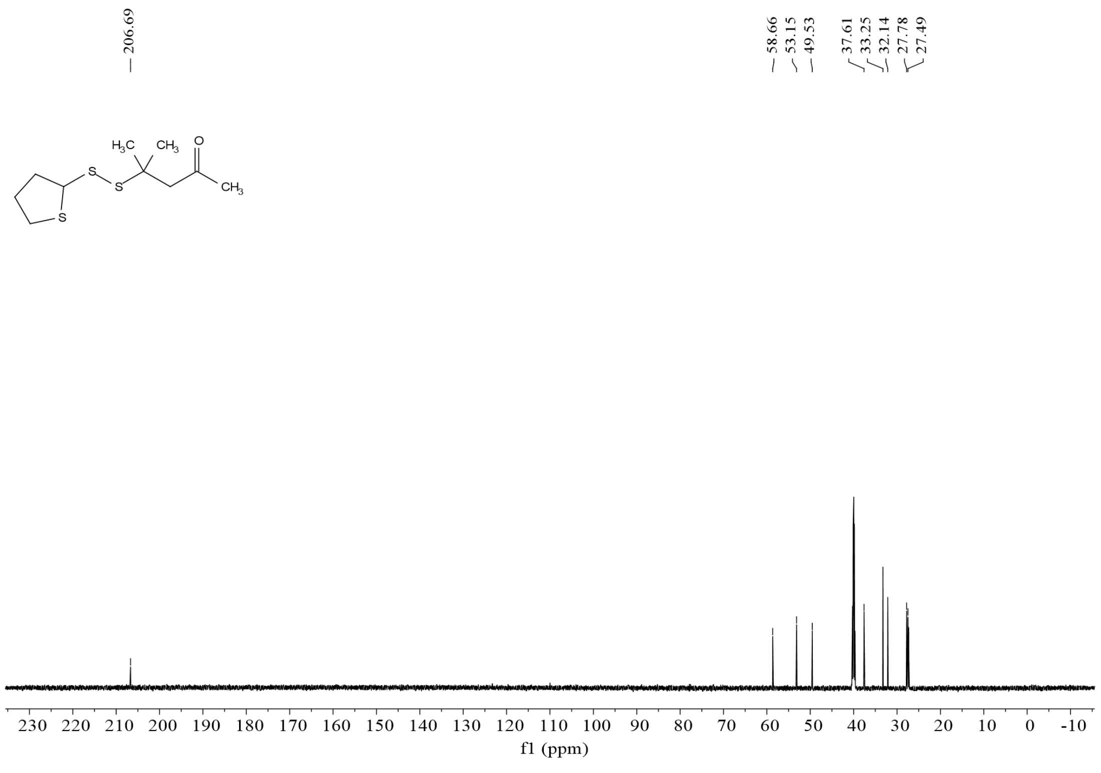

^{13}C NMR of compound 3r

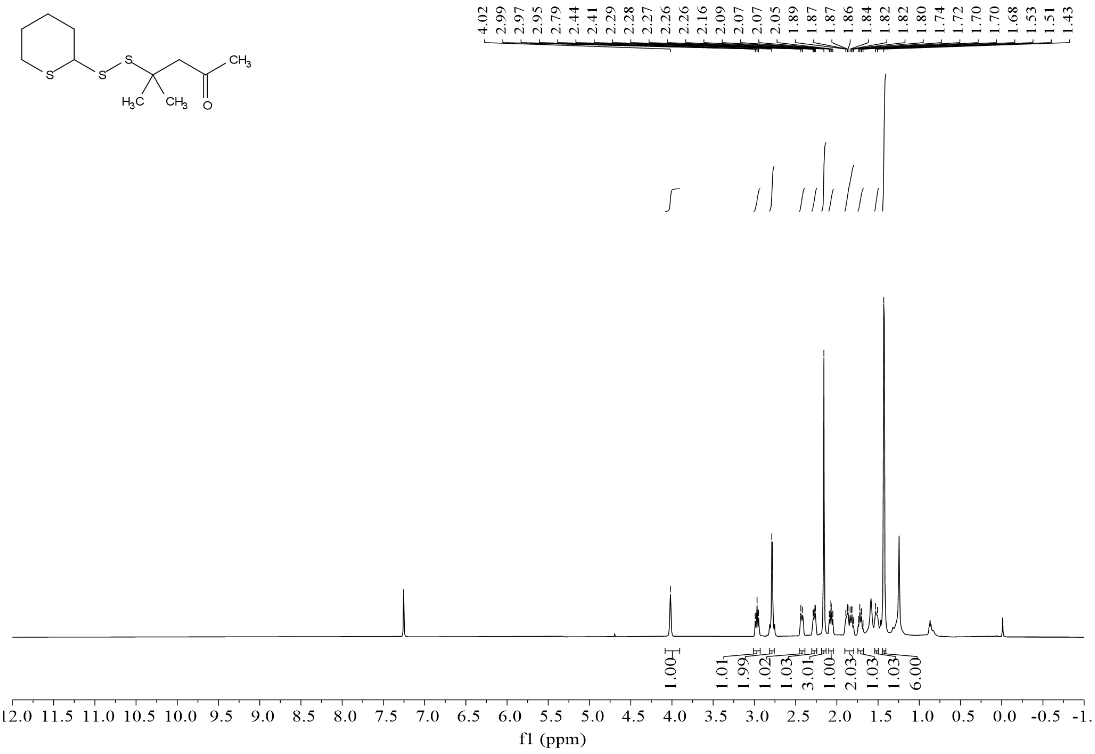

^{1}H NMR of compound 3s

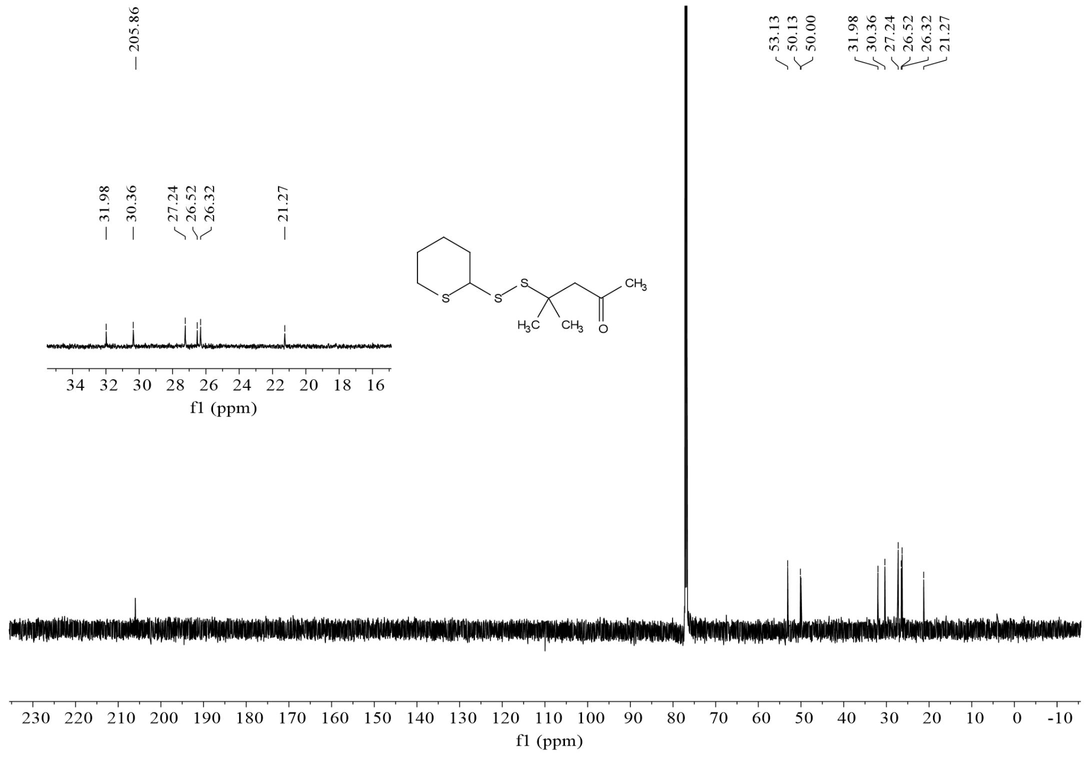

^{13}C NMR of compound 3s

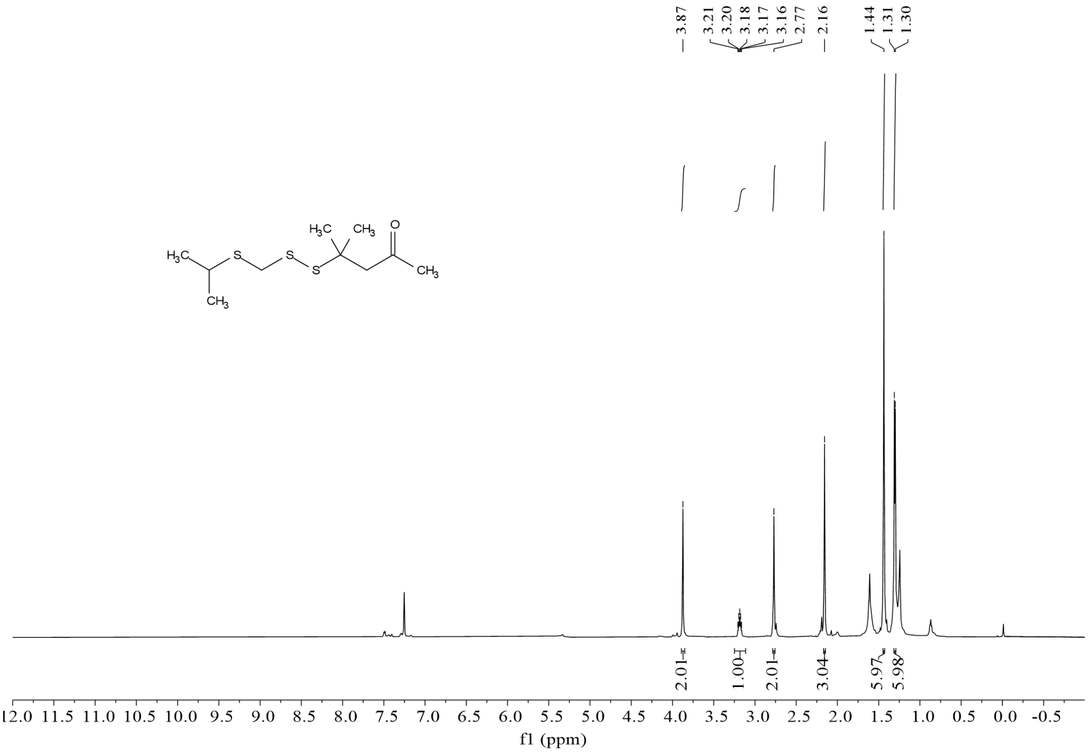

^{1}H NMR of compound 3t

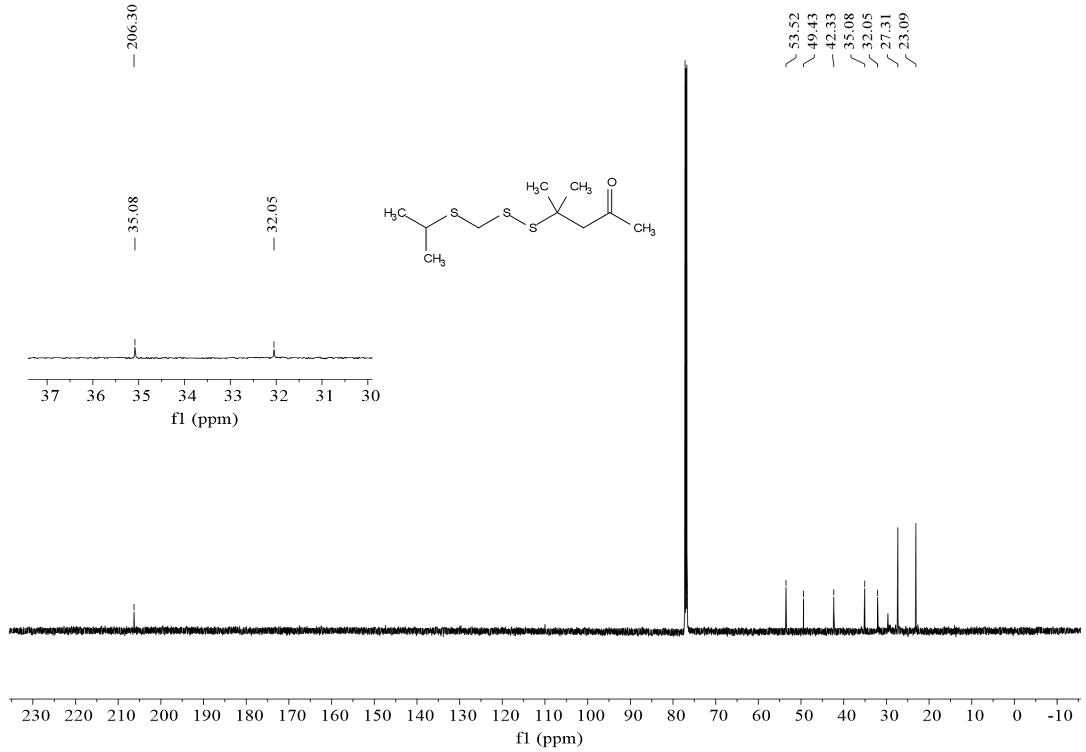

^{13}C NMR of compound 3t

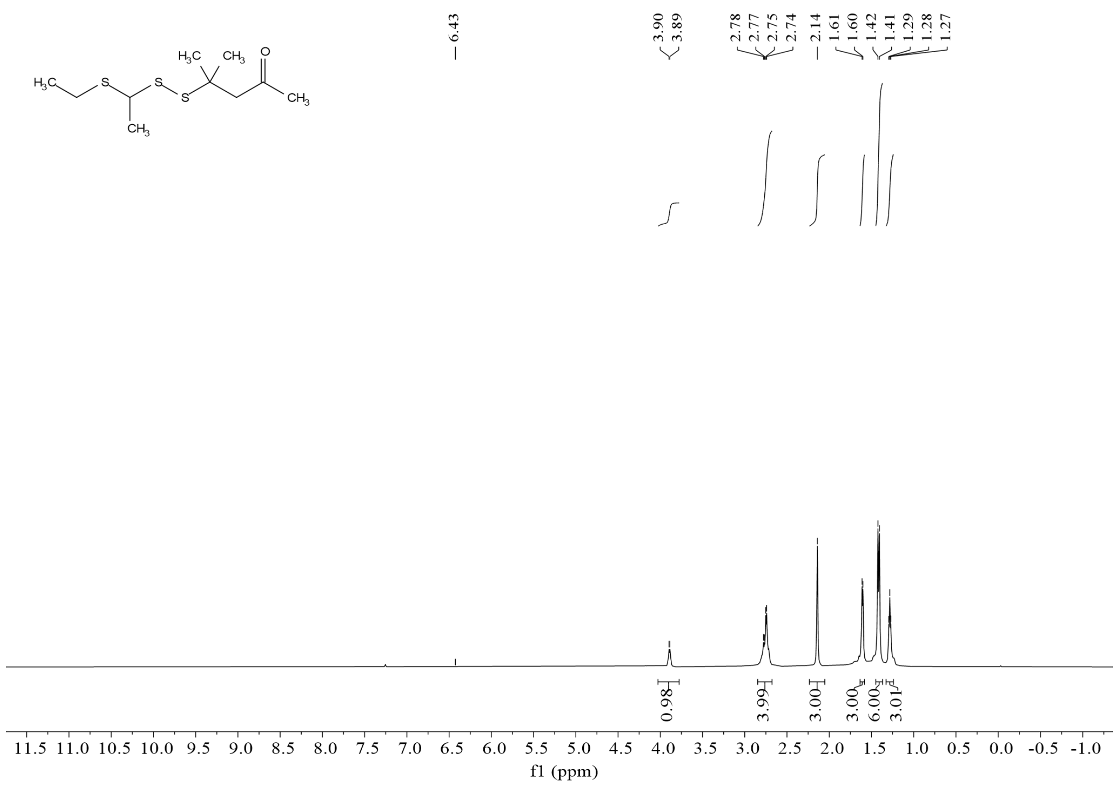

^{1}H NMR of compound 3u

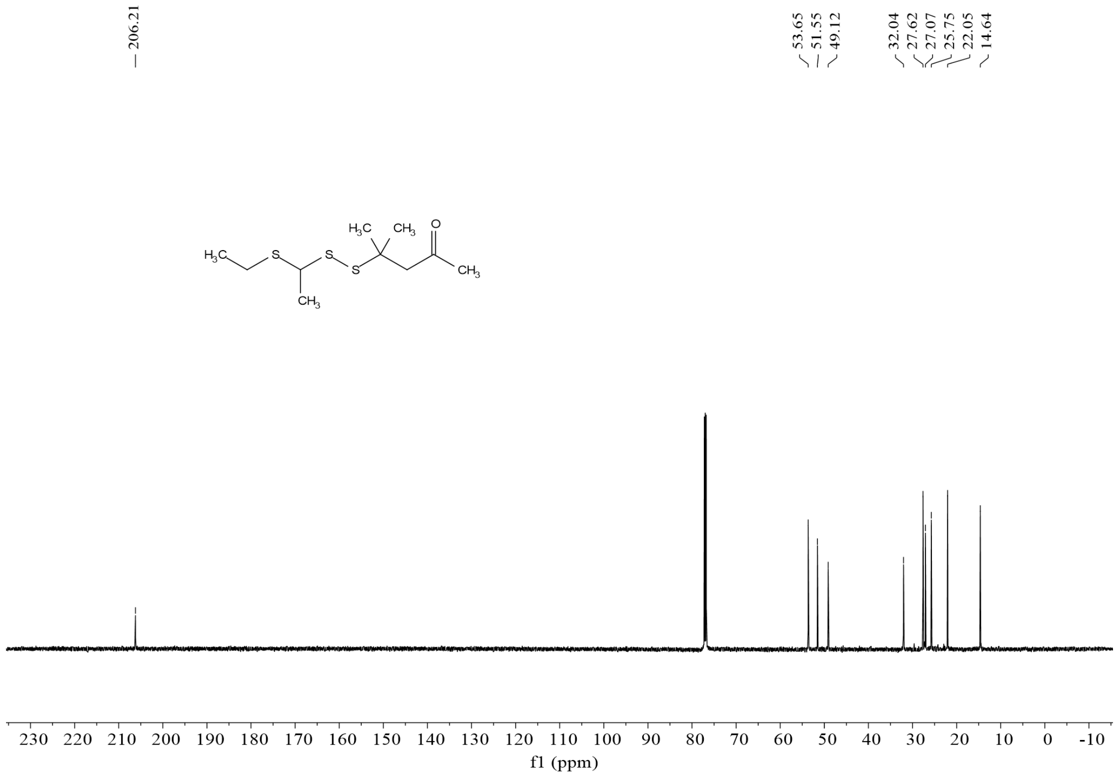

^{13}C NMR of compound 3u

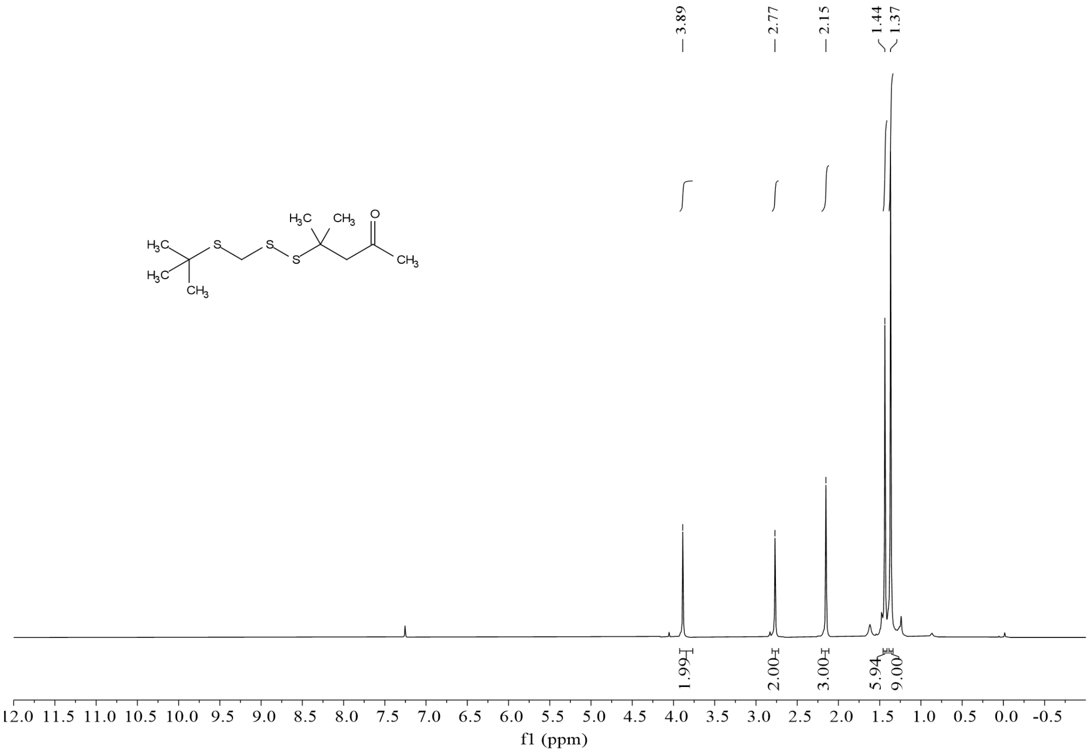

^{1}H NMR of compound 3v

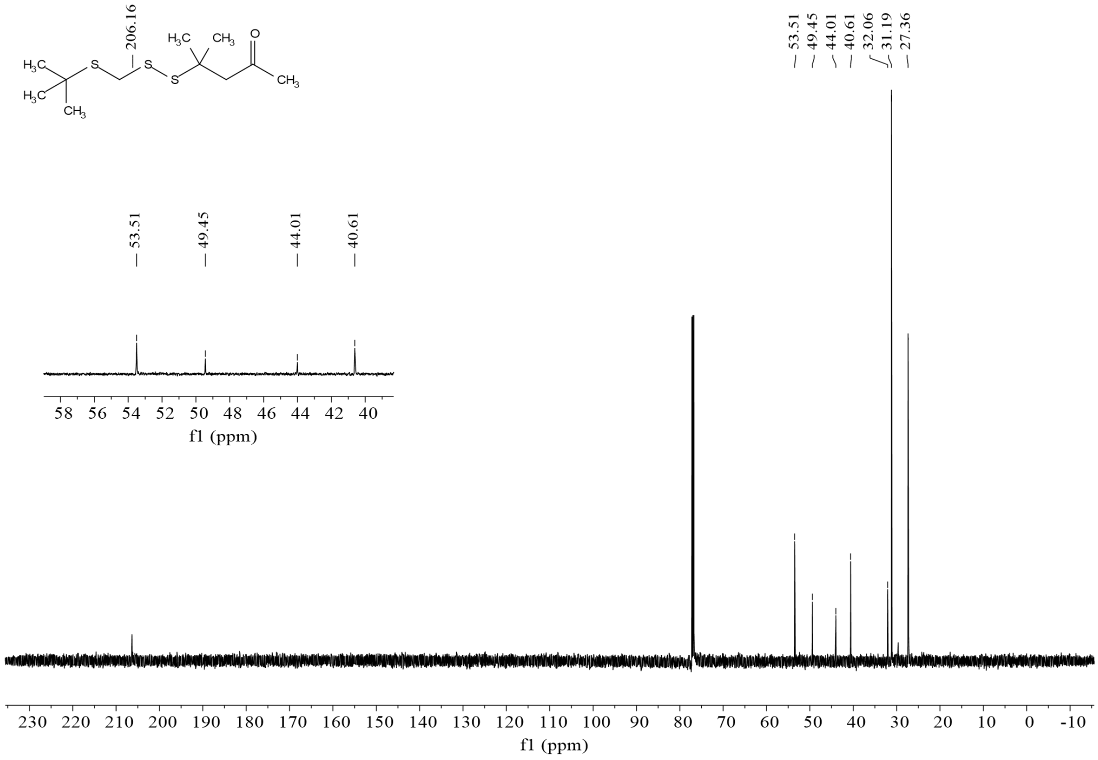

^{13}C NMR of compound 3v

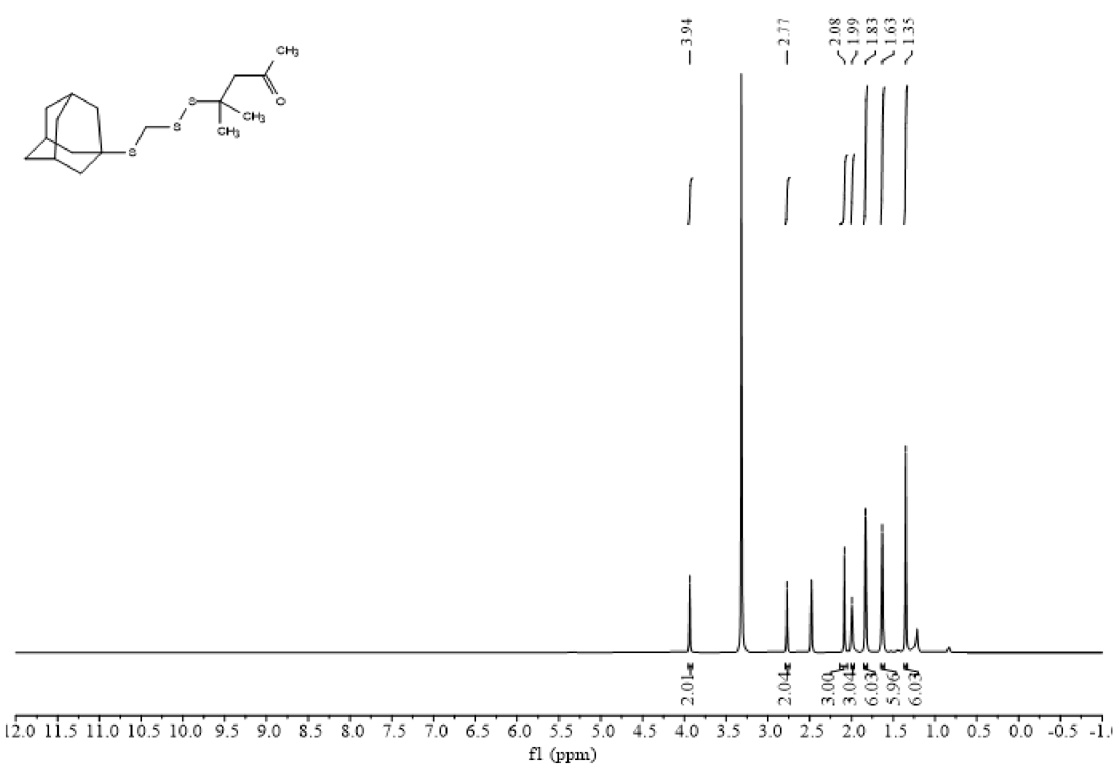

^{1}H NMR of compound 3w

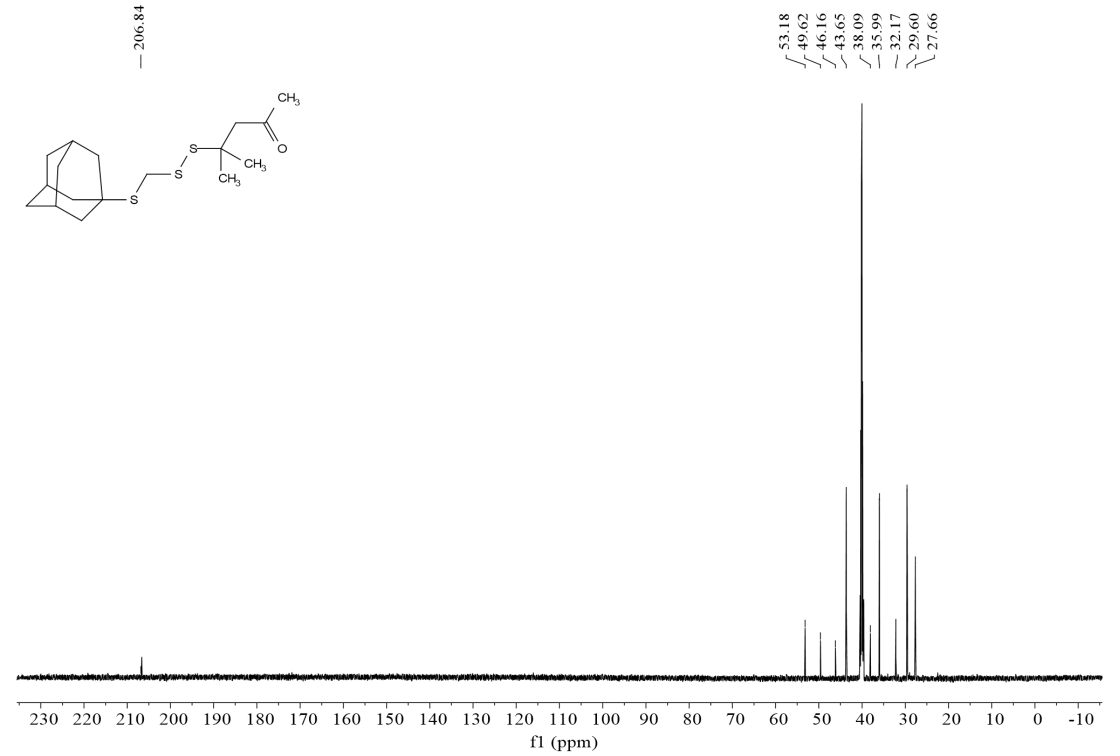

^{13}C NMR of compound 3w

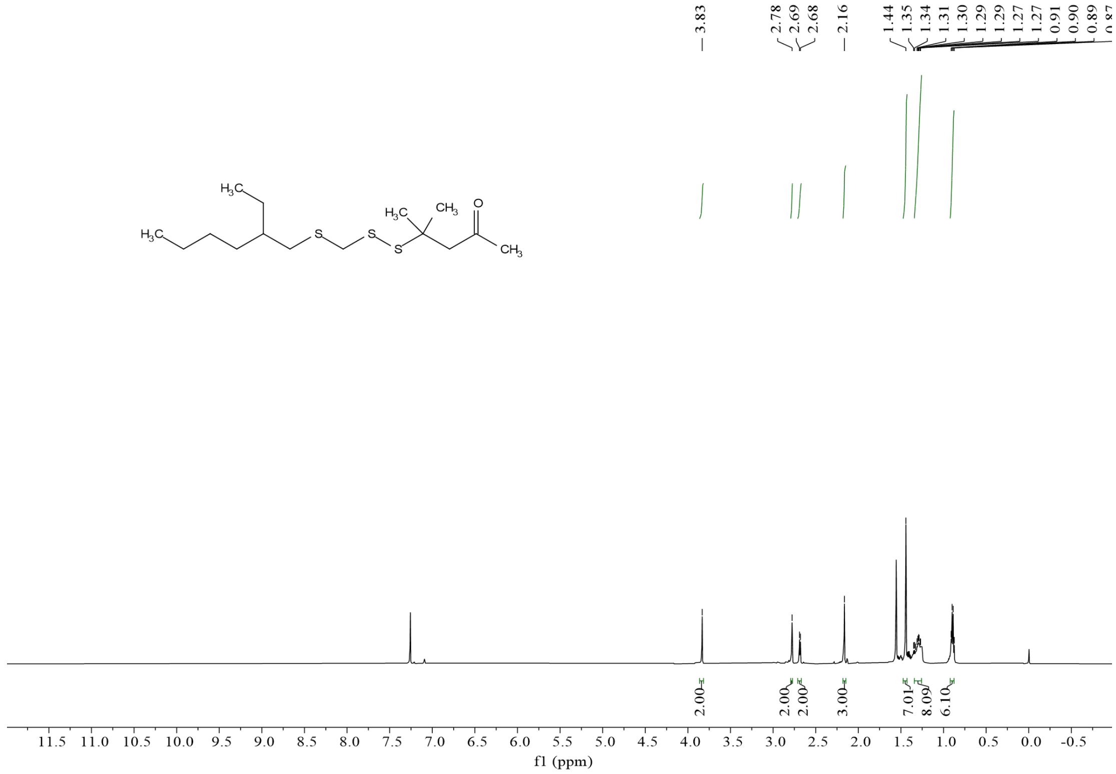

^{1}H NMR of compound 3x

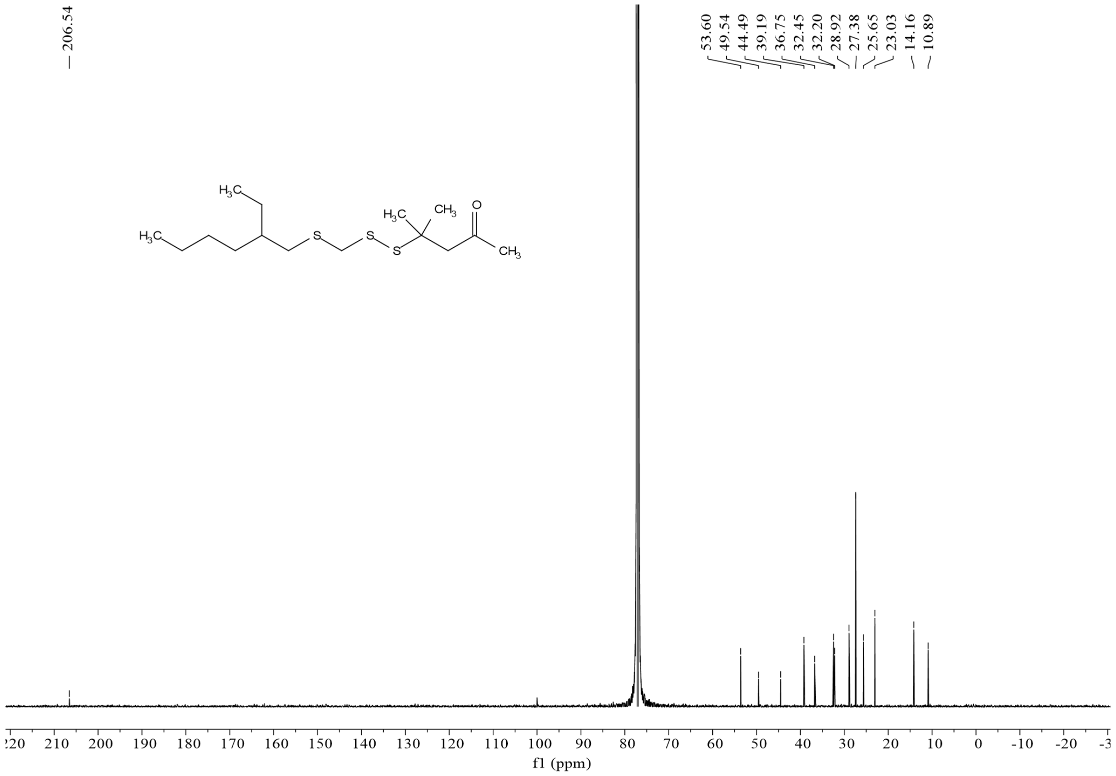

^{13}C NMR of compound 3x

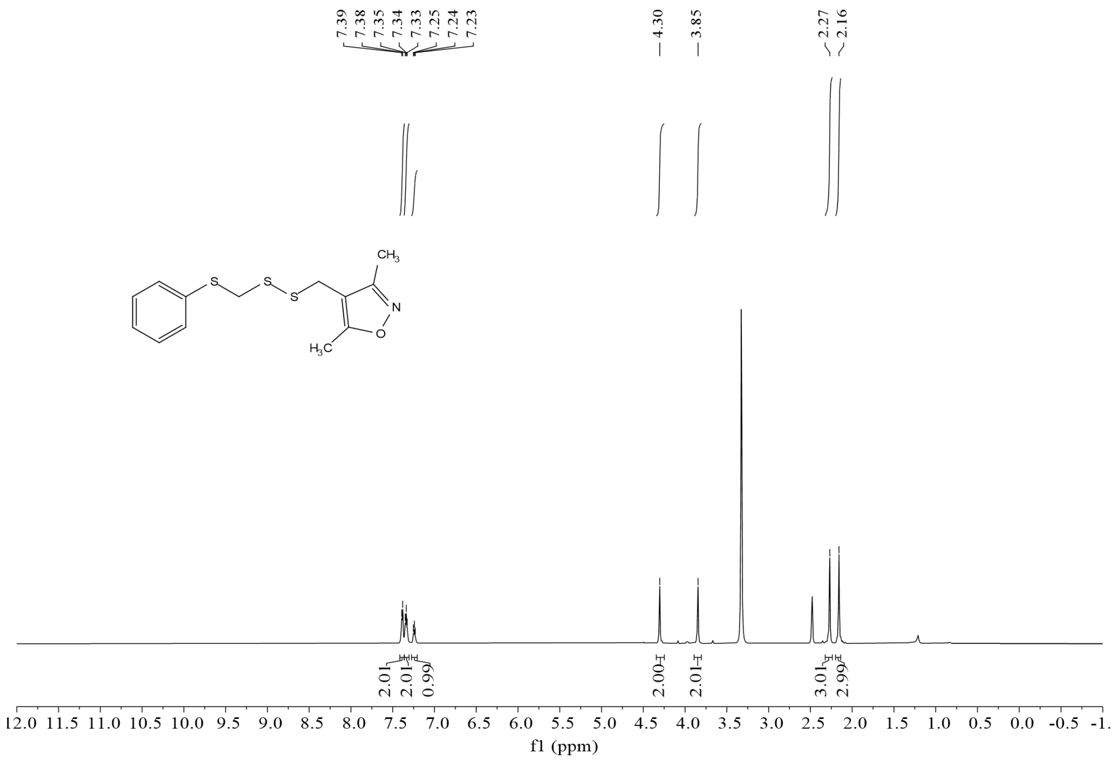

^{1}H NMR of compound 5a

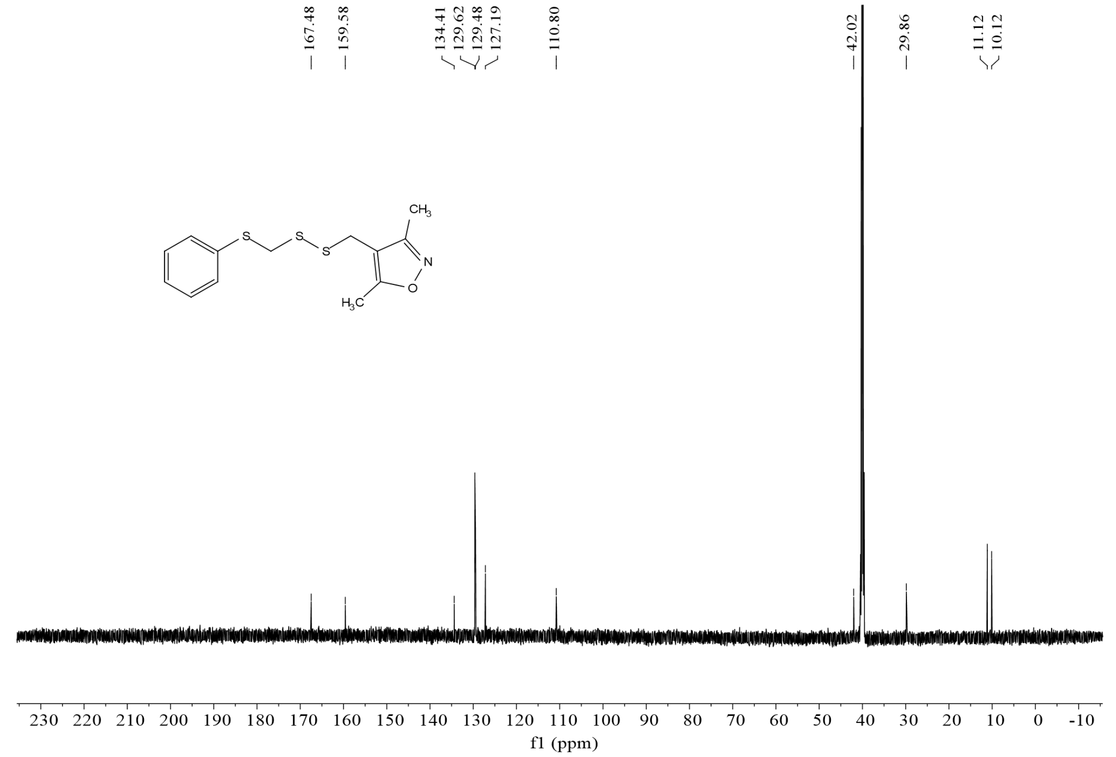

^{13}C NMR of compound 5a

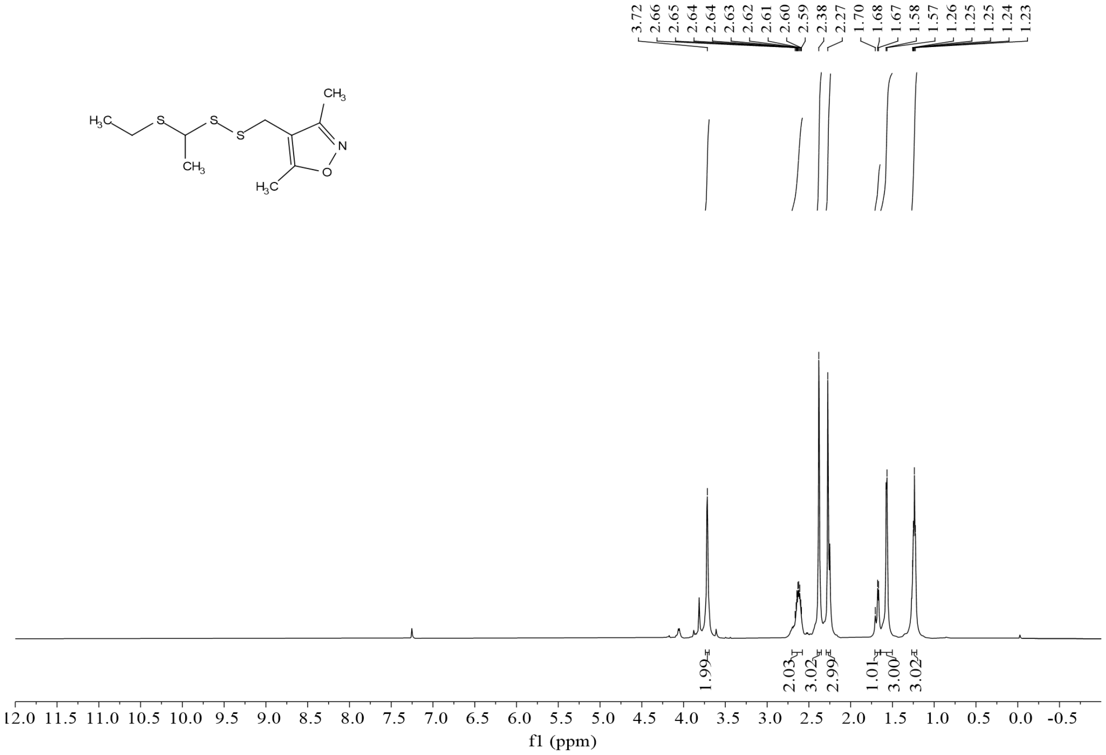

^{1}H NMR of compound 5b

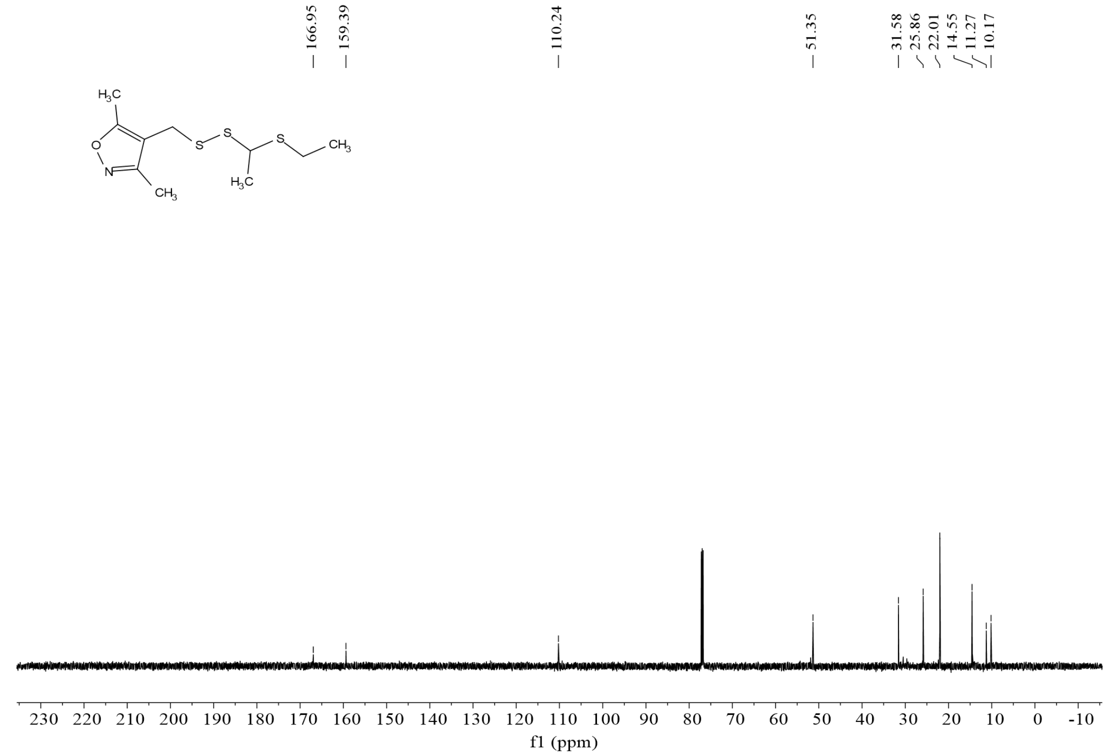

^{13}C NMR of compound 5b

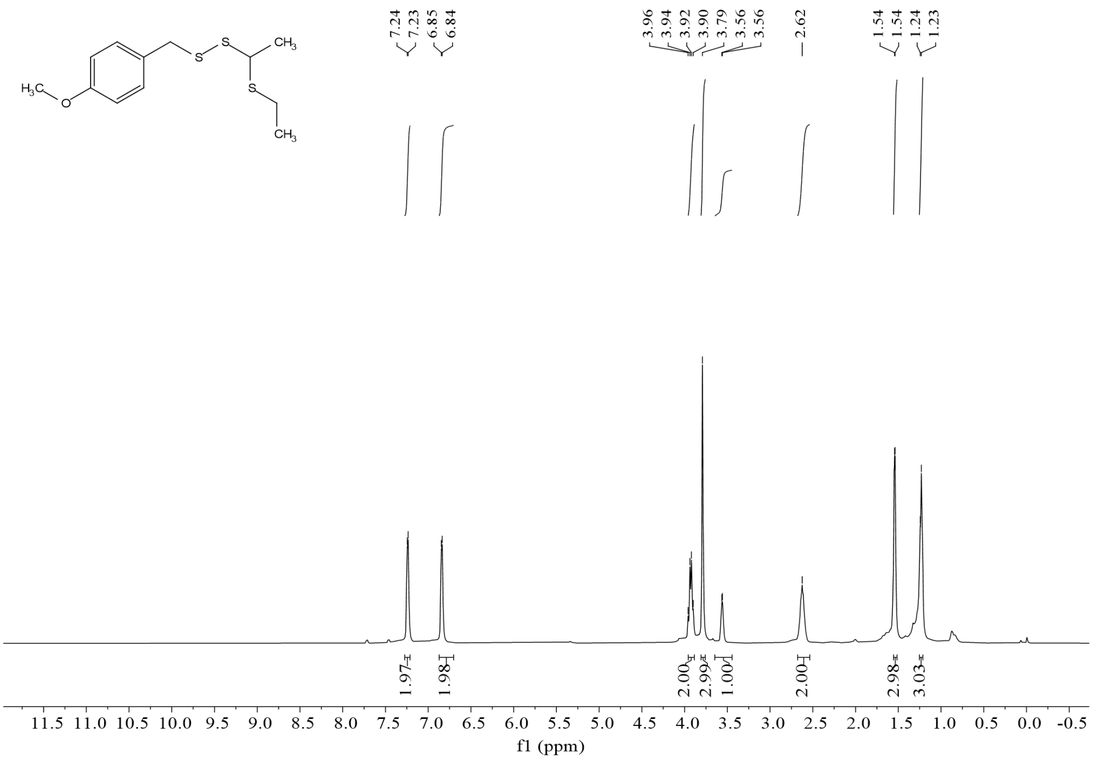

^{1}H NMR of compound 5c

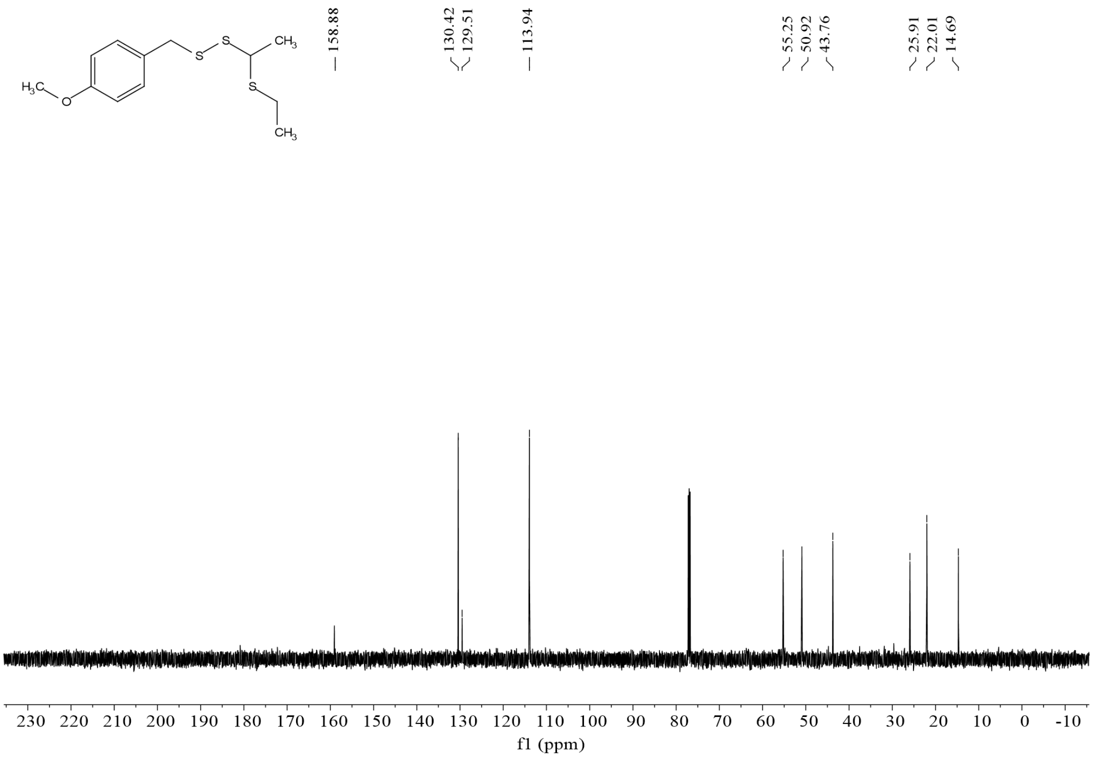

^{13}C NMR of compound 5c

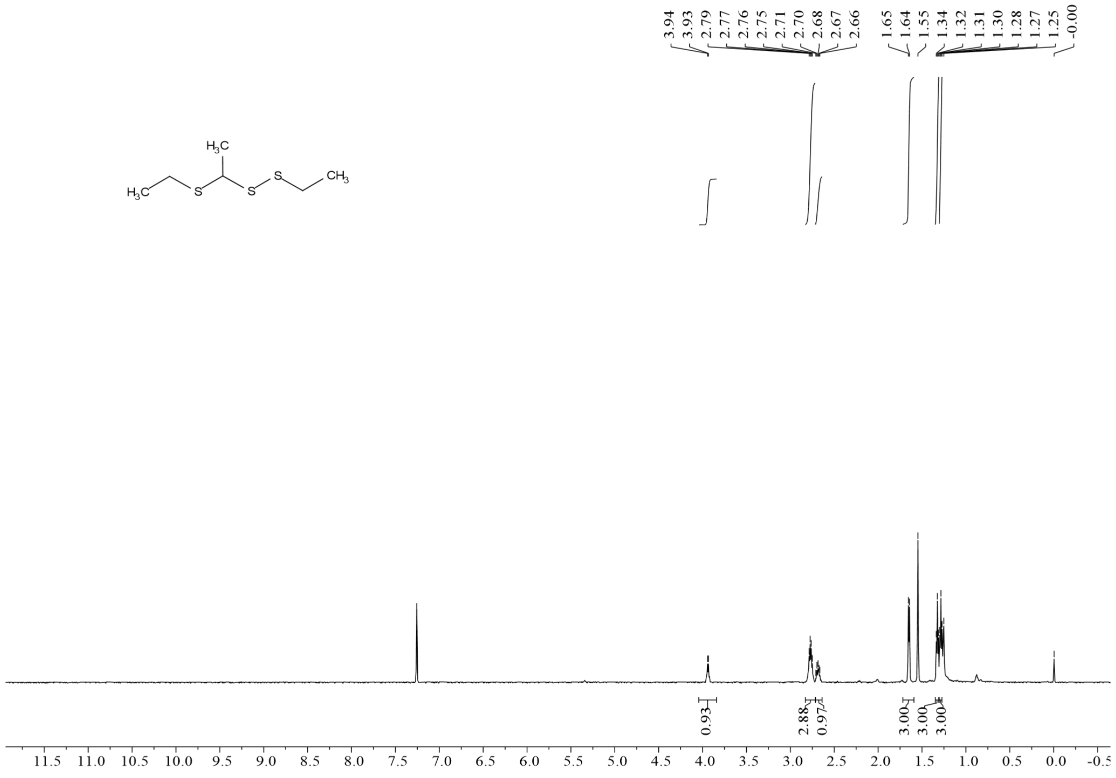

^{1}H NMR of compound 5d

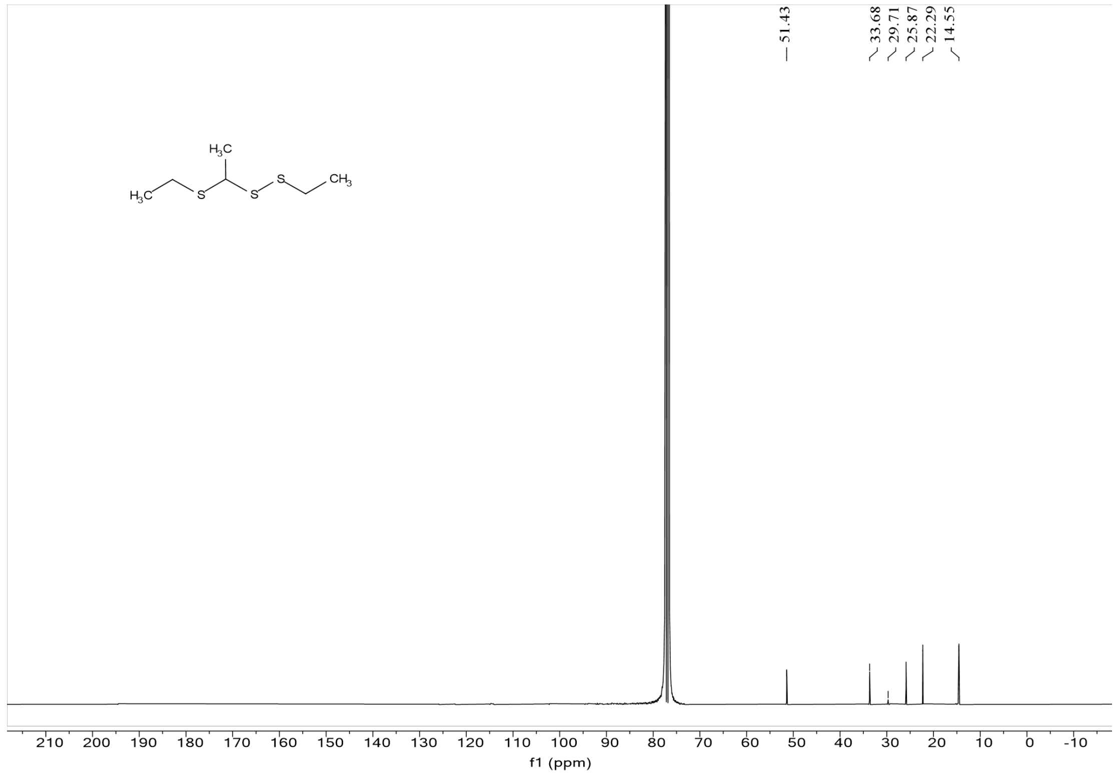

^{13}C NMR of compound 5d

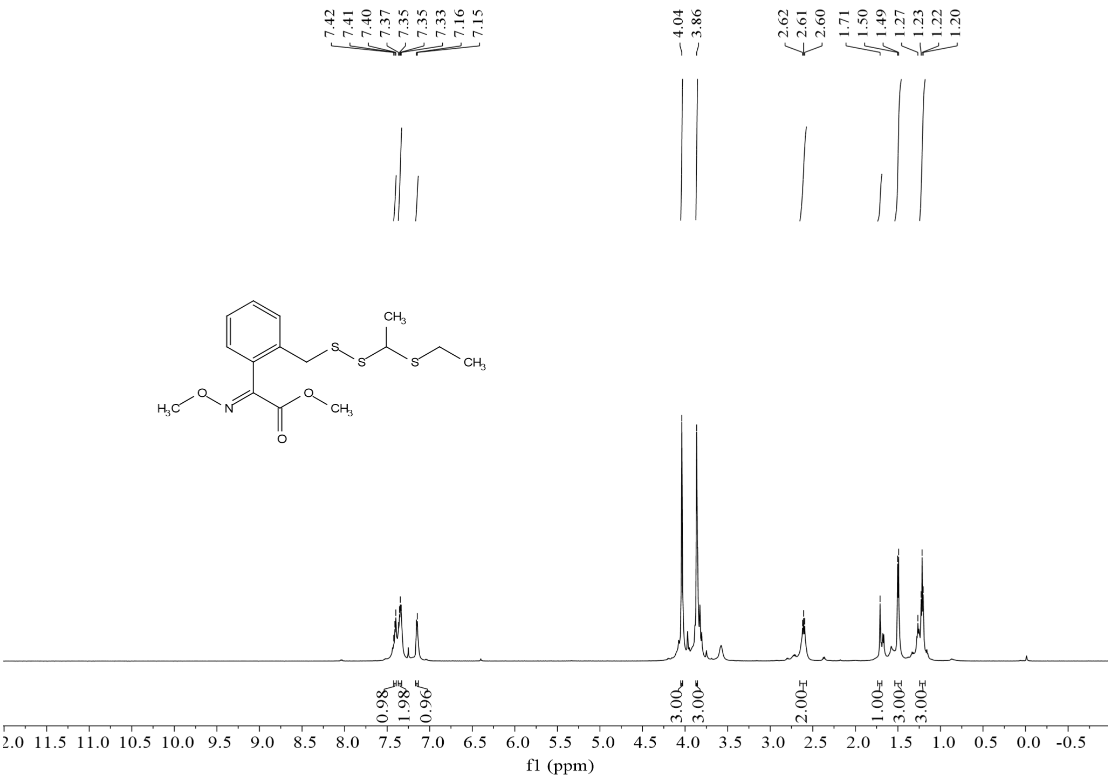

^{1}H NMR of compound 5e

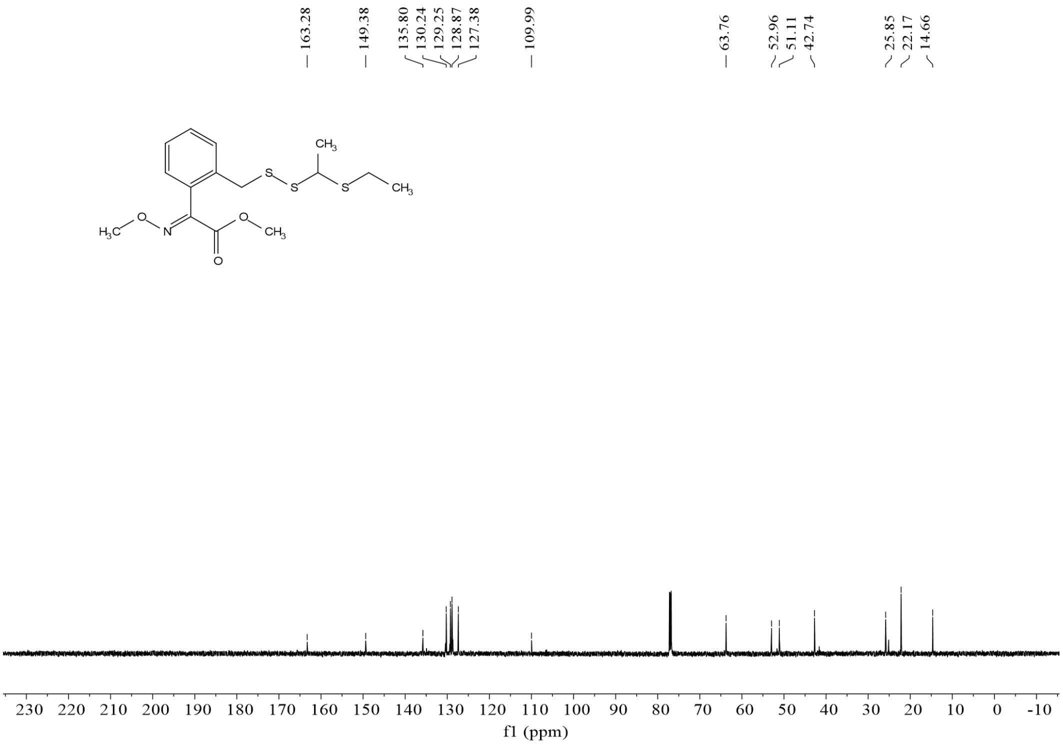

^{13}C NMR of compound 5e

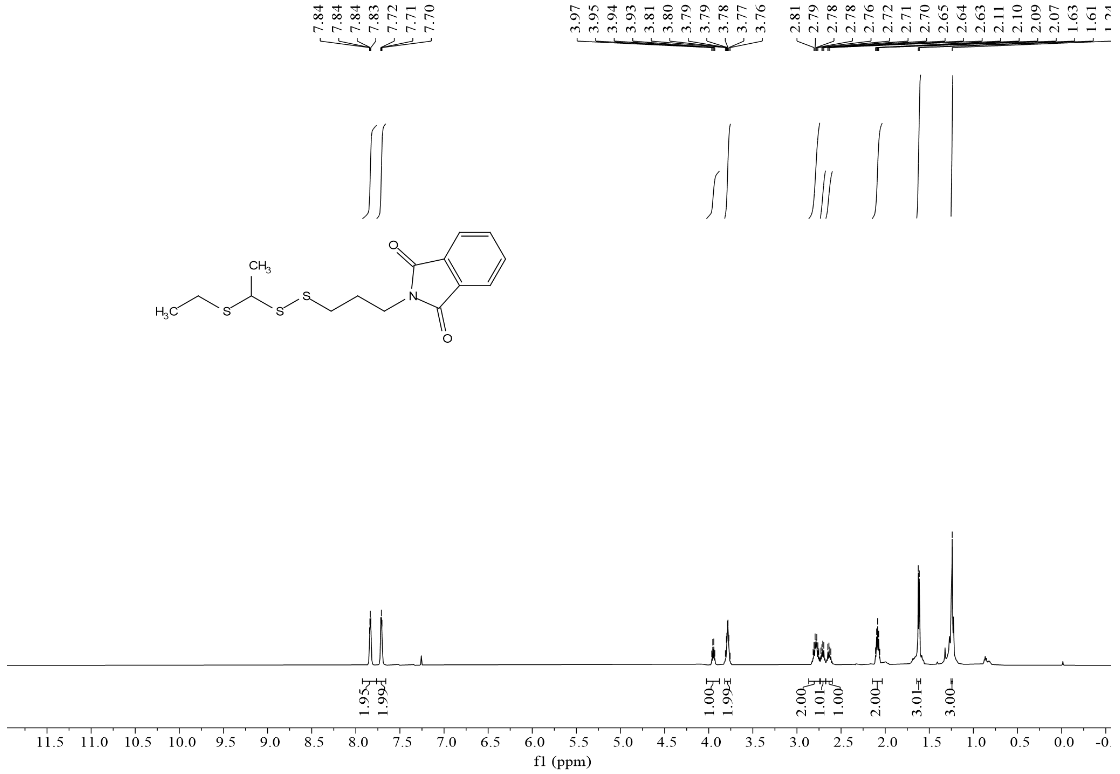

^{1}H NMR of compound 5f

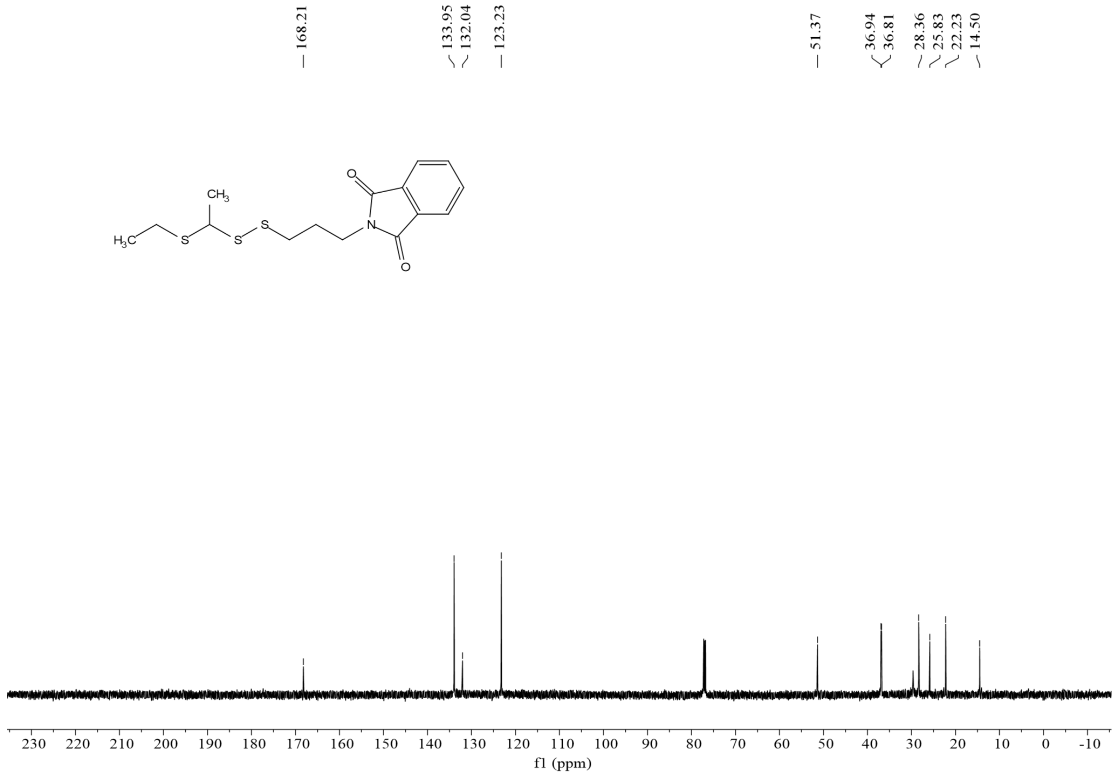

^{13}C NMR of compound 5f

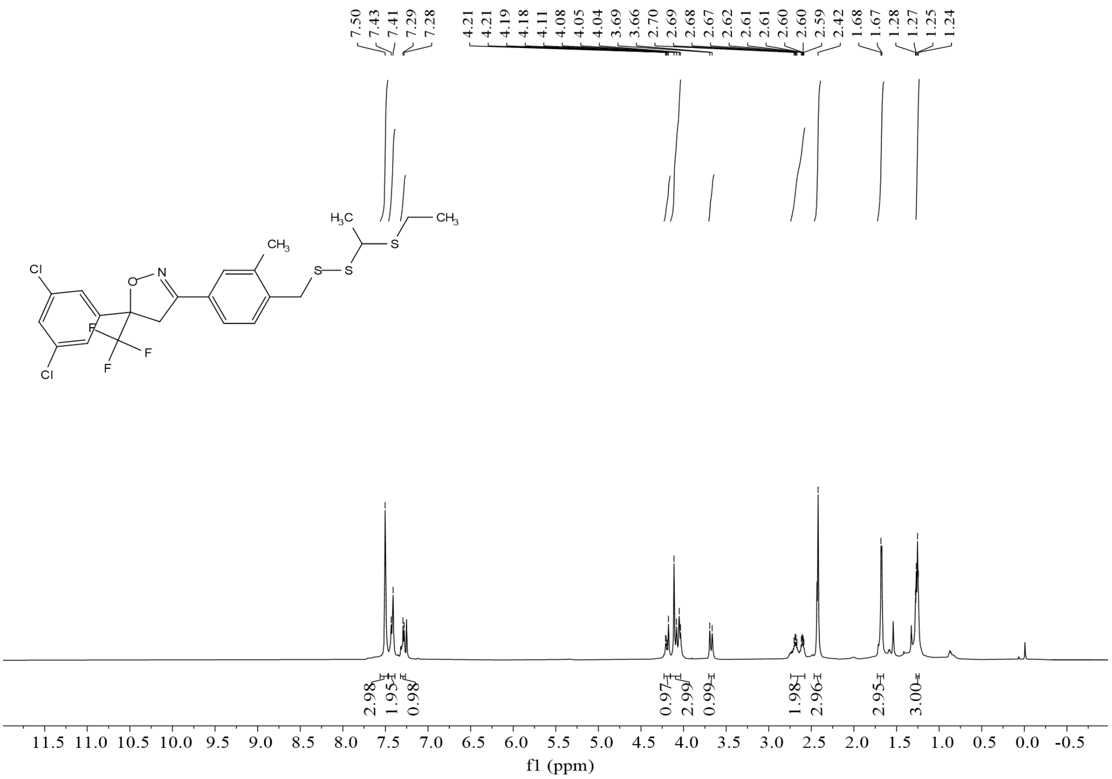

^{1}H NMR of compound 5g

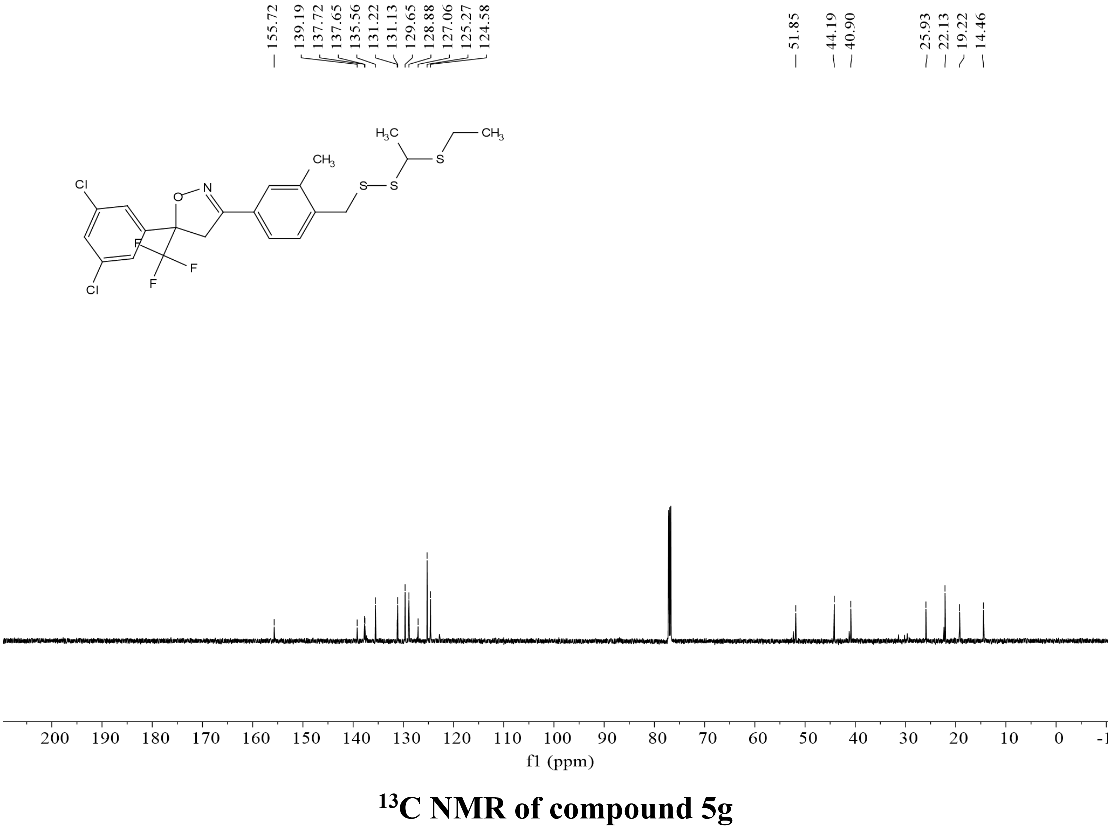

^{13}C NMR of compound 5g

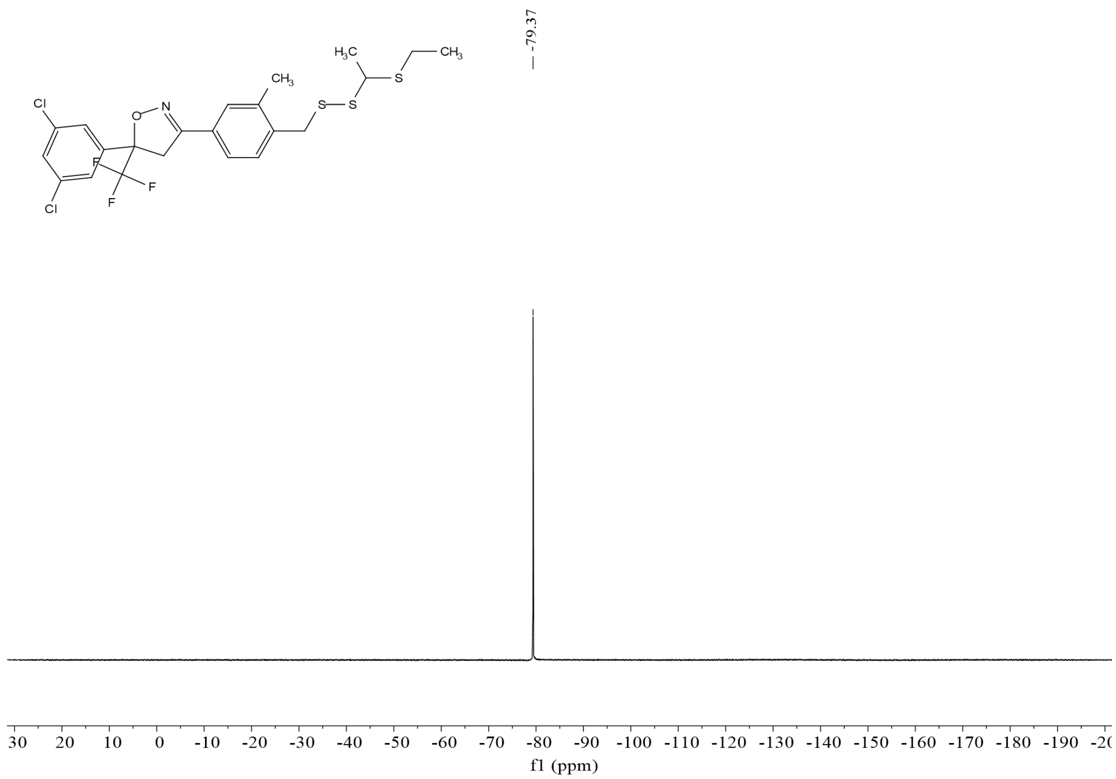

^{19}F NMR of compound 5g

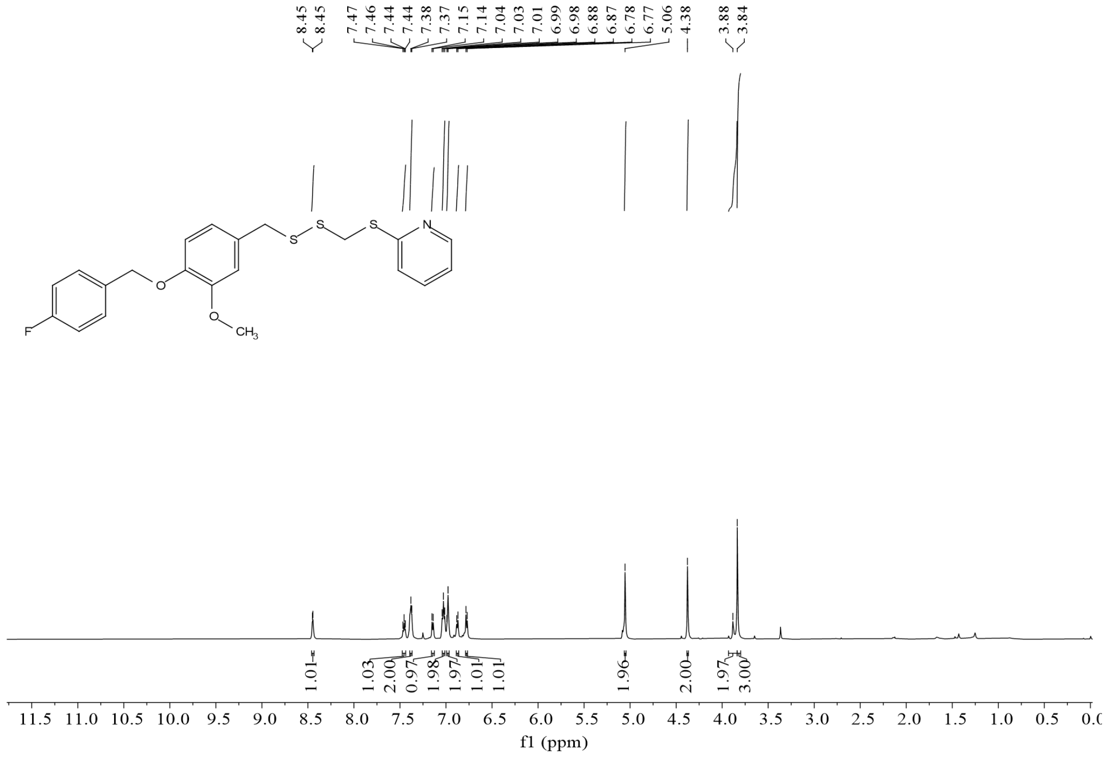

^{1}H NMR of compound 5h

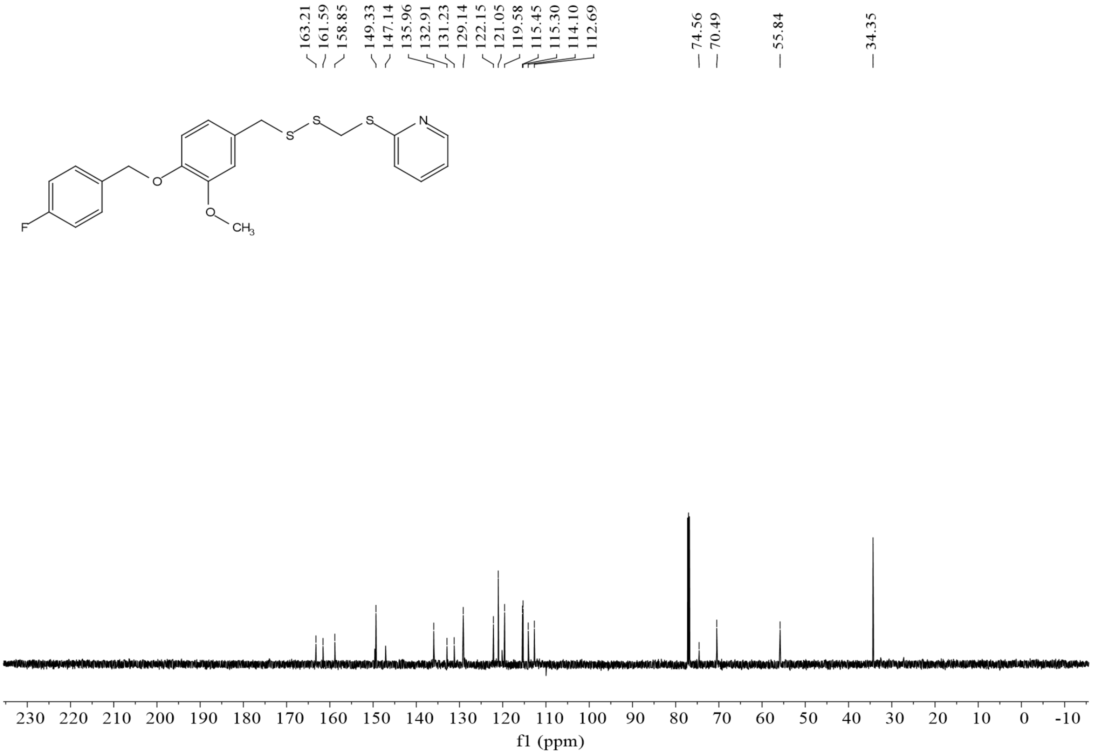

^{13}C NMR of compound 5h

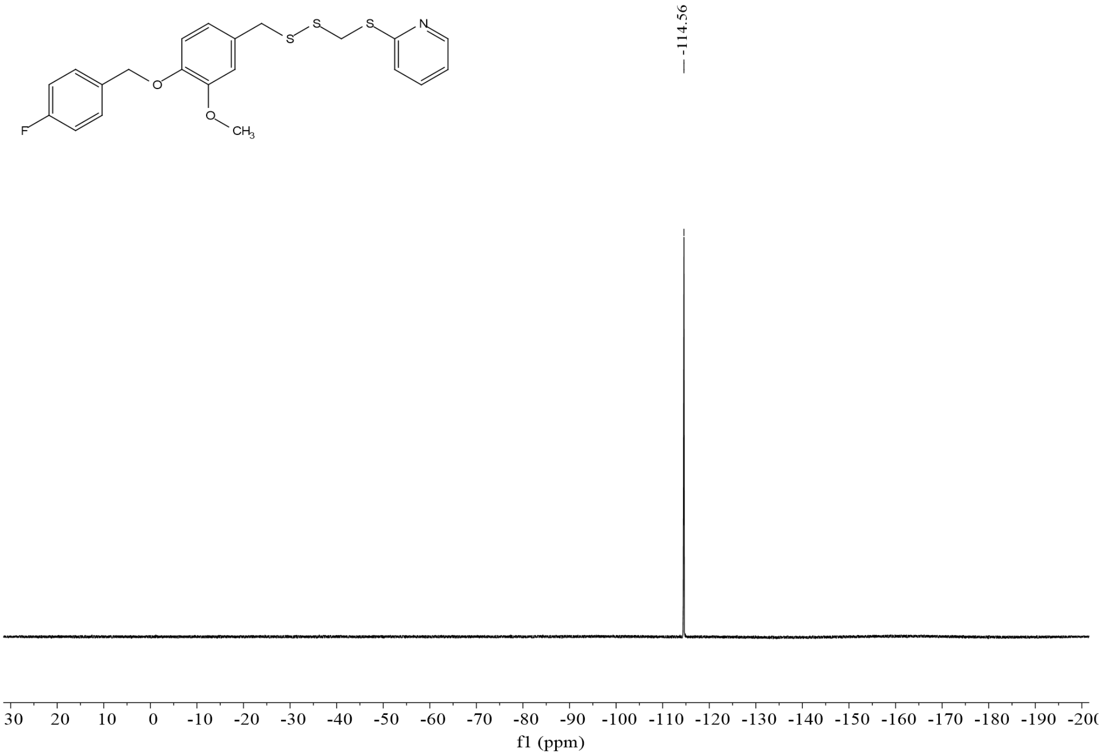

^{19}F NMR of compound 5h

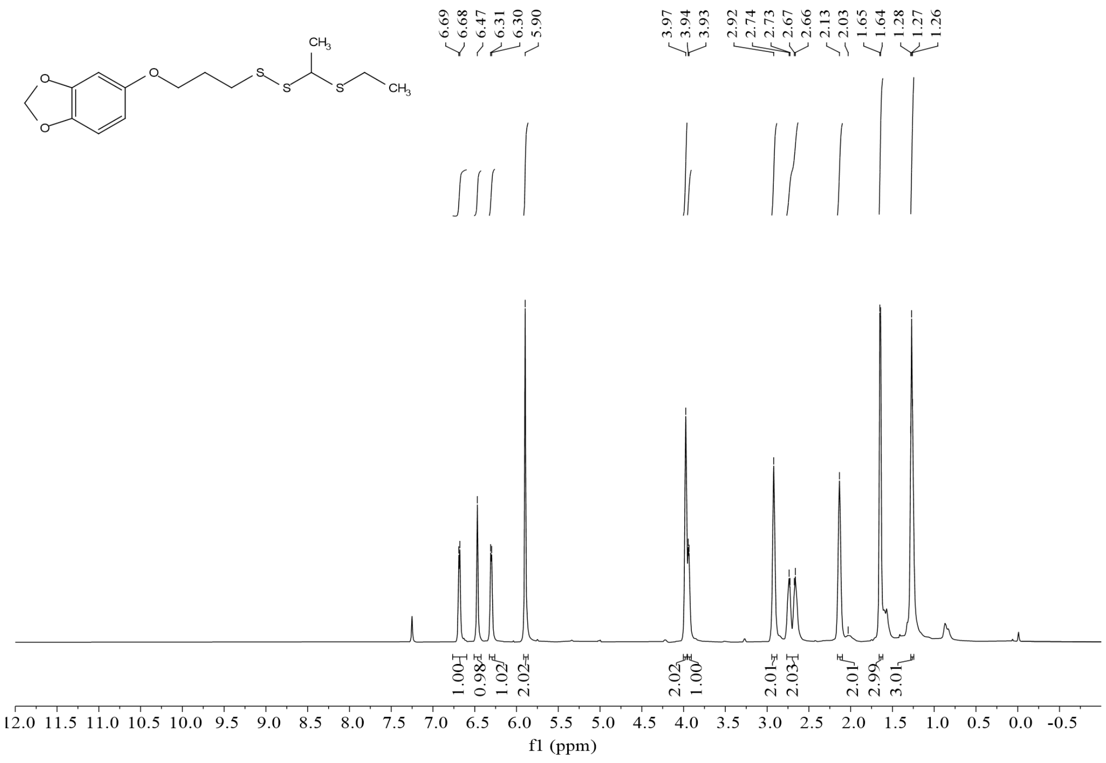

^{1}H NMR of compound 5i

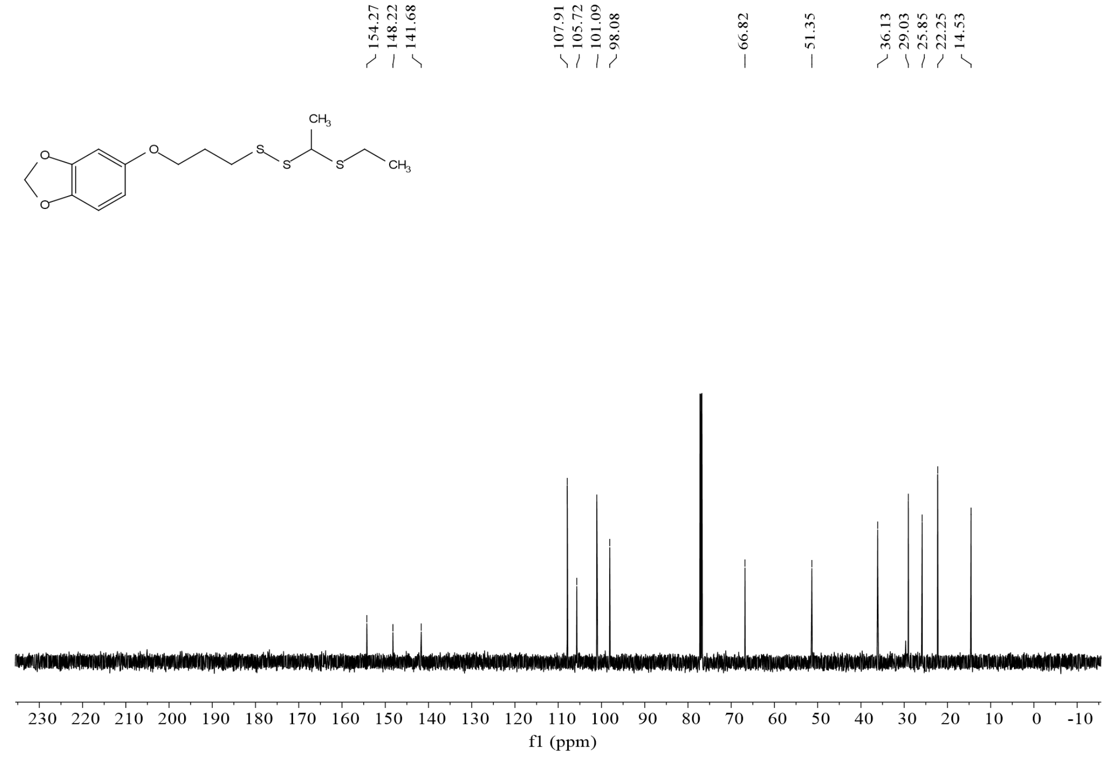

^{13}C NMR of compound 5i

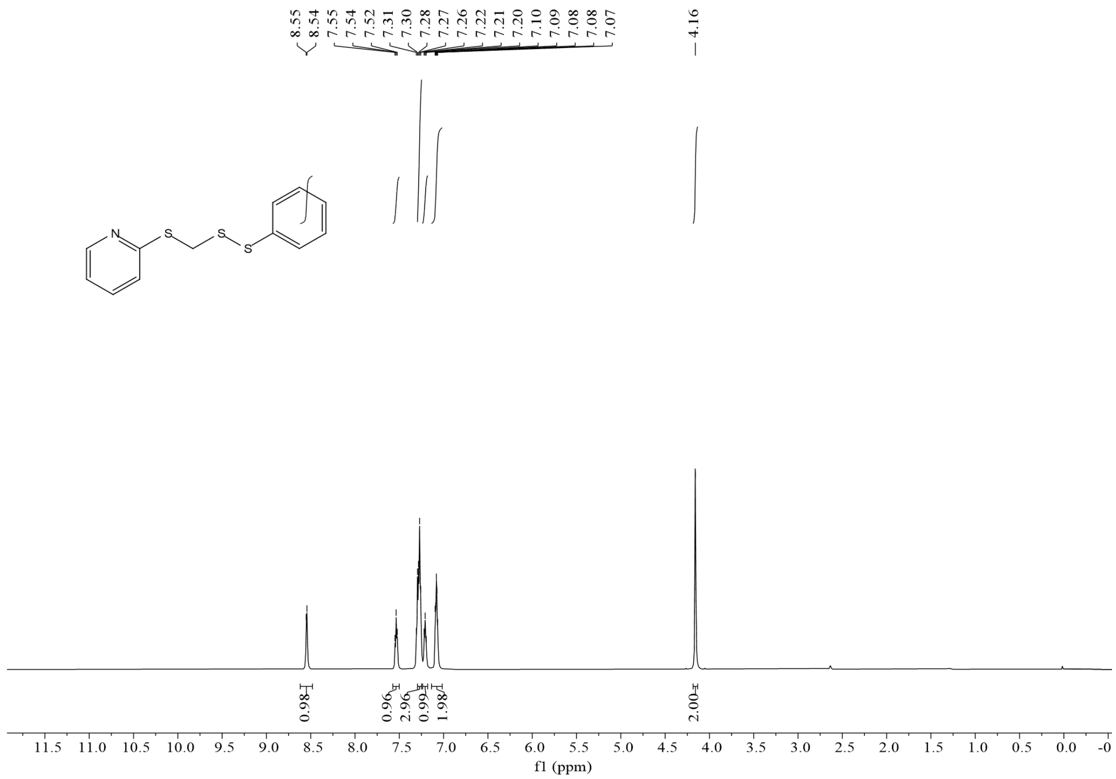

^{1}H NMR of compound 9

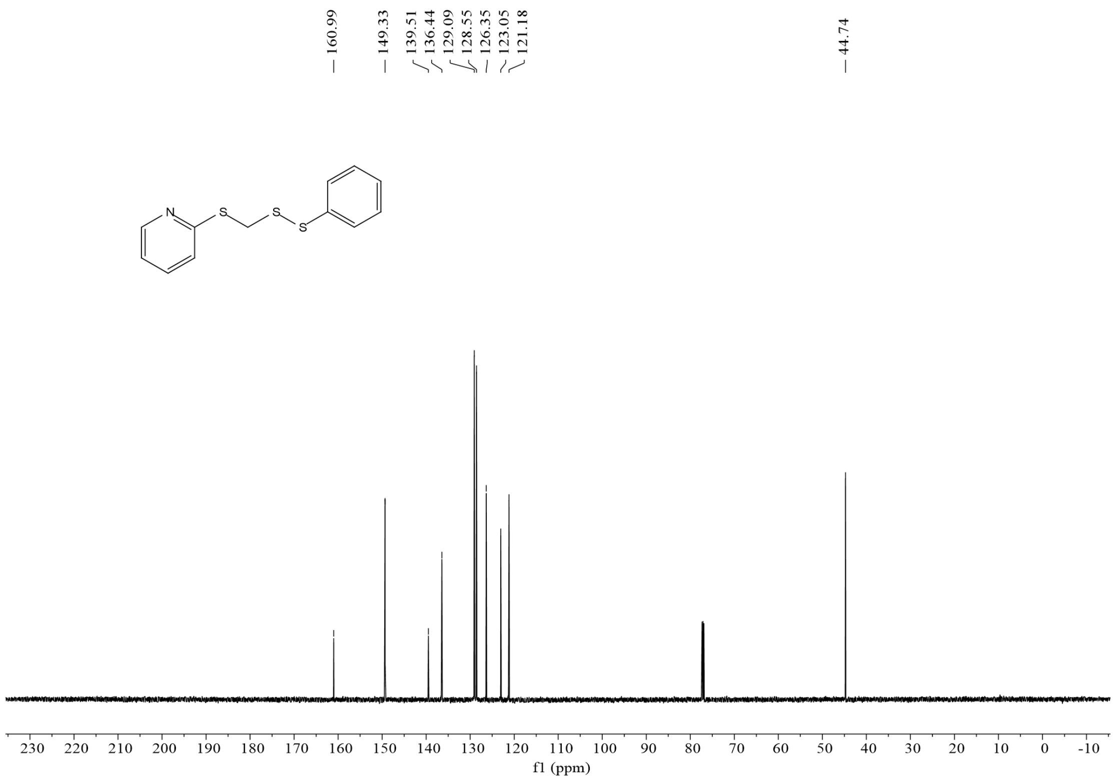

^{13}C NMR of compound 9
